# *S*-Adenosylmethionine–responsive cystathionine β-synthase modulates sulfur metabolism and redox balance in *Mycobacterium* *tuberculosis*

**DOI:** 10.1126/sciadv.abo0097

**Published:** 2022-06-24

**Authors:** Parijat Bandyopadhyay, Ishika Pramanick, Rupam Biswas, Sabarinath PS, Sreesa Sreedharan, Shalini Singh, Raju S. Rajmani, Sunil Laxman, Somnath Dutta, Amit Singh

**Affiliations:** 1 Department of Microbiology and Cell Biology, Indian Institute of Science, Bangalore, Karnataka 560012, India.; 2 Centre for Infectious Disease Research, Indian Institute of Science, Bangalore, Karnataka 560012, India.; 3 Molecular Biophysics Unit, Indian Institute of Science, Bangalore, Karnataka 560012, India.; 4 Institute for Stem Cell Science and Regenerative Medicine, Bangalore, Karnataka 560065, India.

## Abstract

Methionine and cysteine metabolisms are important for the survival and pathogenesis of *Mycobacterium tuberculosis* (*Mtb*). The transsulfuration pathway converts methionine to cysteine and represents an important link between antioxidant and methylation metabolism in diverse organisms. Using a combination of biochemistry and cryo–electron microscopy, we characterized the first enzyme of the transsulfuration pathway, cystathionine β-synthase (*Mtb*Cbs) in *Mtb*. We demonstrated that *Mtb*Cbs is a heme-less, pyridoxal-5′-phosphate–containing enzyme, allosterically activated by *S*-adenosylmethionine (SAM). The atomic model of *Mtb*Cbs in its native and SAM-bound conformations revealed a unique mode of SAM-dependent allosteric activation. Further, SAM stabilized *Mtb*Cbs by sterically occluding proteasomal degradation, which was crucial for supporting methionine and redox metabolism in *Mtb*. Genetic deficiency of *Mtb*Cbs reduced *Mtb* survival upon homocysteine overload in vitro, inside macrophages, and in mice coinfected with HIV. Thus, the *Mtb*Cbs-SAM axis constitutes an important mechanism of coordinating sulfur metabolism in *Mtb*.

## Teaser

SAM-dependent regulation of cystathionine β-synthase governs the directionality of the transsulfuration pathway in *Mtb*.

## INTRODUCTION

*Mycobacterium tuberculosis* (*Mtb*) multiplies, establishes infection, and maintains a state of chronic persistence within the human host (*1*). Moreover, *Mtb* can reinitiate growth after long-term persistence, enabling transmission to a new host and perpetuation of infection (*2*). Studies indicate that the flexibility of metabolic functions limits *Mtb’s* dependence on host metabolites and facilitates survival in the face of host immunity and drug pressures (*3*, *4*). *Mtb* carries out de novo biosynthesis of all essential cofactors and amino acids (*5*). Strains auxotrophic for amino acids are among the most attenuated in vivo (*6*–*8*) and have showed remarkable vaccine potential (*9*–*11*). Therefore, molecular dissection of *Mtb*’s metabolic flexibility is imperative to understand the basis of its success as a human pathogen.

In this context, the metabolism of sulfur (S)–containing amino acids cysteine (Cys) and methionine (Met) is markedly important in *Mtb’s* pathophysiology. Cys functions as a central regulator of redox homeostasis by synthesizing the major mycobacterial antioxidant buffer, mycothiol (*12*). In addition, Cys-derived S is mobilized to generate (Fe-S) clusters (*13*, *14*), sulfolipids (SL-1) (*15*), and hydrogen sulfide (H_2_S) (*16*), crucial for respiration and persistence of *Mtb*, and to tolerate antibiotics (*17*–*20*). Similarly, Met, via the active methyl cycle (AMC), generates the ubiquitous methyl donor, *S*-adenosylmethionine (SAM), which is essential for the biosynthesis of mycolic acids, menaquinone, and biotin (*21*–*23*). Abrogation of Met biosynthesis in *Mtb* diminishes SAM levels, inhibits methylation and one-carbon metabolism, and leads to hyperattenuation in mice (*7*).

In higher eukaryotes such as humans, the metabolism of these two sulfur-containing amino acids is linked to cater to cellular antioxidant and methylation demands (*24*). Met is an essential amino acid and is acquired from the diet, which, in turn, is converted to Cys via the reverse transsulfuration (RTS) pathway. The first and rate-limiting enzyme of the pathway, cystathionine β-synthase (CBS; E.C: 4.2.1.22), a pyridoxal-5′-phosphate (PLP) containing heme protein, condenses serine (Ser) and homocysteine (Hcys), an intermediate of the AMC, to form cystathionine (Cysth). Cysth is subsequently cleaved by cystathionine (Cysth) γ-lyase (CGS; E.C: 4.4.1.1) to yield Cys, which is a precursor of the major cellular antioxidant, glutathione (*25*). Hcys functions as a branch-point metabolite that can either enter the RTS pathway to form Cys during Met sufficiency or get remethylated to form Met and SAM via AMC upon Met deficiency. Hcys partitioning is dependent on CBS and the cellular abundance of its allosteric activator SAM (*26*). Therefore, CBS and SAM in tandem adjust the balance between conserving Met via AMC or committing it to generate antioxidants by RTS to achieve coordinated changes in methylation status or redox potential of the cell, respectively (*27*). In addition, the RTS pathway is also the principal source of the gasotransmitter H_2_S, which has emerged as a major gaseous cellular signaling molecule with cytoprotective effects (*28*).

In contrast to mammals, the biosyntheses of Cys and Met are unlinked and occur de novo in *Mtb* using sulfide (S^2−^) as the S source derived from the reductive sulfate assimilation pathway (*29*). Genetic and pharmacological studies have implicated the reductive S metabolism in redox homeostasis, persistence, and pathogenesis of *Mtb* (*12*, *18*, *30*). Contrary to the reductive arm of S metabolism, the role of RTS pathway in the physiology and pathogenesis of *Mtb* has remained poorly characterized. Therefore, the mechanistic dissection of the functional role of the RTS pathway of *Mtb* is important. Of interest to us is the underlying mechanism(s) that metabolically links RTS pathway with the AMC for maintaining cellular homeostasis in *Mtb*.

In this study, we have characterized the first enzyme of the RTS pathway of *Mtb*, CBS (*Mtb*Cbs) using a range of biochemical, structural [cryo–electron microscopy (cryo-EM)], and genetic approaches. We uncovered a regulatory mechanism by which *Mtb*Cbs is allosterically activated by SAM. A reduction in SAM levels led to destabilization of *Mtb*Cbs posttranslationally, which promoted metabolic switching of the Cys biosynthetic RTS pathway to the Met biosynthetic forward transsulfuration (FTS) pathway. Inhibition of this switching perturbed redox homeostasis and compromised *Mtb’s* survival. Further, genetic disruption of *Mtb*Cbs perturbed survival of *Mtb* in response to Hcys overload in vitro. We showed that under a clinically relevant pathological condition with increased Hcys levels i.e., HIV–tuberculosis (TB) coinfection, *Mtb*Cbs is crucial for the survival of *Mtb*. Our findings establish *Mtb*Cbs as a novel regulatory node in the sulfur metabolism of *Mtb*.

## RESULTS

### *Mtb*Cbs contains PLP and is allosterically activated by SAM

The *Mtb* H37Rv genome encodes a putative CBS (Rv1077, *Mtb*Cbs) (https://mycobrowser.epfl.ch/) belonging to the type II–fold PLP-containing protein family. In contrast to reported bacterial CBSs (*31*), Pfam (protein family database) analysis revealed the presence of the SAM binding Bateman module in *Mtb*Cbs. Purified *Mtb*Cbs eluted as a tetramer based on size exclusion chromatography (SEC) and SEC–multiangle light scattering (SEC-MALS) (fig. S1, A to C). The purified *Mtb*Cbs was yellow in color and exhibited an ultraviolet-visible (UV-VIS) absorption peak at 412 nm, which is characteristic of the protonated internal aldimine form of PLP. The peak at 412 nm was abrogated when the PLP-coordinating active site lysine (K44) was mutated to alanine (K44A) (fig. S1D). *Mtb*Cbs did not exhibit heme-associated Soret peak in the UV-VIS spectrum, and the pyridine hemochromagen assay further confirmed that the protein is heme-less (fig. S1, E to G).

The CBS enzyme from diverse organisms uses multiple substrates to generate Cysth, lanthionine (Lnth), Ser, and H_2_S (table S1) (*32*). Using acid-ninhydrin assay, we found that *Mtb*Cbs generated Cysth from Ser + Hcys, but not from *O*-acetylserine + Hcys as substrates (fig. S1H) (*33*). Further, lead-acetate assay confirmed that *Mtb*Cbs generates H_2_S using Cys or Hcys + Cys but not from Hcys alone (fig. S1I). In addition, using liquid chromatography–tandem mass spectrometry (LC-MS/MS), we confirmed that *Mtb*Cbs produces Lnth from Cys (fig. S2A). Further, *Mtb*Cbs produces Cysth from Ser + Hcys and Cys + Hcys (fig. S2, B to D). However, unlike human CBS (hCBS), it does not generate Ser from Cys. We calculated the steady-state kinetic parameters of the *Mtb*Cbs H_2_S-generating reactions (fig. S3, A to C, and table S2), which revealed the kinetic preference for the condensation of Hcys + Cys over two molecules of Cys.

**Fig. 1.**
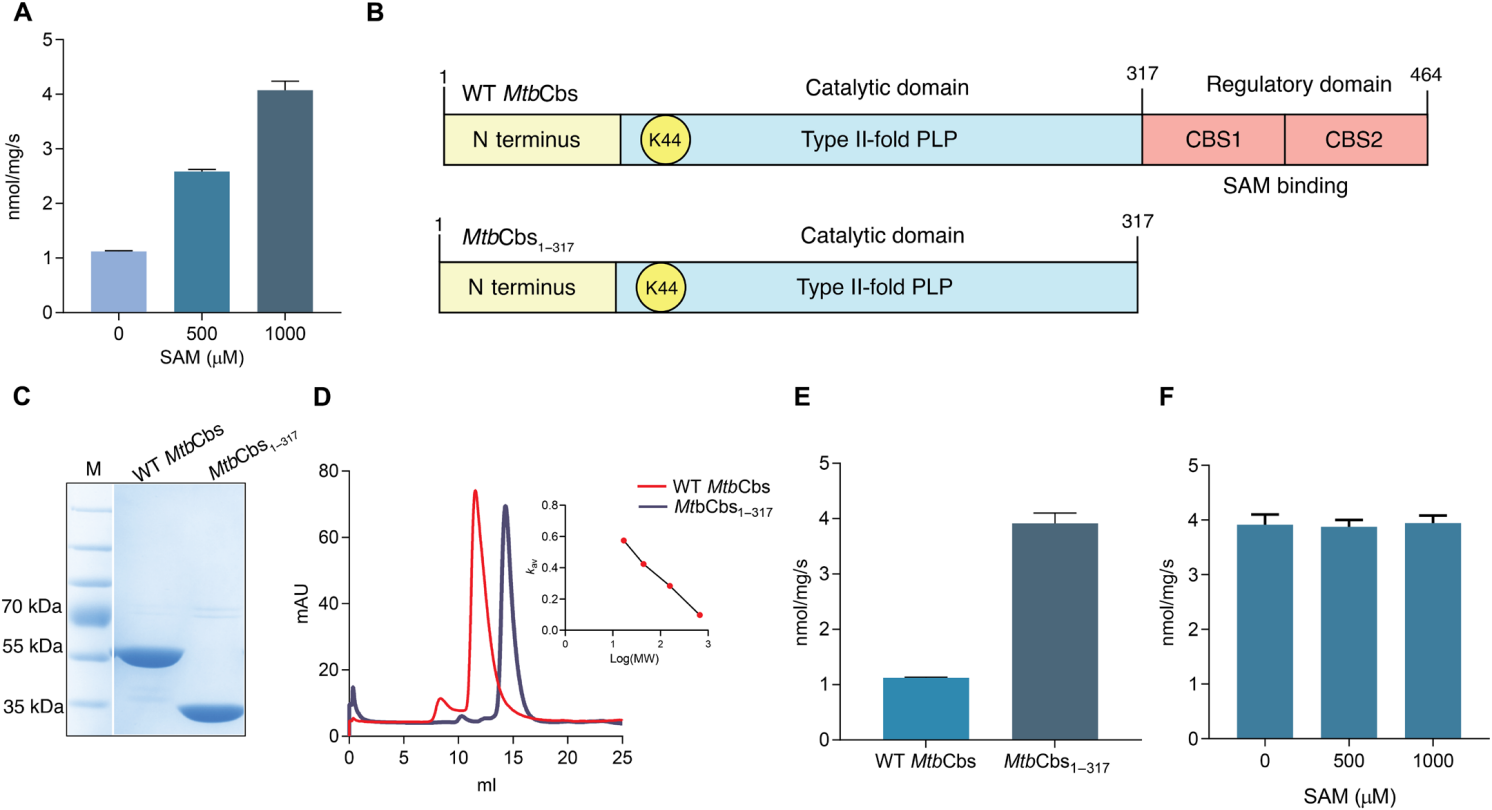
*Mtb*Cbs is allosterically activated by SAM. (**A**) Specific activity of H_2_S production by *Mtb*Cbs in the presence of the indicated amounts of SAM and 20 mM Cys as substrate. (**B**) Schematic diagram of the modular arrangement of WT *Mtb*Cbs and *Mtb*Cbs_1–317_ with C-terminal 147–amino acid deletion. (**C**) SDS–polyacrylamide gel electrophoresis (PAGE) gel of purified MtbCbs and MtbCbs_1-317_. For representation, the marker lane (M) was inserted with the protein lanes. (**D**) SEC profile of Ni-NTA–purified *Mtb*Cbs and *Mtb*Cbs_1–317_. mAU, milli–arbitrary units; MW, molecular weight. (**E**) Specific activities of H_2_S production by *Mtb*Cbs and *Mtb*Cbs_1–317_ showing increased basal activity of *Mtb*Cbs_1–317_. (**F**) *Mtb*Cbs_1–317_ was not activated further in the presence of increasing concentrations of SAM.

Because *Mtb*Cbs harbors the SAM binding Bateman module, we next tested whether SAM allosterically regulates *Mtb*Cbs. We found that SAM, in a concentration-dependent manner, enhanced the H_2_S-producing activity of *Mtb*Cbs by two- to fourfold (Fig. 1A), similar to what has been observed in hCBS (*34*). Under steady-state conditions, SAM increased the *V*_max_ of both the Lnth- and Cysth- generating reactions, with moderate change in the *k*_m_ of Cys, while that of Hcys remained unchanged (fig. S3, D to F, and table S2). We also purified a *Mtb*Cbs mutant lacking the Bateman module (*Mtb*Cbs_1–317_) (Fig. 1, B and C), which eluted as a dimer (*M*_r_ ≅ 65 kDa) (Fig. 1D), exhibiting significantly higher basal specific activity than wild-type (WT) *Mtb*Cbs (Fig. 1E), and was nonresponsive to SAM (Fig. 1F). Under steady-state conditions, the kinetic parameters of H_2_S generation by *Mtb*Cbs_1–317_ were similar to that of WT *Mtb*Cbs in the presence of SAM (fig. S3, G to I, and table S2), confirming that the deletion of the SAM binding domain constitutively activated *Mtb*Cbs. To the best of our knowledge, this is the first report of a bacterial CBS-exhibiting SAM-dependent activation and oligomerization via its SAM binding Bateman module.

### Structure of full-length WT native *Mtb*Cbs

**Fig. 2.**
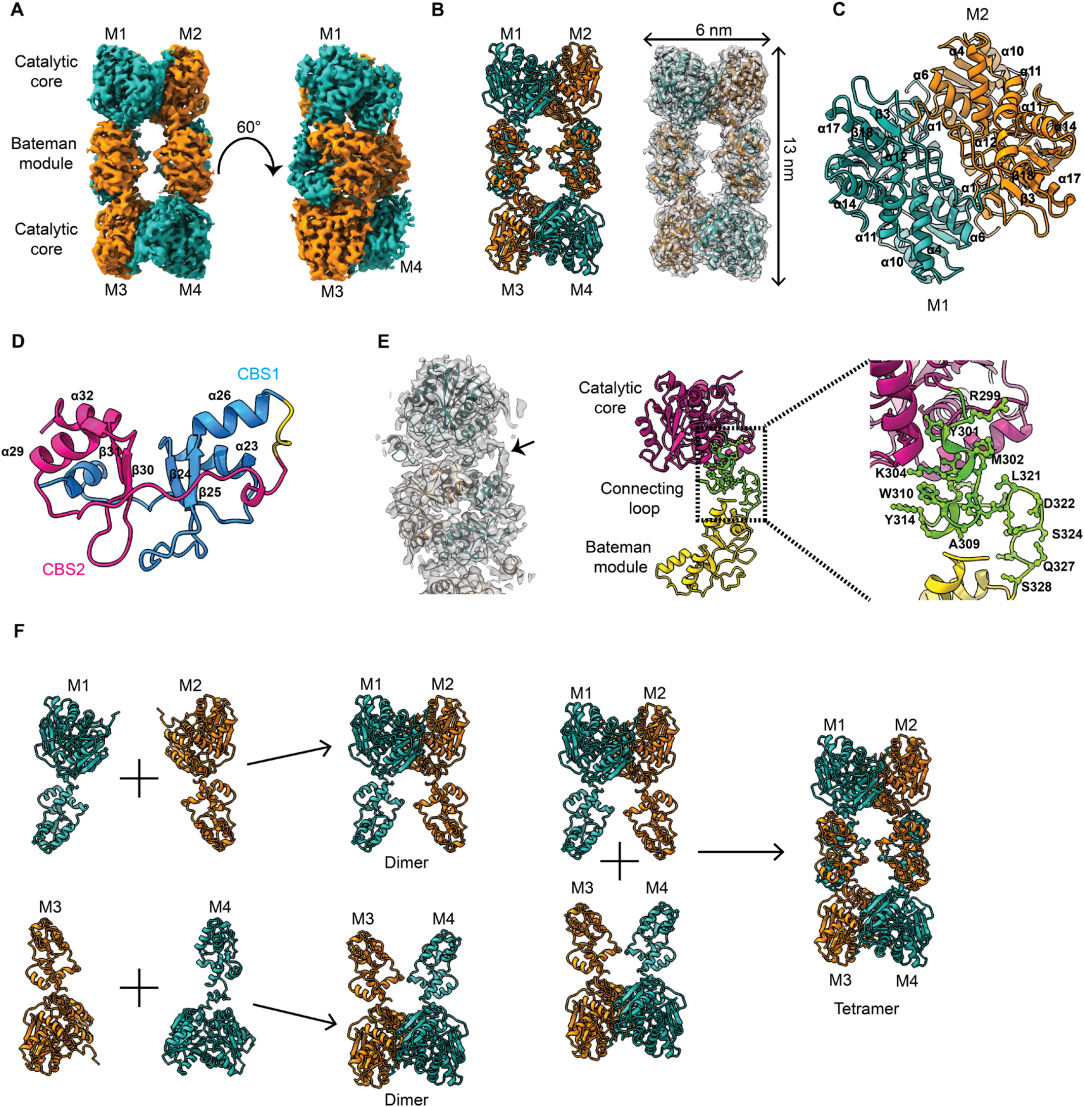
Cryo-EM 3D reconstruction and atomic model building of native *Mtb*Cbs. (**A**) Solid representation of cryo-EM 3D model of native *Mtb*Cbs. Arrangement of four monomers of *Mtb*Cbs is shown in two different colors, green and orange, where the diagonally opposite monomers have the same color. Arrangement of catalytic core and Bateman module is marked. Bateman modules from two monomers interact antiparallelly to form a tetrameric *Mtb*Cbs. Four different monomers are marked M1 to M4. (**B**) Atomic model of tetrameric *Mtb*Cbs in the left panel. Arrangement of four monomers of *Mtb*Cbs is shown in two different colors, green and orange, where diagonally opposite monomers are shown in two different colors. Four different monomers are marked M1 to M4. Transparent representation of *Mtb*Cbs fitted with the atomic model in the right panel. The length of *Mtb*Cbs is 13 nm, whereas breadth is 6 nm. (**C**) Enlarged view of catalytic core region. The helices and β strands are marked. (**D**) Enlarged view of the Bateman module of *Mtb*Cbs, which contains two tandemly repeated CBS motifs (CBS1 and CBS2), colored in blue and pink, respectively. The helices and β strands are marked. (**E**) The connecting loop between catalytic core and Bateman module is shown with black arrowhead. Middle panel shows the atomic model of a monomer of *Mtb*Cbs, where the catalytic core is colored in purple, the Bateman module is colored in yellow, and the connecting loop is colored in green. The right panel showed the enlarged view of connecting loop. The amino acid residues present in the loop region are G297-S328. (**F**) Proposed mechanism for oligomerization of *Mtb*Cbs. M1 and M2 monomeric subunits interact with each other to form dimer, whereas M3 and M4 monomeric subunits interact with each other to form another dimer unit. Two dimeric *Mtb*Cbs units interact to form a tetramer.

Because *Mtb*Cbs was tetrameric and responsive to SAM, we next investigated the structural aspects of its assembly to understand the mechanism of SAM-mediated activation. Single-particle cryo-EM was used to analyze the conformation of the native tetrameric *Mtb*Cbs. Initially, native *Mtb*Cbs was observed by room-temperature negative staining transmission electron microscopy (TEM) to identify the overall shape and stability of the complex. Negative staining TEM micrographs and reference-free two-dimensional (2D) class averages revealed a rectangular-shaped tetrameric architecture of native *Mtb*Cbs (fig. S4A). The same sample was used to perform cryo-EM imaging (fig. S4B), and the atomic-resolution 3D reconstruction of the *Mtb*Cbs homotetramer was resolved at 3.6 Å, with the core domain resolved at 3.1 Å (Fig. 2A and figs. S4, C to E, and S5A). The rectangular-shaped 3D structure of the tetrameric *Mtb*Cbs contains an N-terminal catalytic core region (7 to 296 residues), C-terminal Bateman module (329 to 464 residues), and a connecting linker between the catalytic core and the Bateman module (297 to 328 residues) (Fig. 2, A and B). The stable catalytic core of one monomer is firmly associated with the catalytic core of another monomer in a head-to-head fashion mainly through hydrophobic interactions (M1 and M2; M3 and M4), with a total dimeric surface area of 1882 Å^2^ (Fig. 2C and fig. S5B). The Bateman module is composed of two CBS motifs, CBS1 (T329-E397) and CBS2 (A403-E459), existing in tandem repeats (Fig. 2D). Similar kind of tandem repeats have previously been reported in hCBS (*35*).The Bateman module is positioned outmost from the catalytic core region and is connected to the core domain by a 32–amino acid linker (^297^GGRGYMSKIFNDAWMSSYGFLRSRLDGSTEQS^328^) (Fig. 2E). According to our structural analysis, the C-terminal Bateman module of one dimeric *Mtb*Cbs engages through hydrophobic interactions, salt bridges and H-bonding with another Bateman module of a dimer, in an antiparallel head-to-tail arrangement, resulting in the tetrameric conformation (M1 and M3; M2 and M4) (Fig. 2F and fig. S5B). The tetramer interface has a total surface area of 1779 Å^2^. The overall structure of the N-terminal catalytic core domain of *Mtb*Cbs is quite similar to that of hCBS and *Drosophila* CBS (fig. S6, A and B) (*36*, *37*). Similarly, the Bateman module is quite identical to hCBS (fig. S6A) (*36*). However, a significant difference is observed in the connecting linker and the position of the Bateman module with respect to its corresponding catalytic core domain. In hCBS, the Bateman module is juxtapositioned with the catalytic core, with the Bateman module of the M1 monomer leaning toward the catalytic core domain of M2 monomer (fig. S6A). Unlike other CBS enzymes, significantly in *Mtb*Cbs, the Bateman module is shifted toward the periphery of the homotetramer through the movement of the connecting linker (fig. S6C). The connecting linker helps in the movement of Bateman module, which facilitates the tetrameric assembly of *Mtb*Cbs (Fig. 2F). The density of the connecting linker is well ordered and stable in our cryo-EM structure, which suggests the absence of any disordered amino acid residues (Fig. 2E).

The *Mtb*Cbs catalytic core domain is composed of 10 helices (α1, α4, α6, α8, α10, α11, α12, α14, α16, and α17) and eight β strands (β2, β3, β5, β7, β9, β13, β15, and β18) (fig. S7A). The helices (α1, α6, α8, α12, α16, and α19) of the core domain of one monomer strongly interact with the helices (α1, α6, α8, α12, α16, and α19) of the adjacent monomer to form a stable dimer (fig. S7, B to D). Furthermore, the tetramer interface is stabilized by the interaction of four helices (α20, α23, α26, and α32) present at the Bateman module of two antiparallelly placed monomers (fig. S8A). On the basis of the permissible bond length distance study, we predicted five key amino acid residues, E388, R450, I357, L454, and S393, to be responsible for tetramerization (fig. S8B). To validate this, we performed site-directed mutagenesis of these residues (E388A, R450A, I357A, L454A, and S393R) and analyzed the oligomeric status using SEC (fig. S8C). SEC profile revealed increased propensity of *Mtb*Cbs mutants (E388A, R450A, I357A, L454A, and S393R) to remain in the dimeric state. These mutations at the Bateman module along with its complete deletion (*Mtb*Cbs_1–317_) confirmed our speculation of the oligomerization mechanism, which implicates Bateman module in the dimer to tetramer conversion of *Mtb*Cbs (Fig. 2F and fig. S8C). A major peak shift was observed in the SEC profile of the I357A mutant (fig. S8C). Further, negative staining reference-free 2D classifications of I357A showed the presence of a mixture of dimeric and tetrameric species (fig. S8D). Because the tetramerization state of *Mtb*Cbs also affects its basal activity and SAM responsiveness, specific activities of all the mutants were measured in the presence of SAM using Cys as a substrate. The basal specific activity of I357A (2.16 ± 0.11 nmol/mg per second) was twofold higher than that of WT *Mtb*Cbs (1.03 ± 0.15 nmol/mg per second) with no further SAM-mediated activation (fig. S8, E and F). Whereas for E388A and R450A mutants, the activity was severely compromised, the other two mutants, S393R and L454A, showed moderate activity with subtle change in the rate with increasing concentration of SAM (fig. S8, E and F).

### Conformational changes of *Mtb*Cbs in the presence of SAM and serine

**Fig. 3.**
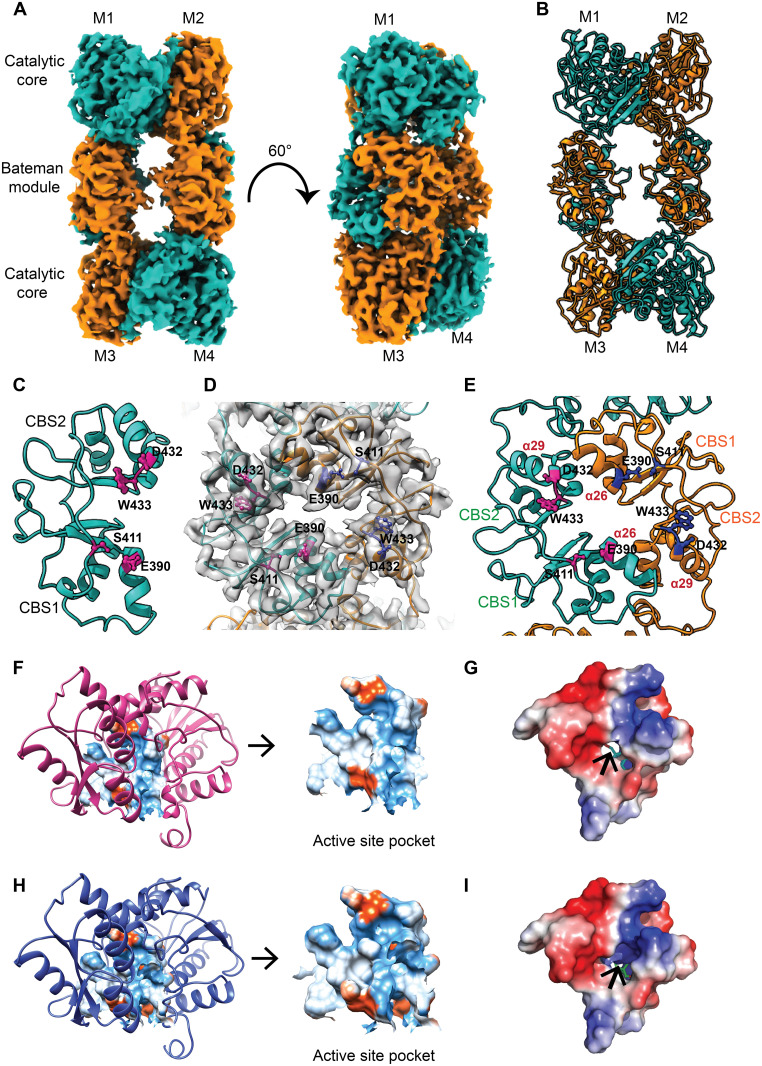
Cryo-EM reconstruction and atomic model building of *Mtb*Cbs in the presence of allosteric activator SAM. (**A**) Solid representation of cryo-EM 3D model of *Mtb*Cbs in the presence of SAM. Arrangement of four monomers is shown in two different colors, green and orange, where the diagonally opposite monomers have the same color. Arrangement of catalytic core and Bateman module is marked, which indicates similar structural arrangement like native *Mtb*Cbs. (**B**) Atomic model of tetrameric *Mtb*Cbs in the presence of SAM. Four monomers in the atomic model are shown in two different colors, green and orange, where the diagonally opposite monomers have the same color. (**C**) Identification of key residues (W433, E390, S411, and D432) in *Mtb*Cbs responsible for SAM binding. (**D**) Enlarged transparent representation fitted with atomic model of the *Mtb*Cbs Bateman module. Two different monomeric chains are colored in green and orange. (**E**) Atomic model of the Bateman module marked with SAM binding amino acid residues in magenta and blue colors in each chain, respectively. (**F**) Determination of substrate groove pocket and position of the pocket in native *Mtb*Cbs. The pocket property is marked with the electrostatic potential value. Red color signifies negative potential, white near neutral, and blue positive potential. (**G**) Electrostatic potential calculated for active site of native *Mtb*Cbs. Substrate channel opening is shown with a black arrow. (**H**) Determination of substrate groove pocket and position of the pocket in SAM-treated *Mtb*Cbs. Electrostatic potential values indicate the surface changes of the amino acids present in the pocket. Red color signifies negative potential, to white near neutral, and to blue for positive potential. The redistribution of surface charges changes after SAM treatment due to the rearrangement of amino acids of SAM binding loop. (**I**) Electrostatic potential calculated for active site of SAM-treated *Mtb*Cbs. Substrate channel opening increases because of allosteric activation of the enzyme (marked with a black arrow).

Similar approaches were implemented to unravel the conformational changes of *Mtb*Cbs in the presence of SAM, and the 3D structure was determined at a resolution of 3.56 Å, with the core domain resolved at 2.8 Å (Fig. 3, A and B, and fig. S9). SAM-treated *Mtb*Cbs retained its rectangular shaped tetrameric assembly similar to native *Mtb*Cbs (fig. S9, A and B). However, some significant differences between these two structures were observed with substantial conformational changes occurring at the Bateman module of SAM-treated *Mtb*Cbs and at the opening of the substrate channel. Because the EM density of SAM was missing in the CBS domain at the root mean square deviation (RMSD) value of 6σ, we predicted the SAM binding position at a low RMSD value of 3σ and pinpointed the probable SAM binding amino acid residues (fig. S10, A and B). Previous studies with hCBS demonstrated the role of F443, Q445, D538, and T535 amino acid residues in SAM binding (fig. S10C) (*36*). Upon superimposition of the Bateman modules of hCBS and *Mtb*Cbs (fig. S10D), we predicted E390, S411, D432, and W433 as the key amino acid residues involved in SAM interaction in *Mtb*Cbs (Fig. 3, C and D). Among these, W433 forms stabilizing stacking interactions with the purine ring of SAM. To validate this, site-directed mutagenesis of these amino acid residues (E390A, S411A, D432N, and W433F) was performed (Fig. 3E). All the mutants retained their tetrameric oligomeric organization but showed significant decrease in SAM-dependent activation, with W433F mutant being the most severely affected (fig. S10E). To rule out the possibility that the decrease in SAM-dependent activation of W433F resulted from enzyme instability, we performed fluorescence thermal shift assay of WT *Mtb*Cbs and W433F. We did not observe any shift in the thermal profile and in the melting transition temperature (*T*_m_) of WT *Mtb*Cbs (57.3° ± 0.592°C) and W433F (58.06° ± 0.55°C), thus indicating that the W433F mutation does not influence enzyme stability (fig. S11A). We further performed microscale thermophoresis assay to ascertain the effect of W433F mutation on SAM binding affinity. The WT *Mtb*Cbs displayed a binding affinity (*k*_d_) of ~128 μM, which was reduced to ~300 μM in W433F (fig. S11, B and C). This can be attributed to the disruption of stacking interaction between SAM and W433F. As expected, *Mtb*Cbs_1–317_, which lacks the C-terminal SAM binding domain, did not display any SAM binding (fig. S11D). Together, these findings identified the amino acid residues involved in SAM binding and SAM-dependent activation.

After SAM treatment, significant changes were observed in the substrate channel of the active site K44. In all the CBS orthologs, the active site invariable lysine is highly conserved. In native *Mtb*Cbs, the active site K44 is buried inside the core region by a flexible loop (G40, G41, S42, S43, D45, and R46), making K44 relatively inaccessible to the substrates. After SAM incubation, the active site K44 gets exposed because of the displacement of the amino acids near the active site region and the substrate channel expands (Fig. 3, F to I). Upon allosteric activation by SAM, this rearrangement of the amino acids occurs to accommodate the higher influx of the substrates toward the active site. These observations revealed the structural basis of SAM-mediated allosteric activation of *Mtb*Cbs.

**Fig. 4.**
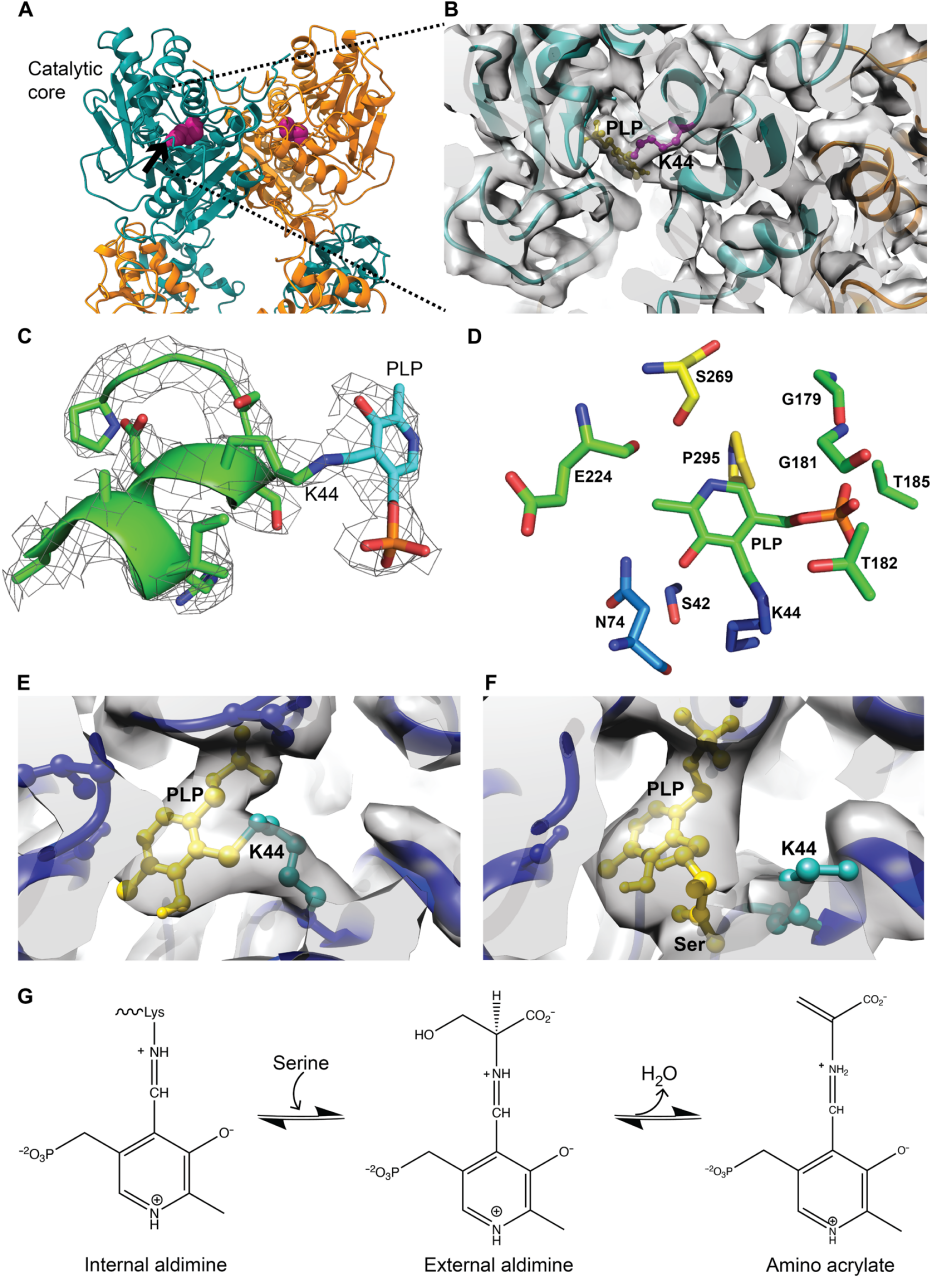
Active site of *Mtb*Cbs and conformational changes after SAM and serine treatment. (**A**) Position of active site K44 in the catalytic core region. (**B**) Zoomed-in view of the active site indicates the presence of PLP. (**C**) A stable continuous density is present between K44 and PLP (fitted at an RMSD value of 6σ). (**D**) Interaction of different amino acids and PLP at the active site groove. (**E**) A continuous density between K44 and PLP indicates the formation of internal aldimine at the active site in the SAM-treated *Mtb*Cbs model (fitted at an RMSD value of 6σ). (**F**) The release of K44 and PLP interaction and the disappearance of continuous density between K44 and PLP indicate the formation of external aldimine/aminoacrylate in the presence of serine (fitted at an RMSD value of 6σ). (**G**) Serine binding converts protonated internal aldimine form to external aldimine and aminoacrylate intermediates.

The active site lysine, K44, is located inside the catalytic core domain on helix α4 and forms a Schiff base interaction with PLP (Fig. 4, A to C). PLP is involved in the interaction with other amino acids (S42, N74, G181, G183, T182, T185, and S269). Our 3D structure shows that S269, G181, G183, T182, and T185 are involved in hydrogen bonding (Fig. 4D).

As the substrate channel expands after SAM treatment, our next target was to observe the changes after SAM and substrate Ser incubation. 3D structure of *Mtb*Cbs retains its tetrameric arrangement after SAM + Ser incubation, and the 3D reconstruction was resolved at a resolution of 4.25 Å (figs. S12 and S13). In the absence of Ser, a stable continuous density is present in the cryo-EM map between K44 and PLP at an RMSD value of 6σ. This indicates the formation of internal aldimine at the active site (Fig. 4E). However, substrate-treated cryo-EM map demonstrated that the interaction between K44 and PLP is disrupted after Ser treatment, followed by the formation of external aldimine/aminoacrylate at the active site, which indicates association of Ser with PLP (Fig. 4, F and G), in agreement with previous reports (*34*, *38*), which is strongly supported by our structural studies.

### SAM stabilizes *Mtb*Cbs by occluding proteasomal degradation

SAM is known to confer stability to hCBS in addition to allosteric activation (*27*). To investigate whether SAM stabilizes *Mtb*Cbs in vitro, we performed the pulse proteolysis assay (*39*) using thermolysin under denaturing conditions. In presence of 500 μM SAM, the *C*_m_ value (urea concentration required to unfold half of *Mtb*Cbs) increased from 3.7 ± 0.3 to 4.92 ± 0.55 M. The global stability of *Mtb*Cbs, Δ*G*°_unf_, increased from 21.2 ± 0.2 to 30.34 ± 0.91 kcal/mol (fig. S14 and table S4). Hence, binding of SAM provides significant stabilization to *Mtb*Cbs. We also performed the pulse proteolysis assay of the SAM unresponsive E390A, D432A, D432N, and W433F mutants and found that the presence of SAM did not confer protection from thermolysin-mediated proteolysis (fig. S15 and table S4).

**Fig. 5.**
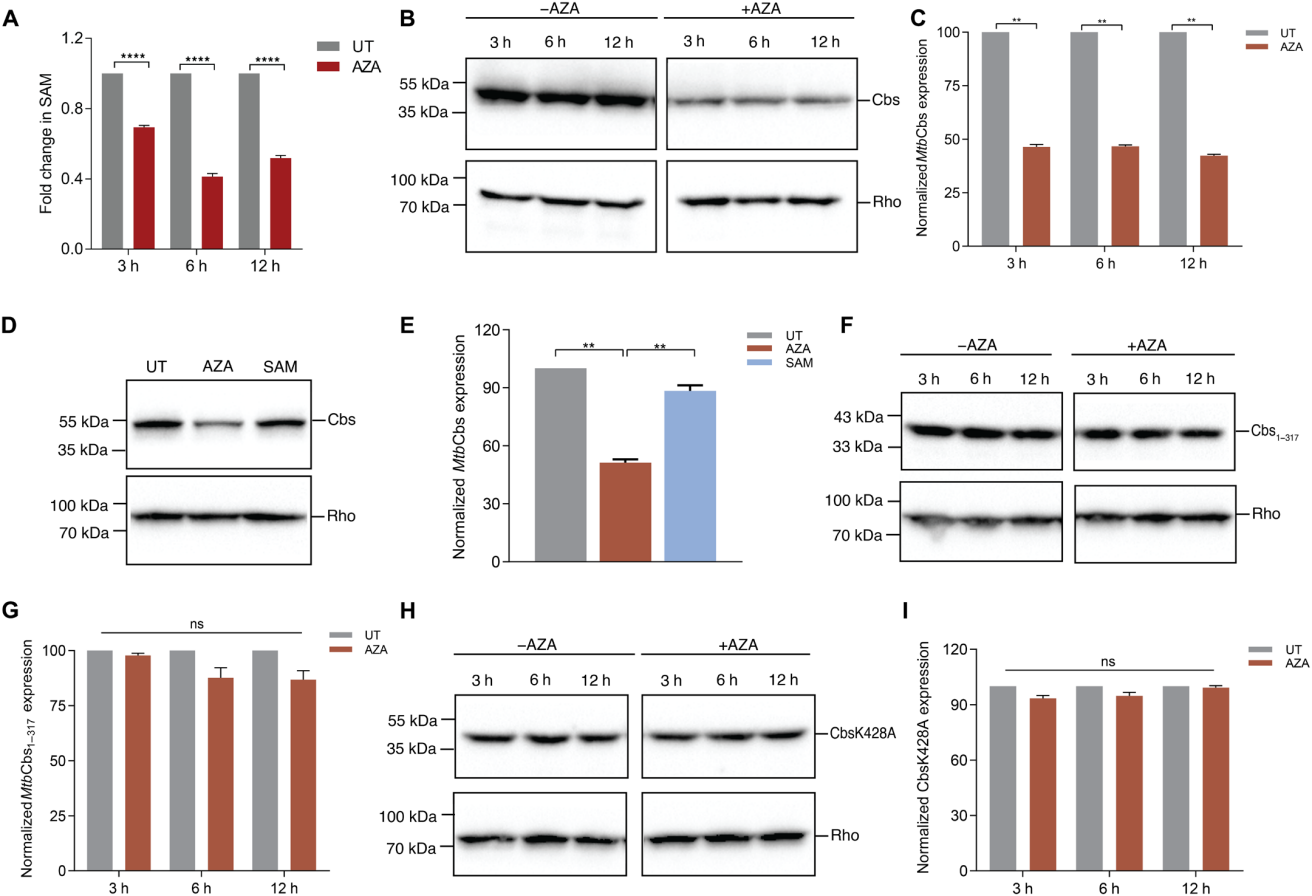
SAM stabilizes *Mtb*Cbs in vivo by occluding proteasomal degradation. (**A**) Exponentially growing *Mtb* was treated with 1 mM AZA for the indicated time periods. ****P < 0.0001 by unpaired *t* test. Data represent three independent biological replicates. (**B**) Immunoblotting of *Mtb*Cbs showing protein abundance upon treatment with 1 mM AZA for the indicated time periods and (**C**) the corresponding densitometry profile. *Mtb*Cbs expression was normalized to the expression of the internal control Rho. Data represent means ± SD of two independent biological replicates. **P = 0.001 by unpaired *t* test. (**D**) Immunoblotting of *Mtb*Cbs showing protein abundance upon treatment with 1 mM AZA for 3 hours, followed by addition of 1 mM SAM for additional 3 hours and (**E**) the corresponding densitometry profile. *Mtb*Cbs expression was normalized to the expression of the internal control Rho. Data represent means ± SD of two independent biological replicates. **P = 0.0033 by unpaired *t* test. (**F**) Immunoblotting of *Mtb*Cbs_1–317_ showing protein abundance upon treatment with 1 mM AZA for the indicated time periods and (**G**) the corresponding densitometry profile. *Mtb*Cbs_1–317_ expression was normalized to the expression of the internal control Rho. Data represent means ± SD of two independent biological replicates. ns, not significant. (**H**) Immunoblotting of *Mtb*CbsK428A showing protein abundance upon treatment with 1 mM AZA for the indicated time periods and (**I**) the corresponding densitometry profile. *Mtb*CbsK428A expression was normalized to the expression of the internal control Rho. Data represent means ± SD of two independent biological replicates. UT, untreated.

After establishing that SAM stabilizes *Mtb*Cbs in vitro, we were interested to understand how intracellular SAM abundance regulates its stability in vivo. In *Mtb*, SAM is synthesized by the enzyme SAM synthetase (MetK; Rv1392). A purine analog, azathioprine (AZA) is a well-established inhibitor of *Mtb* MetK (*40*). We confirmed that treatment with 1 mM AZA resulted in depletion of SAM in a time-dependent manner (Fig. 5A). Concomitantly, cellular *Mtb*Cbs protein levels also reduced (*P* = 0.0035) (Fig. 5, B and C) without affecting the *cbs* transcript (fig. S16A). Exogenous supplementation with 1 mM SAM for additional 3 hours rescued AZA-mediated down-regulation of *Mtb*Cbs (Fig. 5, D and E) and restored intracellular SAM levels (fig. S16B). We genetically expressed the E390A, S411A, D432A, and W433F mutants in the *Mtb* strain lacking WT *Mtb*Cbs (*Mtb*Δ*cbs*) and found that SAM failed to rescue the down-regulation of these proteins upon AZA treatment (fig. S17, A to H). Furthermore, we also expressed *Mtb*Cbs_1–317_ in *Mtb*Δ*cbs* (*Mtbcbs*_1–317_) and showed that deletion of the Bateman domain rendered it insensitive to AZA-mediated down-regulation (Fig. 5, F and G). This indicated the existence of a regulatory motif in the C terminus of *Mtb*Cbs that is crucial for SAM-dependent stabilization of *Mtb*Cbs.

Recently, *Mtb*Cbs was reported to have a pupylation site at lysine-428 (K428), which likely facilitates prokaryotic ubiquitin-like protein (Pup)–mediated proteasomal degradation of mycobacterial proteins (*41*). Because K428 lies in the SAM binding cleft of the Bateman module, we proposed an “occlusion by occupation model” to explain SAM-dependent degradation of *Mtb*Cbs. Under SAM sufficiency, SAM binding likely precludes protein degradation by sterically hindering the access of K428 for pupylation. Under SAM limitation, the exposed K428 of *Mtb*Cbs is pupylated and targeted for proteasomal degradation. Consistent with this, expression of a pupylation-deficient K428A mutant in *Mtb*Δ*cbs* (*MtbcbsK428A*) prevented its degradation upon AZA treatment (Fig. 5, H and I). Together, we show that *Mtb*Cbs is sensitive to intracellular SAM and is likely targeted for proteasomal degradation during SAM limitation.

### *Mtb*Cbs down-regulation reroutes transsulfuration toward SAM replenishment

**Fig. 6.**
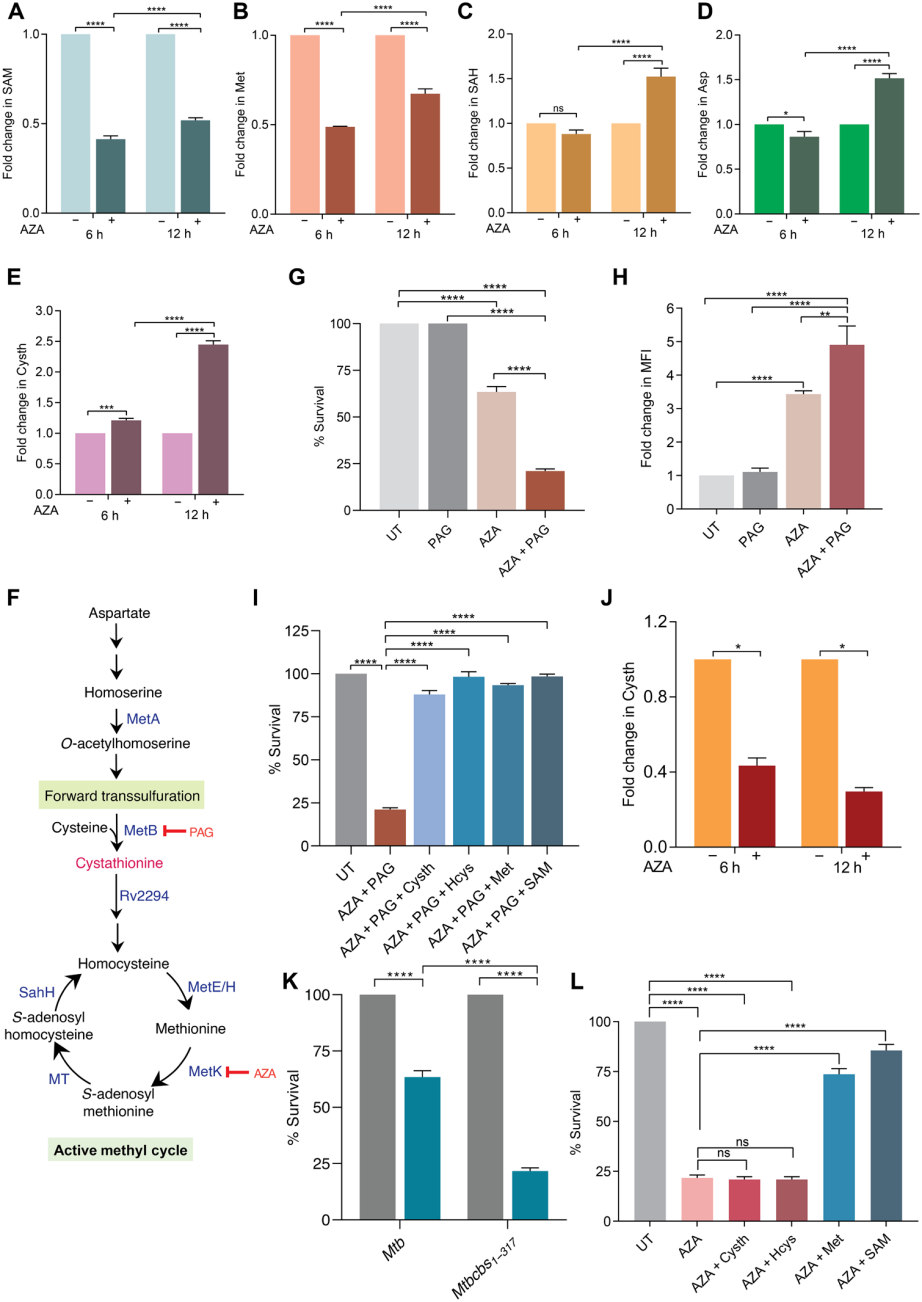
SAM depletion signals resetting of the transsulfuration pathway in *Mtb*. (**A** to **E**) Fold change in abundance of RTS and AMC intermediates upon treatment with 1 mM AZA for the indicated time periods. Data represent means ± SD of three independent biological replicates. *P = 0.0114, ***P = 0.0005, and ****P < 0.0001 by two-way analysis of variance (ANOVA) with Tukey’s multiple comparison test. (**F**) Schematic showing the FTS pathway of methionine biosynthesis. MetA, homoserine *O*-acetyltransferase; MetB, bifunctional Cysth γ-synthase/β-lyase; Rv2294, probable Cysth β-lyase; MetE, 5-methyltetrahydropteroyltriglutamate–homocysteine methyltransferase; MetH, 5-methyltetrahydrofolate–homocysteine methyltransferase; MetK, SAM synthetase; MT, methytransferase; SahH, *S*-adenosylhomocysteine hydrolase. (**G**) Exponentially growing *Mtb* was treated with 1 mM AZA alone or in combination with 500 μM PAG for 24 hours. (**H**) ROS was detected (at 12 hours after treatment) by staining with CellROX DeepRed and analyzed by flow cytometry with λ_Excitation_ = 644 nm and λ_Emission_ = 665 nm. MFI, mean fluorescence intensity. (**I**) Rescue of the survival defect caused by cotreatment with AZA + PAG. Hcys, Cysth, Met, and SAM were added 6 hours after treatment with AZA + PAG at a final concentration of 1 mM each. (**J**) Fold change in Cysth abundance of *Mtb*cbs_1–317_ upon treatment with 1 mM AZA for 6 and 12 hours. (**K**) Exponentially growing *Mtb*Cbs_1–317_ strain was treated with 1 mM AZA for 24 hours. (**L**) Rescue of the survival defect caused by AZA treatment in *Mtb*Cbs_1–317_ strain. Hcys, Cysth, Met, and SAM were added 6 hours after treatment with AZA + PAG at a final concentration of 1 mM each. For all survival experiments, percent survival was calculated by colony-forming unit (CFU) enumeration on 7H11-ADS plates. (G to L) Data represent means ± SD of three independent biological replicates. *P = 0.0116, **P = 0.0012, ****P < 0.0001 by one-way ANOVA with Tukey’s multiple comparison test.

We next interrogated the effect of SAM-dependent allosteric regulation of *Mtb*Cbs on AMC and RTS pathway of *Mtb*. We measured the abundance of the RTS pathway and AMC intermediates upon AZA-mediated SAM depletion by targeted metabolomics. Treatment of *Mtb* with 1 mM AZA for 6 to 12 hours led to a reduction in SAM (*P* < 0.0001) and Met (*P* < 0.0001) levels at 6 hours after treatment with AZA (Fig. 6, A to C). In comparison to 6 hours, 12 hours of AZA treatment showed signs of recovery in the levels of SAM (*P* < 0.0001), Met (*P* < 0.0001), and S-adenosylhomocysteine (SAH) (*P* < 0.0001) (Fig. 6, A to C). Aspartate (Asp), which is the main precursor of Met biosynthesis, also showed significant (*P* < 0.0001) increase at 12 hours after treatment with AZA (Fig. 6D). These results suggested that *Mtb* likely responds to inhibition of SAM-*Mtb*Cbs axis by initially down-regulating Met biosynthesis, followed by a gradual recovery in the intracellular levels of AMC intermediates.

Down-regulation of *Mtb*Cbs is expected to reduce the intracellular levels of Cysth. However, contrary to our expectations, we observed significant increase in Cysth levels at 6 and 12 hours (*P* < 0.0001) after AZA treatment, respectively, as compared to untreated controls (Fig. 6E). Cysth is also an intermediate of the FTS pathway, which converts Cys to Met in bacteria such as *Escherichia coli* and *Salmonella typhimurium* (*42*). In the FTS pathway, Cysth γ-synthase (CGS) condenses *O*-acetyl homoserine and Cys to form Cysth, which is then cleaved by Cysth β-lyase (CBL) to form Hcys for Met and SAM biosynthesis (Fig. 6F). Because we observed increased Cysth abundance upon SAM depletion, one likely possibility is that *Mtb* activates the FTS pathway of Met biosynthesis. *Mtb* encodes a bifunctional enzyme, MetB (Rv1079), which has both CGS and Cysth γ-lyase (CGL) activities (*43*) and therefore could generate Cysth to fuel Met biosynthesis and maintain viability in response to AZA. To test this, we treated *Mtb* with AZA for 3 hours alone or followed by exogenous supplementation with 1 mM SAM for an additional 3 hours. We found that Cysth levels increased only in the presence of AZA (*P* < 0.0001), while exogenous SAM addition reversed it to the level of untreated control (fig. S18).

We also measured the survival of *Mtb* upon treatment with the MetB inhibitor, propargylglycine (PAG; 500 μM) in combination with 1 mM AZA. While 1 mM AZA reduced *Mtb’s* survival by 40% (*P* < 0.0001), PAG alone was ineffective against *Mtb*. However, cotreatment with AZA and PAG led to 90% decrease (*P* < 0.0001) in *Mtb*’s survival as compared to the untreated control (Fig. 6G). Disruption of Met biosynthesis kills *Mtb* by inducing oxidative stress (*7*). Consistent with this, reactive oxygen species (ROS) levels were induced to 3.5-fold (*P* < 0.0001) upon treatment with AZA, which further increased to 5.0-fold (*P* = 0.012) in response to AZA + PAG combination (Fig. 6H). Exogenous supplementation with Cysth, Hcys, or Met rescued the growth defect and improved the survival of *Mtb* in response to AZA + PAG combination (Fig. 6I). Both the transcript and protein levels of MetB remained unchanged upon AZA treatment (fig. S19, A to C), suggesting that *Mtb* MetB is able to functionally switch from CGL to CGS upon aberration in *Mtb*Cbs-SAM axis. We propose that under SAM-sufficient condition, *Mtb*Cbs is stabilized and converts Hcys to Cysth. This kinetically constrains MetB to function as a CGL to generate Cys from Cysth via the RTS pathway. However, upon SAM depletion and concomitant destabilization of *Mtb*Cbs, MetB exerts its CGS activity to generate Cysth via the FTS pathway for replenishment of Met and SAM. These findings indicate that the induction of the FTS pathway via CGS activity of MetB is likely to be dependent on the concomitant depletion of both SAM and *Mtb*Cbs. Consistent with this, the CBS-deficient strain of *Mtb* (*Mtb*Δ*cbs*) did not show any increase in Cysth levels upon AZA treatment (fig. S20), reiterating that *Mtb*Cbs down-regulation in response to SAM depletion is prerequisite for activating FTS pathway. To further assess this, we examined the effect of AZA treatment on *Mtbcbs*_1–317_. Because it constitutively expresses CBS activity in a SAM-independent manner, MetB would be coerced to function as a CGL rather than a CGS even under SAM-deficient conditions. Consistent with this, Cysth levels did not increase in AZA-treated *Mtbcbs*_1–317_ (Fig. 6J). Furthermore, AZA-treated *Mtbcbs*_1–317_ showed a significant growth defect as compared to WT *Mtb*, indicating the inability of the strain to sustain CGS activity of MetB for maintaining essential AMC intermediate pools (Fig. 6K). In agreement with this, exogenous supplementation of Met or SAM, but not Hcys or Cysth, restored the survival of *Mtbcbs*_1–317_ in response to AZA (Fig. 6L). In summary, *Mtb*Cbs-SAM axis constitutes a novel regulatory node in the Cys-Met metabolism and proffers functional significance to the reversibility of the transsulfuration pathway in *Mtb*.

### Antifolate drugs destabilize *Mtb*Cbs to affect survival in *Mtb*

Antifolate antibiotics such as *para*-amino salicylic acid (PAS) and sulfamethoxazole (SMX) have been reported to deplete critical AMC intermediates, including SAM, in *Mtb* (*44*). On the basis of this, we next checked the effect of antibiotics PAS and SMX on *Mtb*Cbs stability. We treated WT *Mtb* with 1×, 5×, and 10× MIC (minimum inhibitory concentration) of PAS and SMX for 12 hours MIC was determined as described previously (*20*). We found that PAS and SMX treatment led to *Mtb*Cbs down-regulation, which was maximum at 10× MIC of both the antibiotics (fig. S21, A and B). As expected, the *Mtb*Cbs levels in SAM-unresponsive *Mtbcbs*_1–317_ and pupylation-resistant *MtbcbsK428A* remained unchanged (fig. S21, C to F). Further, we examined the sensitivity of WT *Mtb*, *Mtb*Δ*cbs*, *Mtbcbs-comp*, *Mtbcbs*_1–317_, and *MtbcbsK428A* toward PAS and SMX. We found that *Mtbcbs*_1–317_ showed a two- and fourfold decrease in MIC_90_ of PAS and SMX, respectively, as compared to *Mtb*Δ*cbs* and *Mtbcbs-comp* (fig. S22, A to C). In addition, MIC_90_ of PAS and SMX was found to be twofold lower in *MtbcbsK428A* as compared to WT, *Mtb*Δ*cbs*, and *Mtbcbs-comp* (fig. S22, A to C). These findings support our observations that under SAM-depleted condition, survival of *Mtb* is dependent on Cbs down-regulation and activation of the FTS pathway. Thus, the Cbs-SAM axis plays an important role in *Mtb*’s survival against antifolate antibiotics.

### *Mtb*Cbs confers protection from Hcys toxicity and affects survival of *Mtb* during HIV-TB coinfection

**Fig. 7.**
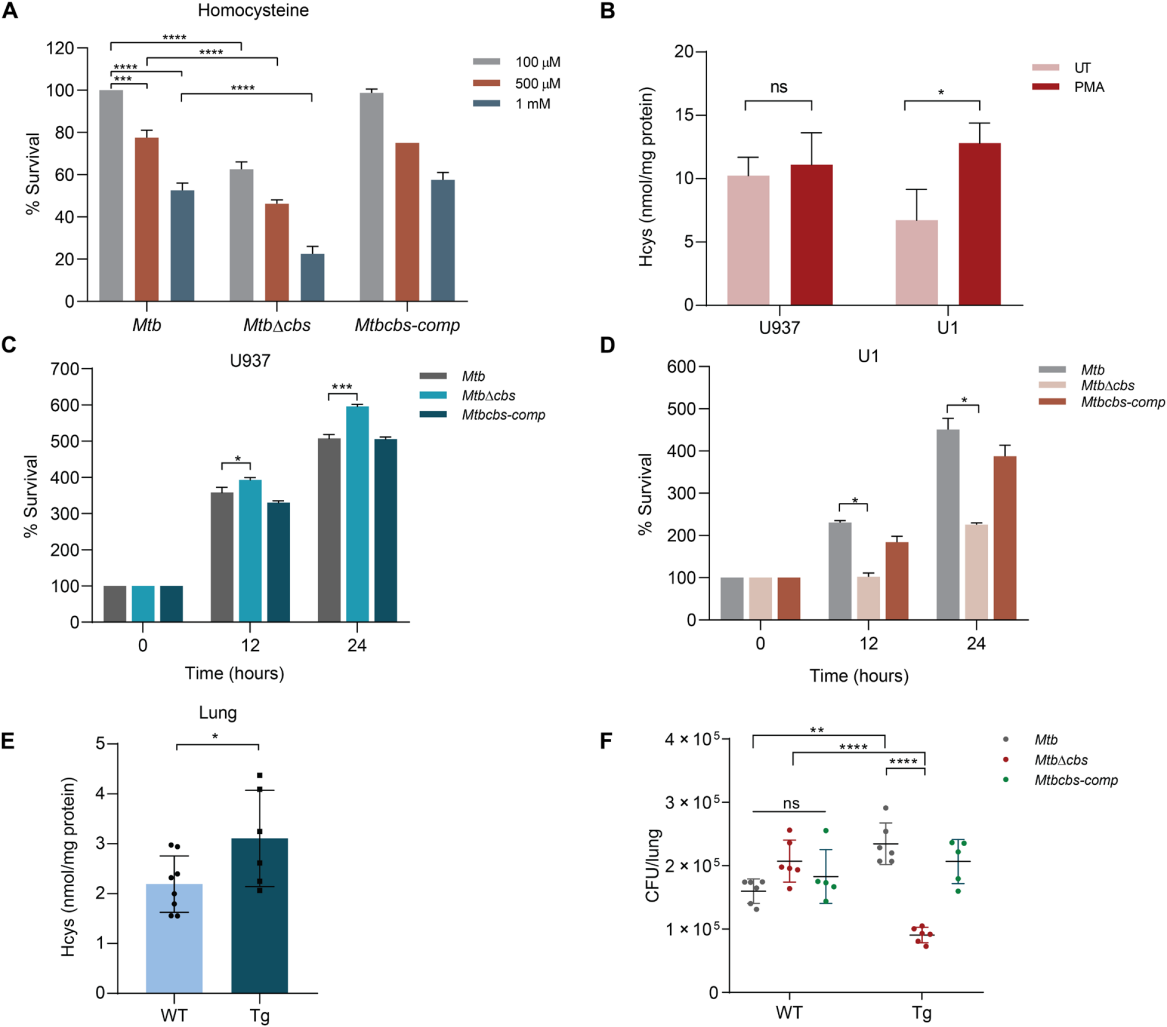
*Mtb*Cbs is crucial for survival under conditions of Hcys excess. (**A**) Exponentially growing *Mtb*, *Mtb*Δ*cbs*, and *Mtbcbs-comp* were treated with the indicated amounts of Hcys for 24 hours, and percent survival was calculated by CFU enumeration. Data represent two independent biological experiments. ***P = 0.0004 and ****P < 0.0001 by two-way ANOVA with Tukey’s multiple comparison test. (**B**) Hcys was measured in PMA activated U937 and U1 cells. Data represent three independent biological experiments. *P = 0.0221 by unpaired *t* test. Survival of *Mtb*, *Mtb*Δ*cbs*, and *Mtbcbs-comp* in PMA-activated (**C**) U937 and (**D**) U1 cell lines. Data represent two independent biological experiments. *P = 0.0137 by unpaired *t* test; ***P = 0.0002. (**E**) Estimation of Hcys in *Salmonella* lipopolysaccharide-treated WT (*n* = 8) and HIV-Tg (*n* = 6) mice. *P = 0.0451 by unpaired *t* test. (**F**) Survival of *Mtb*, *Mtb*Δ*cbs*, and *Mtbcbs-comp* in WT and HIV-Tg mice. WT (*n* = 6 for *Mtb*, *Mtb*Δ*cbs* each and *n* = 5 for *Mtbcbs-comp*) and HIV-Tg (*n* = 6 for *Mtb*, *Mtb*Δ*cbs* each and *n* = 5 for *Mtbcbs-comp*) were infected with 100 CFUs of each strain via the aerosol route. **P = 0.0024 and ****P < 0.0001 by two-way ANOVA with Tukey’s multiple comparison test.

Having shown the importance of SAM in regulating *Mtb*Cbs functionality, we next examined the metabolic consequences of complete loss of CBS activity in *Mtb*. Loss of *Mtb*Cbs did not lead to Cys auxotrophy, indicating that the RTS pathway is a minor Cys biosynthetic pathway in *Mtb* (fig. S23). It is widely known that defects in hCBS leads to excess Hcys, which adversely affects cellular health by inducing oxidative stress, leaching of essential metals (e.g., copper), dysregulation of protein activity (*N*-homocysteinylation), and accumulation of its precursor, SAH (*45*). On this basis, we first investigated the effect of *Mtb*Cbs deletion on AMC metabolites as described for WT *Mtb*. While we were not able to detect Hcys, *Mtb*Δ*cbs* displayed increased abundance of AMC intermediates Met (*P* = 0.0005) and SAH (*P* = 0.0005) and decreased SAM/SAH ratio (*P* = 0.0009) (fig. S24, A to C). This suggests that in the absence of *Mtb*Cbs, *Mtb* likely mitigates potential Hcys accumulation by increasing the biogenesis of AMC intermediates. We next investigated the response of *Mtb*Δ*cbs* to excess Hcys and found that the survival of *Mtb*Δ*cbs* was significantly lower than WT *Mtb* upon exogenous addition of Hcys (Fig. 7A). These results indicate that *Mtb* predominantly requires Cbs to maintain bacterial viability in response to Hcys overload. *Mtb*Δ*cbs* did not exhibit any phenotype in response to various stress conditions including ROS, reactive nitrogen species (RNS), acidic pH (fig. S25, A to D) and antibiotics in vitro (table S5).

Higher circulating Hcys levels are uniformly associated with HIV-infected patients (*46*). We reasoned that *Mtb*Cbs could contribute to survival of *Mtb* during HIV-TB coinfection. We examined this idea by monitoring the survival of *Mtb*Δ*cbs* in U1 monocytic cell line model of HIV-TB coinfection (*20*) and in the lungs of HIV–transgenic (HIV-Tg) mice. U1 cells are generated from U937 monocytes wherein two copies of HIV-1 genome are inserted, and viral multiplication can be easily induced by phorbol 12-myristate 13-acetate (PMA) (*47*). We first confirmed that the treatment with PMA (5 ng/ml) led to an increase in Hcys level in U1 but not in U937 cells (Fig. 7B). We subsequently infected PMA-treated U1 and U937 (uninfected HIV-1 control) with WT *Mtb*, *Mtb*Δ*cbs*, and *Mtbcbs-comp* and measured survival over time, as described previously (*20*). In U937 cells, *Mtb*Δ*cbs* showed marginally greater survival than WT *Mtb* and *Mtbcbs-comp* in U1 cells at 12 and 24 hours postinfection (p.i.). In contrast, survival of *Mtb*Δ*cbs* was ~2.5-fold lower *(P* = 0.0002) in comparison to WT *Mtb* and the *Mtbcbs-comp* at 12 and 24 hours p.i. (Fig. 7, C and D). Last, we exploited the HIV-Tg mouse model (NL4-3Δ gag/pol), which expresses HIV-1 accessory proteins and recapitulates metabolic, redox, and pathological complications (e.g., cardiomyopathy, congenital cataracts, nephropathy, skin lesions, and severe wasting) induced by the virus in humans (*48*). Littermate mice of C57/BL6J strain were used as nontransgenic mice in this study. Similar to PMA-treated U1 cells, higher levels of Hcys levels were detected in the lungs of the (HIV-Tg) mice but not the WT mice (*P* = 0.0451; Fig. 7E). Next, we implanted ~100 bacilli of WT *Mtb*, *Mtb*Δ*cbs*, and *Mtbcbs-comp* in the lungs via the aerosol route and measured survival at 4 weeks p.i. The survival of *Mtb*Δ*cbs* was similar to WT *Mtb* and *Mtbcbs-comp* in the lungs of C57/BL6J strain. In contrast, the lung bacillary burden of *Mtb*Δ*cbs* showed two- to threefold lower (*P* < 0.0001) survival in the HIV-Tg mice as compared to WT *Mtb* and *Mtbcbs-comp* (Fig. 7F). These results suggest that *Mtb*Cbs is important to promote *Mtb* survival in response to Hcys excess encountered by *Mtb* during HIV-TB coinfection.

## DISCUSSION

Studies have shown that de novo Cys and Met biosynthetic pathways are indispensable for *Mtb*’s survival and pathogenesis. In addition, *Mtb* was also reported to possess a poorly characterized RTS pathway, which represents the sole source of Cys and the antioxidant glutathione in many eukaryotes. Here, we report that the first enzyme of the RTS pathway of *Mtb*, i.e., *Mtb*Cbs, is allosterically activated and stabilized by SAM and regulates Met metabolism in *Mtb*. Our study exemplifies how *Mtb* exploits the *Mtb*Cbs-SAM axis as a metabolic sensor to rapidly respond to perturbations of Met metabolism. Furthermore, *Mtb*Cbs ensures bacterial survival against the toxic effects of excess Hcys encountered in vitro, inside macrophages, and in mice infected with HIV.

*Mtb*Cbs displays remarkable similarity to hCBS in terms of enzyme kinetics and SAM-dependent allosteric regulation but resembles its prokaryotic counterparts in being heme-less. Because heme-moiety of hCBS is exceptionally sensitive to ROS and RNS (*49*), the presence of a heme-less CBS in *Mtb* suggests an elegant evolutionary strategy to maintain RTS functionality despite the oxidatively and nitrosatively hostile environment it faces inside the phagocytes (*50*). In contrast to prokaryotic CBS and akin to hCBS, *Mtb*Cbs displays tetrameric assembly and SAM-dependent oligomerization.

*Mtb*Cbs demonstrates significant substrate promiscuity and in addition to its primary product Cysth also yields Lnth and H_2_S. While the calculated kinetic parameters shed light on the preference of substrate utilization, how these parallel reactions are regulated and the exact cellular conditions in which they become physiologically relevant remains unclear. Furthermore, *Mycobacterium smegmatis* mutants defective in meso–diamino pimelate biosynthesis were found to incorporate Lnth into the peptidoglycan (PG), which affected β-lactam susceptibility. Future experiments are needed to understand the contribution of *Mtb*Cbs in regulating the composition of *Mtb’s* PG and response to cell wall–targeting stresses (*51*).

Because the intrinsic instability of the tetrameric hCBS, its full-length structure remained uncharacterized. Although models of the structural mechanism of SAM-mediated allosteric activation of CBS exist and have yielded insightful information, they have relied heavily on the mutants of the full-length enzyme. The structures of hCBS and yeast CBS could only be resolved in the stable mutant dimeric forms of the native tetrameric enzymes [Protein Data Bank (PDB): 4COO, 4L3V, 1JBQ, 4PCU, and 6C4P] (*35*–*38*, *52*). In this study, we resolved the full-length structure of *Mtb*Cbs in its native, activated, and substrate-bound conformations. We propose that the oligomerization of *Mtb*Cbs requires initial association of the monomers via their core domains to create dimers, which subsequently associate via their respective Bateman modules to yield the tetrameric assembly. The 3D structure indicates that the dimerization and tetramerization interfaces are stabilized predominantly by hydrophobic interactions.

The 3D structure of SAM-treated *Mtb*Cbs enabled us to understand the mechanism of SAM-dependent allosteric activation. Previous reports on hCBS showed that in the absence of SAM, the Bateman module blocks the access to catalytic site while in the presence of SAM, it shifts markedly to expose the catalytic site leading to allosteric activation (*36*). In contrast, native *Mtb*Cbs tetramer did not show any changes in the Bateman module in the presence or absence of SAM. We speculate that the likely reason behind the free movement of Bateman module in the dimeric hCBS is its considerable flexibility, which permits greater freedom of movement of the Bateman modules as compared to the more rigid tetrameric *Mtb*Cbs. Our data demonstrate that SAM binding was accompanied by rearrangement of amino acids leading to redistribution of the surface charges in the Bateman module. Furthermore, the active site lysine (K44), which remained buried inside the catalytic core, became more exposed because of the flexible loop’s displacement at the substrate channel, which increased its accessibility. These structural rearrangements upon SAM binding led to enhanced influx of the substrate molecules resulting in enzyme activation. We identified the key amino acid residues responsible for SAM interaction at the Bateman module and confirmed their essentiality in activating *Mtb*Cbs by site directed mutagenesis. Furthermore, we were able to capture the formation of the external aldimine/aminoacrylate in presence of the substrate Ser at the enzyme’s active site. However, our study cannot distinguish between these two intermediates (external aldimine and aminoacrylate) from this resolution structure.

In addition to the structural changes, our data suggest that SAM binding also stabilizes *Mtb*Cbs. We found that depletion of cellular SAM destabilizes *Mtb*Cbs by promoting its pupylation and subsequent degradation by the proteasomal machinery. Pupylation appears to be a protective mycobacterial response upon depletion of AMC metabolites as *Mtb* mutants defective in it were specifically sensitive to folate inhibitors which affect Met metabolism (*53*). Consistent with this, we observed reduced survival of the pupylation resistant *MtbcbsK428* as compared to WT *Mtb*, *Mtb*Δ*cbs*, and *Mtbcbs-comp* in response to PAS and SMX.

Under normal growing SAM-sufficient conditions, deletion of *Mtb*Cbs led to significant depletion of Cysth, indicating that it functions as the housekeeping Cysth synthase in *Mtb* (fig. S26). Under SAM-deficient conditions, destabilization of *Mtb*Cbs leads to repression of the RTS pathway and allows MetB to function as a CGS via the FTS pathway to replenish the AMC. However, the FTS pathway is not the canonical Met biosynthetic pathway in *Mtb* as deletion of MetB does not lead to Met auxotrophy (fig. S27). Consistent with this, the de novo MetA-dependent pathway appears to be the major route to generate AMC intermediates (*7*).

A distinctive feature of *Mtb*Cbs-SAM regulatory node is its exclusively metabolic and posttranslational nature, which bypasses the need for extensive transcription rewiring. The induction of the FTS pathway affords two advantages—first, because it is metabolically regulated, it can respond faster to a sudden drop in SAM. Second, the rapid conversion of *O*-acetylhomoserine to Cysth and not Hcys ensures a unidirectional flow toward Met biosynthesis without interference from any residual activity of *Mtb*Cbs. MetC, an enzyme of the de novo biosynthetic pathway is predicted to be under the control of a SAM-dependent riboswitch (*54*). The expression of *metC* increases in Met starvation (*7*). However, derepression of *metC* and its subsequent increased translation, to lastly up-regulate Met biosynthesis, would constitute a multistep process as opposed to the simple metabolic switching exhibited by MetB. Our study does not preclude the importance of the MetC-dependent methionine biosynthesis, and it is likely that the SAM-riboswitch up-regulation of MetC may take over from the ad hoc FTS pathway under conditions of extended nutritional deprivation such as those encountered in granulomas. However, in the Wayne model of gradual oxygen depletion, *Mtb*Cbs is up-regulated under the microaerophilic conditions [nonreplicative stage 1 (NRP1)] stage, whereas Rv2294 a putative CBL involved in the FTS pathway is induced in hypoxia (NRP2) stage (*55*). It is therefore possible that the RTS to FTS switching might occur as an adaptive measure when *Mtb* transits into dormancy in response to a gradient of oxygen depletion.

Our metabolite data also revealed the failure of AZA to deplete SAM in *Mtb*Δ*cbs* (fig. S28) to the extent observed in WT and complemented strains. The SAM/SAH ratio, an indicator of cellular methyl potential, was lower in *Mtb*Δ*cbs* as compared to WT due to increased SAH abundance in *Mtb*Δ*cbs*. SAH is a potent inhibitor of methyltransferases (*56*). It is possible that inhibition of SAM-dependent methyltransferases by SAH would have decreased the demand for SAM in *Mtb*Δ*cbs* and consequently upon AZA treatment, the overall cellular SAM abundance remained unchanged.

In addition to Cys biosynthesis, CBS is the principal regulator of Hcys metabolism in mammals and prevents its cytotoxic accumulation (*57*). In *M. smegmatis*, exogenous Hcys has been found to disrupt biofilm formation (*58*). Our data indicate that while steady-state Hcys levels were not perturbed upon *Mtb*Cbs deletion, it is crucial for metabolizing surplus Hcys. Hcys acquisition using L-cystine uptake protein transporter (TcyP) has been found to support *Staphylococcus aureus* survival in murine heart and liver (*59*). Using a cell line and transgenic animal pathophysiological model of Hcys excess, i.e., HIV-TB coinfection, we highlighted the importance of *Mtb*Cbs in bacterial survival under this condition. This metabolic cross-talk between the transsulfuration pathways of the host and the pathogen adds another layer of complexity in the pathophysiology and prognosis of TB disease.

## MATERIALS AND METHODS

All chemicals were of analytical grade and purchased from Sigma-Aldrich (St. Louis, MO, USA), unless otherwise specified. All enzymes were purchased from New England Biolabs (Ipswich, MA, USA).

### Bacterial strains and growth conditions

All *Mtb* strains were cultured in BBL Middlebrook 7H9 broth or BBL Middlebrook 7H11 agar (Becton, Dickinson and Company, Franklin Lakes, NJ, USA) at 37°C, supplemented with 10% ADS (albumin, dextrose, and sodium chloride), or 10% OADC (oleic acid, albumin, dextrose, sodium chloride, and catalase). *E. coli* DH5α and BL21 (DE3) were cultured in Luria Bertani broth or agar (HiMedia Laboratories, Mumbai, India). Kanamycin was used at a final concentration of 50 and 25 μg/ml for *E. coli* and *Mtb*, respectively. Hygromycin was used at a final concentration of 150 and 50 μg/ml for *E. coli* and *Mtb*, respectively.

### Purification of recombinant WT *Mtb*Cbs and mutant proteins

*cbs* (Rv1077) was amplified using Q5 High Fidelity polymerase from the genomic DNA of *Mtb* H37Rv using specific forward and reverse primers appended with Nco I and Hind III restriction sites, respectively, and cloned into pET28a expression vector (Novagen) for generating a C-terminal 6× His-tagged construct. For protein purification, cells were grown till an optical density at 600 nm (OD_600_) of 0.6 and induced with 0.5 mM isopropyl-β-d-thiogalactopyranoside at 18°C for 24 hours. The cells were subsequently harvested by centrifugation at 5000 rpm for 10 min at 4°C. The bacterial pellets were resuspended in lysis buffer [50 mM tris-HCl (pH 8.0), 150 mM NaCl, 5% glycerol, 10 mM imidazole, 5 mM β-mercaptoethanol, 50 μM PLP, and 2 mM phenylmethylsulfonyl fluoride] and lysed by sonicating on ice. The lysate was clarified by centrifugation at 13,000 rpm for 45 min at 4°C. Equilibrated Ni–nitrilotriacetic acid (NTA) beads (QIAGEN, Hilden, Germany) were added to the supernatant and kept for binding for 3 hours on a rotating platform at 4°C. The beads were then loaded onto a polypropylene gravity column (QIAGEN, Hilden, Germany) and washed with five-column volumes of equilibration buffer containing 50 mM tris-HCl (pH 8), 300 mM NaCl, 20 mM imidazole, 5% glycerol, and 0.5 mM β-mercaptoethanol. Protein was eluted using increasing concentration of imidazole. The elutes were pooled and dialyzed against two 1.5 liters of buffer volumes, containing 100 mM tris-HCl (pH 8), 150 mM NaCl, 20 μM PLP, 5% glycerol, and 0.5 mM β-mercaptoethanol. They were further concentrated using solid polyethylene glycol 20,000, aliquoted, flash-frozen, and stored at −80°C.

For negative staining and cryo-EM structural study, the Ni-NTA purified protein was loaded to Superdex 200 Increase 10/300 GL column (GE Healthcare Life Sciences, Piscataway, NJ, USA) for SEC [equilibrated with 50 mM tris (pH 8.0) and 150 mM NaCl], and the fractions were collected at a rate of 0.3 ml/min. The same protocol was followed for *Mtb*Cbs protein (~25 μM) incubated with 1 mM SAM (Sigma-Aldrich) and together 1 mM SAM (Sigma-Aldrich) and 10 mM l-serine (Sigma-Aldrich). Standard curve for calculation of molecular weight was made using protein standards of known molecular weight (Bio-Rad gel filtration standard).

### Size exclusion chromatography–multiangle light scattering

The oligomeric state of *Mtb*Cbs protein was determined by SEC-MALS. Suprose-6 increase 10/300 GL (GE Healthcare Life Sciences, Piscataway, NJ, USA) analytical gel filtration column equilibrated with 50 mM tris (pH 8.0) (HiMedia Laboratories, Mumbai, India), 150 mM NaCl (Sisco Research Laboratories, India) with in-line UV (Shimadzu), MALS (mini-DAWN TREOS, Wyatt Technology Corp.) and refractive index detectors (WATERS24614). A total of 0.5 mg/ml (100 μl) of protein was injected. UV, MALS, and refractive index data were collected and analyzed using ASTRA software (Wyatt Technology, Santa Barbara, CA, USA).

### Detection of Cysth and Lnth

Cysth production from serine and Hcys in presence and absence of SAM was detected colorimetrically using the acid-ninhydrin method as described in (*60*). The reaction mixture contained 50 mM tris-HCl buffer (pH 8), 20 μg of purified enzyme, 20 mM serine or *O*-acetylserine, and 10 mM l-Hcys in a total volume of 200 μl. After 20 min of incubation at 37°C, the reaction was stopped by adding 50 μl of 100% ice-cold trichloroacetic acid. The precipitated protein was removed by centrifugation at 13,000 rpm for 15 min. A total of 100 μl of supernatant was mixed with 1 ml of acid ninhydrin reagent (1 g of ninhydrin in 100 ml of concentrated acetic acid and ^1^/_3_ volume of phosphoric acid) and boiled for 5 min, followed by immediate cooling on ice for 2 min and subsequently held at room temperature for 30 min for color development. The absorbance was measured at 455 nm.

The production of Cysth and Lnth was further confirmed by LC–electrospray ionization (ESI) MS/MS. One milliliter of reaction mixture contained 20 μg of purified *Mtb*Cbs in Hepes buffer (pH 8), 20 mM l-cysteine (for Lnth formation), 20 mM l-cysteine or 20 mM serine, and 10 mM l-Hcys (for Cysth formation). The reaction was carried out for 15 min at 37°C and stopped by adding 50 μl of 100% ice-cold trichloroacetic acid. The precipitated protein was removed by centrifugation at 14,000 rpm for 15 min, and the supernatants were flash-frozen and stored at −80°C till analysis. Aliquots were injected into an Dionex 3000 Ultimate LC (Sunnyvale, CA, USA) equipped with C_18_ column using gradients of mobile phases A (0.1% formic acid in water) and B (0.1% formic acid in acetonitrile). The flow rate maintained at 1 ml/min. A 30-min prerun was done for each sample with initial gradient conditions was done to equilibrate the column. Bruker Impact HD QTOF (high resolution hybrid quadrupole-time-of-flight) mass spectrometer (Billerica, MA, USA) equipped with an ESI source (negative ionization) was used in this LC-MS experiment. Data were analyzed using Bruker DataAnalysis (version 4.1 build 362.7). The identities of the thioether species were verified by MS/MS data using the online software CFM-ID 3.0 (Competitive Fragmentation Modeling for Metabolite Identification) (https://cfmid.wishartlab.com/).

### Site-directed mutagenesis

Single primer method of site-directed mutagenesis was used to create point mutants of *Mtb*Cbs. pET28a-*cbs* was amplified in two separate reactions carrying with either the forward or reverse mutagenic primer. Briefly, after the initial denaturation step at 94°C for 2 min, polymerase chain reaction (PCR) was conducted for 30 cycles with denaturation at 94°C for 40 s, primer annealing at 55°C for 40 s, and DNA synthesis at 72°C for 7 min, followed by final extension at 72°C for 10 min. The reaction products were then mixed, heated to 95°C, and cooled slowly to room temperature, to promote annealing and subsequently digested with Dpn I. The digested products were transformed into *E. coli* DH5α ultracompetent cells. Plasmids were isolated from single colonies using the QIAprep Spin Miniprep Kit (QIAGEN, Hilden, Germany) and sequenced for identification of positive mutants.

### Generation of *Mtb*Δ*cbs* knockout and complemented strains

One-kilobyte upstream and downstream regions of the genomic locus of *cbs* (Rv1077) were amplified using Q5 High-Fidelity polymerase and cloned at 5′ and 3′ ends, respectively, of the *loxP-hyg-gfp-loxP* cassette in the mycobacterial *sacB*-based suicide vector pML523 (a gift from M. Niederweis at the University of Alabama at Birmingham). The complete construct of the flanking sequences and the *hyg-gfp* cassette was subsequently amplified and cloned into the pRSF-duet vector (Clontech Laboratories, Mountain View, CA, USA) digested with Hpa I. The pRSF-*cbs* plasmid was pretreated with UV light and electroporated into WT *Mtb* H37Rv for allelic exchange. Positive clones (Hyg^R^-Kan^S^-GFP^+^) were screened by genomic DNA PCR. Disruption of *cbs* was confirmed by reverse transcription quantitative PCR (RT-qPCR) and Western blotting. To unmark *Mtb*Δ*cbs*, the pCre-ZEO-SacB plasmid (a gift from A. K. Pandey, Translational Health Science and Technology Institute, Haryana, India) was electroporated into *Mtb*Δ*cbs* to allow for the loss of the *loxP-hyg-gfp-loxP* cassette from the genome. The resulting unmarked strains were confirmed by the loss of green fluorescent protein (GFP) fluorescence and antibiotic selection.

For creation of the complemented strain, *cbs* was amplified with its native promoter from *Mtb* genome and cloned in the integrative shuttle plasmid pCV125 (Medimmune, Gaithersberg, MD, USA) and electroporated into the unmarked *Mtb*Δ*cbs* strain. *Mtb*Δ*cbs* was also complemented with the mutagenic variants of WT *cbs*, which were unresponsive to allosteric activation by SAM. For this, the gene carrying the respective deletion or point mutation was cloned into the integrative pMV761 vector under *hsp60* promoter.

### UV-VIS spectroscopy

Absorbance measurements for PLP detection were carried out in a BioMate 3S UV-VIS spectrophotometer (Thermo Fisher Scientific, Waltham, MA, USA) in quartz cuvettes using purified proteins (~1 mg/ml).

### Pyridine hemochromagen assay

Pyridine hemochromagen assay was performed according to (*61*). Briefly, purified *Mtb*Cbs was treated with 0.2 M NaOH, 40% (v/v) pyridine, 500 μM potassium ferricyanide in a 1-ml quartz cuvette, and the spectra were recorded in BioMate 3S UV-VIS spectrophotometer (Thermo Fisher Scientific, Waltham, MA, USA) in quartz cuvettes. Subsequently, 0.5 M sodium dithionite in 0.5 M NaOH was added, and the spectra were recorded again. For clarity, only the reduced spectra were plotted.

### Steady-state enzyme kinetics

The steady kinetic parameters of *Mtb*Cbs were determined by a continuous spectrophotometric lead acetate assay (*32*). Briefly, the reaction mixture comprised of 20 μg of the purified enzyme in Hepes buffer (pH 8), 0.4 mM lead acetate, Cys (0 to 50 mM), Hcys (0 to 8 mM), and 500 μM SAM as indicated. H_2_S production from Cys and Hcys was estimated at 390 nm using ϵ = 5500 M^−1^ for lead sulfide formation. Absorbance measurements were carried out in VersaMax microplate reader (Molecular Devices, San Jose, CA, USA) in a 96-well plate with a reaction volume not exceeding 200 μl. To compute the kinetic parameters, velocity data were fitted to either the standard Michaelis-Menten or bisubstrate kinetics equations.

### Microscale thermophoresis

SAM binding to purified native and mutated *Mtb*Cbs was evaluated using microscale thermophoresis experiment (Nanotemper Technologies, Munich, Germany), that measures binding affinity of SAM. For this assay, the final protein concentration used was 100 nM. The protein was labeled with 100 nM red tris-NTA dye (Nanotemper Technologies, Munich, Germany) in the C terminus of the His-tag. Monolith NT.115 capillaries (Nanotemper Technologies, Munich, Germany) were used in each experiment. In the binding assay, the protein concentration for both native and mutated was kept constant. Both proteins were incubated with 16 twofold serial dilutions of the ligand SAM. The ligand was solubilized in a protein-containing buffer [50 mM Hepes (pH 7.4), 150 mM NaCl, and 3% glycerol]. The starting concentration for the ligand SAM was 500 μM for all the cases.

### Fluorescence thermal shift assay

Fluorescence thermal shift assay was performed using 10 μg of purified protein and SYPRO orange dye (Bio-Rad, Hercules, CA, USA) using the fluorescence resonance energy transfer channel of the CFX96 RT-PCR System (Bio-Rad, Hercules, CA, USA).

### Negative staining sample preparation and visualization by TEM

SEC-purified native *Mtb*Cbs in the presence of SAM, SAM + serine, and the I357A mutant was visualized by negative staining electron microscopy (EM) to analyze homogeneity and particle distribution. All the samples were prepared by conventional negative staining methods. A carbon-coated copper grid (EM grid, 300 mesh; TedPella) was glow-discharged for 30 s at 20 mA. The purified protein was dialyzed without glycerol buffer for negative staining analysis [(50 mM tris (pH 8.0) (HiMedia Laboratories, Mumbai, India) and 150 mM NaCl (Sisco Research Laboratories)]. A total of 3.5 μl of the sample (0.1 mg/ml) was added to the glow-discharged (GloQube glow discharge system, Quorum) carbon-coated copper grid for 30 s. The extra sample was blotted out. Negative staining was performed using 1% uranyl acetate (98% uranyl acetate; ACS Reagent, Polysciences Inc. Warrington, PA, USA) solution for 20 s. The grid was air-dried. The negatively stained sample for native *Mtb*Cbs, SAM-treated *Mtb*Cbs, and I357A mutant were visualized at room temperature using Tecnai T12 electron microscope equipped with a LaB_6_ filament operated at 120 kV, and images were recorded using a side-mounted Olympus VELITA (2000 × 2000) charge-coupled device camera at a magnification of ×220,000 (2.54 Å per pixel). One hundred forty images were collected for native *Mtb*Cbs, 215 images were collected for SAM-treated *Mtb*Cbs, and 55 images were collected for I357A mutant manually for image processing. SAM + serine–treated *Mtb*Cbs was visualized at room temperature using Talos L120C transmission electron microscope (Thermo Fisher Scientific) equipped with Ceta (4000 × 4000) camera. Images were recorded at a magnification of ×92,000 (1.52 Å per pixel). Around 40 images were collected for *Mtb*Cbs treated with SAM + serine.

### Negative staining data processing and calculation of reference-free 2D classification

The evaluation of micrographs was done with EMAN 2.1 (*62*). A total of 12,541 particles for *Mtb*Cbs and 15,120 particles for SAM-treated *Mtb*Cbs were manually picked using EMAN 2.1. The total number of particles were extracted separately for *Mtb*Cbs and *Mtb*Cbs treated with SAM using e2boxer.py in EMAN2.1 software package. Initially, e2refine2d.py was used to perform the 2D reference-free class averaging without any masking to visualize the structural integrity of *Mtb*Cbs. However, same dataset for both the samples (*Mtb*Cbs and SAM-treated *Mtb*Cbs) were subjected to reference-free 2D classification using RELION 2.1. In the first round of 2D classification, *Mtb*Cbs and SAM-treated *Mtb*Cbs dataset were split into 250 and 300 classes, respectively. The classes having the best signal-to-noise ratio were selected for the second round of 2D classification. A total of 8189 particles for *Mtb*Cbs and 10,738 particles for SAM-treated *Mtb*Cbs were selected for the second round of 2D classification. In the second round of 2D classification, *Mtb*Cbs and SAM-treated *Mtb*Cbs dataset were split into 150 and 200 classes, respectively. Last, 69 best classes with 6875 particles and 89 best classes with 9726 particles from *Mtb*Cbs and SAM-treated *Mtb*Cbs were selected for further 2D classification. The cleaned datasets of the samples were used for reference-free 2D classification, and reference-free 2D class averages of different particle projections were calculated using simple_prime2D of SIMPLE 2.1 software (*63*) with a mask diameter of 100 pixels at 2.54 A per pixel.

For SAM + serine–treated *Mtb*Cbs, around 5000 were manually picked using EMAN 2.1. The total number of particles were extracted using e2boxer.py in EMAN2.1 software package. Initially, e2refine2d.py was used to perform the 2D reference-free class averaging without any masking to visualize the structural integrity of substrate-treated CBS protein. However, the same dataset was subjected to reference-free 2D classification using RELION 2.1. Several rounds of 2D classification were performed to clean the dataset. The cleaned dataset of the samples was used for further processing, and reference-free 2D class averages of different particle projections were calculated using simple_prime2D of SIMPLE 2.1 software at 1.52 Å per pixel.

For I357A mutant, around 4530 particles were manually picked using EMAN 2.1 and extracted using e2boxer.py in EMAN 2.1. Several rounds of 2D classification were performed using RELION 2.1 to clean the dataset. The cleanest dataset of samples was used for reference-free 2D classification of different particle projections and conformations using simple_prime2D of SIMPLE 2.1 software at 2.54 Å per pixel.

### Cryo-EM sample preparation

R 1.2/1.3 (QUANTIFOIL) (Electron Microscopy Sciences) 300-mesh copper grids were glow-discharged for 90 s at 20 mA using Quorum GlowCube before sample preparation. The purified protein was dialyzed without glycerol buffer for negative staining analysis [50 mM tris (pH 8.0) (HiMedia Laboratories, Mumbai, India) and 150 mM NaCl (Sisco Research Laboratories)]. Three microliters of freshly prepared *MtbCbs*, *Mtb*Cbs treated with SAM, and *Mtb*Cbs treated with SAM + serine were added to the glow-discharged grids, incubated for 10 s, followed by blotting of 6.5 s at 100% humidity. The sample-containing grids were quickly plunged into liquid ethane using an FEI Vitrobot IV plunger (Thermo Fisher Scientific).

### Cryo-EM data acquisition using 200-kV Talos Arctica and preliminary data processing for native *Mtb*Cbs and SAM-treated *Mtb*Cbs

The cryo-EM data collection was initially performed in 200-kV cryo-TEM (*64*) to achieve our targets and observe the structural integrity of *Mtb*Cbs and *Mtb*Cbs with SAM at cryogenic temperature. Briefly, a small dataset (~300) was collected using 200-kV Talos Arctica cryo-EM (Thermo Fisher Scientific) equipped with K2 direct electron detector (Gatan Inc.) using Latitude-S automatic data acquisition tools. The data acquisition was performed at ×42,200 magnification and a calibrated pixel size of 1.17 Å at specimen level. Total electron dose of about 40 *e*^−^/ Å^2^ at the defocus range of −0.75 to −2.25 μm (table S3). Last, around 10,000 particles were selected for 2D classification, and 2D class averages indicate that *Mtb*Cbs with and without allosteric activators is organized as a tetramer.

### Cryo-EM data acquisition using 300-kV Titan Krios and data processing for native *Mtb*Cbs and SAM-treated *Mtb*Cbs

For high-resolution cryo-EM structural characterization, cryo-EM data acquisition was performed using Thermo Scientific Titan Krios Transmission Electron Microscope operated at 300 kV equipped with a Falcon direct electron detector (Thermo Fisher Scientific). Images were collected automatically using Thermo Scientific EPU software at a pixel size of 1.07 Å at specimen level. Total electron dose was about 30 *e*^−^/Å^2^ at a defocus range of −2.2 to −3.9 μm. Data were recorded in a movie file for a total of 25 frames. A total of 1349 micrographs were collected for *Mtb*Cbs, and 1286 micrographs were collected for *Mtb*Cbs treated with SAM for further data processing (table S3).

Primarily, data processing was performed using RELION 3.0 (*65*). Initially, beam-induced motion correction was performed for each movie file using MotionCorr2 software (*66*). The micrographs having poor signal-to-noise ratio were discarded after screening in cisTEM software package (*67*), and the best micrographs were considered for further data processing. Contrast transfer function (CTF) was estimated using CTFFIND 4.1.13 (*68*). Initially, around 8000 particles were picked manually, and reference-free 2D classification was calculated using RELION 3.0 for both *Mtb*Cbs and *Mtb*Cbs-treated with SAM datasets. Best 2D class averages were selected as template for automated particle picking for both the datasets. After automatically particle picking, 980,992 particles were picked for native *Mtb*Cbs, and 803,322 particles were picked for *Mtb*Cbs treated with SAM. Automatically picked particles were extracted with a box size of 240 pixels, calibrated pixel size of 1.07 Å for both the datasets. After particle sorting, 961,170 particles were selected for native *Mtb*Cbs, and 787,020 particles were selected for SAM-treated *Mtb*Cbs. Several rounds of 2D classification were run subsequently to clean both the datasets. Around 12,000 particles were selected with best signal-to-noise ratio to generate ab initio model. Extracted particles from the best 2D class averages were used to calculate the 3D classification using the previously determined ab initio structure as reference. However, the ab initio model was low-pass–filtered to 30 Å, to use as an initial 3D model for 3D classification.

For native *Mtb*Cbs, after three rounds of 2D classifications, 605,966 particles were selected for 3D classification. 3D classification was performed with C2 symmetry, and 605,966 particles dataset divided in 10 classes. From 3D classification result, class 4 (178,599 particles) was observed as a tetramer and well resolved. 3D autorefinement was carried out for class 4 using soft mask in RELION 3.0. Followed by 3D autorefinement, per-particle defocus refinement with beam tilt correction was done. Corrected particles were subjected for Bayesian polishing. Polished particles were subjected for another round of 3D refinement. A 3D autorefined map of native *Mtb*Cbs was sharpened using RELION 3.0 (table S3).

For SAM-treated *Mtb*Cbs, after three rounds of 2D classifications, 485,258 particles were selected for 3D classification. 3D classification was performed with C2 symmetry, and 485,258 particles were divided in eight classes. From 3D classification, result shows that class 6 (146,444 particles) was observed as a tetramer and well resolved. 3D autorefinement was carried out for class 6 using soft mask in RELION 3.0. The consensus particles set was selected for CTF refinement to correct the per-particle defocus values and beam tilt correction. Corrected particles were subjected for Bayesian polishing. Polished particles were subjected for another round of 3D refinement. 3D autorefined map of SAM-treated *Mtb*Cbs was sharpened using RELION 3.0 (table S3). The structural differences between native *Mtb*Cbs and SAM-treated *Mtb*Cbs were performed after superimposing both the cryo-EM density maps in UCSF Chimera (*69*) and UCSF ChimeraX (*70*, *71*).

### Cryo-EM data acquisition using 200-kV Talos Arctica and data processing for SAM + serine–treated *Mtb*Cbs

The cryo-EM data collection for SAM + serine–treated *Mtb*Cbs was performed using 200-kV Talos Arctica cryo-TEM (Thermo Fisher Scientific) to achieve our targets and observe the structural changes of *Mtb*Cbs in the presence of SAM + serine at cryogenic temperature. A dataset of 1666 micrographs was collected in a movie file of 20 frames using 200-kV Talos Arctica cryo-EM (Thermo Fisher Scientific) equipped with a K2 direct electron detector (Gatan Inc.) using Latitude-S automatic data acquisition tools. The data acquisition was performed at ×42,200 magnification, a calibrated pixel size of 1.17 Å at specimen level, and a total electron dose of about 40 *e*^−^/ Å^2^ at the defocus range of −0.75 to −2.25 μm.

Primarily, data processing was performed using RELION 3.1 (*65*). Initially, beam-induced motion correction was performed for each movie file using MotionCor2 software. The micrographs having a poor signal-to-noise ratio were discarded after screening in cisTEM software package (*67*), and the best micrographs were considered for further data processing. The CTF was estimated using CTFFIND 4.1.13 (*68*). Initially, around 8000 particles were picked manually and reference-free 2D classification was calculated using RELION 3.1. Best 2D class averages were selected as template for automated particle picking. After automatically particle picking, 786,924 particles were picked. Automatically picked particles were extracted with a box size of 240 pixels and calibrated pixel size of 1.17 Å. Several rounds of 2D classification were run subsequently to clean the dataset. Around 12,000 particles were selected with the best signal-to-noise ratio to generate the ab initio model. Extracted particles from the best 2D class averages were used to calculate the 3D classification using the ab initio structure.

For *Mtb*Cbs in the presence of SAM + Ser, after three rounds of 2D classifications, 643,806 particles were selected for 3D classification. The 3D classification was performed with C2 symmetry, and the 643,806 particles dataset was divided into four classes. From the 3D classification result, class 4 (172,994 particles) was observed as a tetramer and well resolved. 3D autorefinement was carried out for class 4 using a soft mask in RELION 3.1. Followed by 3D autorefinement, per-particle defocus refinement was performed with beam tilt correction, anisotropic magnification correction, and per micrograph astigmatism fitting. Corrected particles were subjected for Bayesian polishing. Polished particles were subjected for another round of 3D refinement. 3D autorefined map of *Mtb*Cbs in the presence of substrate was sharpened using RELION 3.1 (table S3). The structural differences after substrate treatment were observed after superimposing both the cryo-EM density maps in UCSF Chimera and UCSF ChimeraX.

### Fourier shell correlation calculation and local resolution estimation

All structures were visualized using UCSF Chimera and UCSF ChimeraX. Fourier shell correlation was estimated for all the maps at 0.143. Local resolution estimation was done for all the maps using unfiltered auto-refined maps with ResMap (*72*).

### Homology modeling

Because of the unavailability of full-length *Mtb*Cbs structure, we performed homology modeling using SWISS-MODEL (*73*) to predict a probable model for *Mtb*Cbs. The homology model generated for catalytic core region and Bateman module separately. The homology model was fitted into the catalytic core region and Bateman module in cryo-EM map of *Mtb*Cbs. All the model fittings were performed into the respective cryo-EM density maps using UCSF Chimera and UCSF ChimeraX.

### Atomic model building and validation

Initially, cryo-EM maps were docked with predicted homology model in catalytic core and Bateman module separately (generated in SWISS-MODEL). The docking was done using phenix:dock_in_map program in PHENIX (*74*). The docked model was refined using phenix:real_space_refine program in PHENIX. The refined model and cryo-EM maps were imported in Coot (*75*) and manually fitted and corrected. The model was subsequently rebuilt manually in the missing area of homology model using Coot. This process was done iteratively, and after each round fitting in Coot, the model was refined iteratively using phenix:real_space_refine program in PHENIX. The final models were analyzed using MolProbity in PHENIX. The models were validated using phenix:validation_tool in PHENIX.

Initially, the PLP was fitted manually at the active site for both the maps using Coot. Later, refinement was carried out for the model with PLP using phenix:real_space_refine program. The SAM density was not visible for SAM-treated *Mtb*Cbs cryo-EM model at an RMSD value of 6σ. SAM was docked in the possible orientation of the protein at an RMSD value of 3σ to understand SAM interaction (table S3).

### Analysis of atomic models

Atomic models and cryo-EM maps were visualized and analyzed using UCSF Chimera, UCSF ChimeraX, and PyMOL (*76*). EMRinger score (*77*) for each map was calculated using PHENIX.

#### *Identification of responsible amino acid residues for SAM interaction*

hCBS Bateman module (PDB: 4PCU) and *Mtb*Cbs Bateman module were superimposed using PyMOL. The predicted amino acids responsible for SAM interaction in *Mtb*Cbs were determined and marked.

#### *Identification of amino acid residues at the dimer and tetramer interface*

Initially, the amino acid residues present at the dimer and tetramer interface was calculated using PDBSum (*78*). The distance between two amino acid residues were calculated in UCSF Chimera and confirmed the selected amino acids for site directed mutagenesis.

#### *Determination of active site pocket for native MtbCbs and SAM-treated MtbCbs*

The active site pocket for both the models was calculated using CASTp 3.0 software (*79*). The pocket was visualized in UCSF Chimera.

### Pulse proteolysis assay

Pulse proteolysis was performed as described in with some modifications (*39*). Purified *Mtb*Cbs (0.5 mg/ml) was equilibrated overnight in a chilled water bath maintained at 4°C. The assay buffer comprised 20 mM tris-HCl (pH 8), urea (1 to 7 M), and 10 mM CaCl_2_. Thermolysin from *Geobacillus stearothermophilus* (0.1 mg/ml) (Sigma-Aldrich, St. Louis, MO, USA) was used for digestion. *Mtb*Cbs was incubated with 500 μM SAM at 37°C for 15 min before urea equilibration to carry out pulse proteolysis in the presence of SAM. Proteolytic pulse was given for exactly 1 min and was subsequently quenched in 20 mM EDTA. Samples were resolved on 12% SDS–polyacrylamide gel electrophoresis (PAGE) gel and visualized by Colloidal Coomassie staining. Band intensities were quantified using ImageJ. *C*_m_ (midpoint of the urea-induced unfolding transition) was determined by plotting *f*_fold_ (band intensity of intact protein at a given concentration of urea divided by band intensity of intact protein in the absence of urea) and fitting it to a sigmoidal model. The global stability of the protein (Δ*G*°_unf_) was calculated by multiplying the *C*_m_ by the *m* value (denaturant dependence of Δ*G*°_unf_). The *m* value of *Mtb*Cbs was estimated as described by multiplying the number of amino acid residues by −0.013.

### RT-qPCR analysis

One microgram of total cellular RNA was purified (*80*) and then treated with Turbo deoxyribonuclease (Invitrogen, Carlsbad, CA, USA). From this, 200 ng of RNA was used for cDNA synthesis using the iScript Select cDNA Synthesis Kit. A 1× iQ SYBR Green Supermix cocktail was used for the RT-qPCR reaction with gene specific primers in the CFX96 RT-PCR System (Bio-Rad, Hercules, CA, USA). For gene expression analysis, 16*S* ribosomal RNA was used as internal normalization control in all cases.

### Cell lysis and Western blotting

*Mtb* cell lysis was performed by four rounds of bead beating (40 s) in FastPrep-24 Classic bead beating grinder (MP Biomedicals, Irvine CA, USA) using glass beads in a total volume of 1 ml of lysis buffer with intermittent chilling on ice. The lysis buffer comprised 50 mM tris-HCl, 150 mM NaCl, 5% glycerol, 0.5 mM β-mercaptoethanol, and 1× cOmplete EDTA-free protease inhibitor cocktail (Roche, Basel, Switzerland). For Western blotting, 50 μg of total cell lysate was resolved on a 12% SDS-PAGE gel and transferred to immunoblot polyvinylidene difluoride membrane in a Mini Trans-blot Cell (Bio-Rad, Hercules, CA, USA) at a constant current of 350 mA. Cbs was detected using Cbs antisera (anti-rabbit; 1:10,000) and anti-rabbit immunoglobulin G horseradish-linked antibody (1:10,000; Cell Signaling Technology, Danvers, MA, USA). Rho (gift from V. Nagaraja, Department of Microbiology and Cell Biology, Indian Institute of Science) was used as internal control (anti-rabbit; 1:10,000). The blots were developed using Clarity Max ECL Western blotting substrate in a GelDoc XR+ Gel Documentation system. Densitometry was performed by plotting band intensities quantified using ImageJ (https://imagej.nih.gov/ij/download.html).

### Metabolite extraction and analysis

Metabolite extraction for the targeted analysis of RTS pathway and AMC intermediates was carried out as outlined in (*81*). Briefly, exponentially growing *Mtb* cultures (OD_600_ = 0.6) were treated with AZA for the indicated time period or left untreated. The cells were quenched with four volumes of 60% methanol, maintained at −45°C for 5 min in a dry ice–methanol bath, and centrifuged at 5000 rpm for 5 min. The pellet was resuspended in 750 μl of 60% methanol, maintained at −45°C, and centrifuged at 5000 rpm for 5 min. Subsequently, the pellet was resuspended in 1 ml of 75% ethanol, incubated at 80°C for 3 min exactly, and kept on ice immediately for 5 min, followed by centrifugation at 13,000 rpm for 15 min. The final supernatant was lyophilized and stored at −80°C till further analysis.

### Survival assays

Exponentially growing cultures were diluted in 7H9-ADS to an OD_600_ of 0.1 and treated with the indicated concentrations of drugs for 24 hours at 37°C with constant shaking. Cells were plated on 7H11-ADS plates and incubated at 37°C for 3 weeks. Susceptibility was expressed as percentage survival of the strains in comparison to untreated controls.

### Animal experimentation

WT and heterozygous HIV-Tg mice Tg26 (C57BL/6 background) containing HIV proviral DNA with mutated 3-kb region of *gag* and *pol* genes were used. HIV-Tg26 was backcrossed with C57BL/6 for eight generations to create a more stable HIV-Tg26 (FVB/N) line by R. L. Sutliffe (Veterans Affairs Medical Center, Emory University, Atlanta, GA). The breeding colony was maintained in the BSL3 Animal Facility at CIDR, IISc, according to the Institutional Animal Ethical Committee (IBSC No. IBSC/IISc/AS/25/2019) guidelines. Genotyping was performed at 4 weeks of age by tail vein PCR. Six- to eight-week-old transgenic mice were used for the experiment. WT littermates were used as controls.

For the chronic model of infection, mice aged 6 to 8 weeks were infected by the aerosol route with 100 bacilli using a Madison chamber aerosol generation chamber and housed for 4 weeks for infection progression. At the indicated time points, mice were euthanized, and the lungs and spleen were harvested for analysis of bacterial burden and histopathology. Lung and spleen samples were homogenized in sterile 2 ml of phosphate-buffered saline (PBS), serially diluted, and plated on 7H11-OADC plates containing BBL MGIT PANTA antibiotic cocktail (Becton, Dickinson and Company, Franklin Lakes, NJ, USA) for colony-forming unit (CFU) enumeration.

### Homocysteine quantification

Homocysteine was quantified in cell lines and animal lungs using the Euro Diagnostics homocysteine assay kit (Chennai, India) according to the manufacturer’s protocol.

### Ethics statement

This study was carried out in strict accordance with the guidelines provided by the Committee for the Purpose of Control and Supervision on Experiments on Animals (CPCSEA), Government of India. The protocol of animal experiment was approved by animal ethical committee on the Ethics of Animal Experiments, IISc, Bangalore, India (approval number: CAF/Ethics/544/2017). All humane efforts were made to minimize the suffering.

### Detection of ROS production by *Mtb*

ROS generation was detected using the fluorescent dye CellROX Deep Red reagent (Invitrogen, Carlsbad, CA, USA). Briefly, CellROX Deep Red was added to 500 μl of treated or untreated samples at a final concentration of 2.5 μM and incubated at 37°C with constant shaking. Subsequently, cells were pelleted and washed twice with 1× PBS and resuspended in 150 μl of PBS, and in all cases 10,000 events were analyzed with flow cytometry with λ_Ex_ = 644 nm and λ_Em_ = 665 nm in BD FACSAria flow cytometer (BD Biosciences, San Jose, CA, USA). Fold change in median fluorescence intensities with respect to untreated controls were plotted.

### Statistical analysis

All statistical analyses were performed using GraphPad Prism version 8.0 (GraphPad Software, San Diego, CA, USA). All data indicated are means ± SD. Data were first tested for normality by Shapiro-Wilk’s test, followed with requisite parametric statistical test.

### Data fitting

All custom data fittings were obtained by nonlinear regression using OriginPro 2021 (OriginLab, Northampton, MA, USA).

## Notes

View/request a protocol for this paper from *Bio-protocol*.
