# Supplementary material for: S-Adenosylmethionine–responsive cystathionine β-synthase modulates sulfur metabolism and redox balance in Mycobacterium tuberculosis
Source: Sci Adv. 2022 Jun 24;8(25):eabo0097. doi: 10.1126/sciadv.abo0097 (PMC9232105; doi:10.1126/sciadv.abo0097)
Supplement: Supplementary file 1 — Supplementary Materials Figs. S1 to S28 Tables S1 to S5 [file sciadv.abo0097_sm.pdf]

Science Advances
AAAS

Supplementary Materials for

S-Adenosylmethionine–responsive cystathionine β-synthase modulates sulfur metabolism and redox balance in *Mycobacterium tuberculosis*

Parijat Bandyopadhyay *et al.*

Corresponding author: Amit Singh, asingh@iisc.ac.in; Somnath Dutta, somnath@iisc.ac.in

Sci. Adv. **8**, eabo0097 (2022)
DOI: 10.1126/sciadv.abo0097

This PDF file includes:

Supplementary Materials
Figs. S1 to S28
Tables S1 to S5

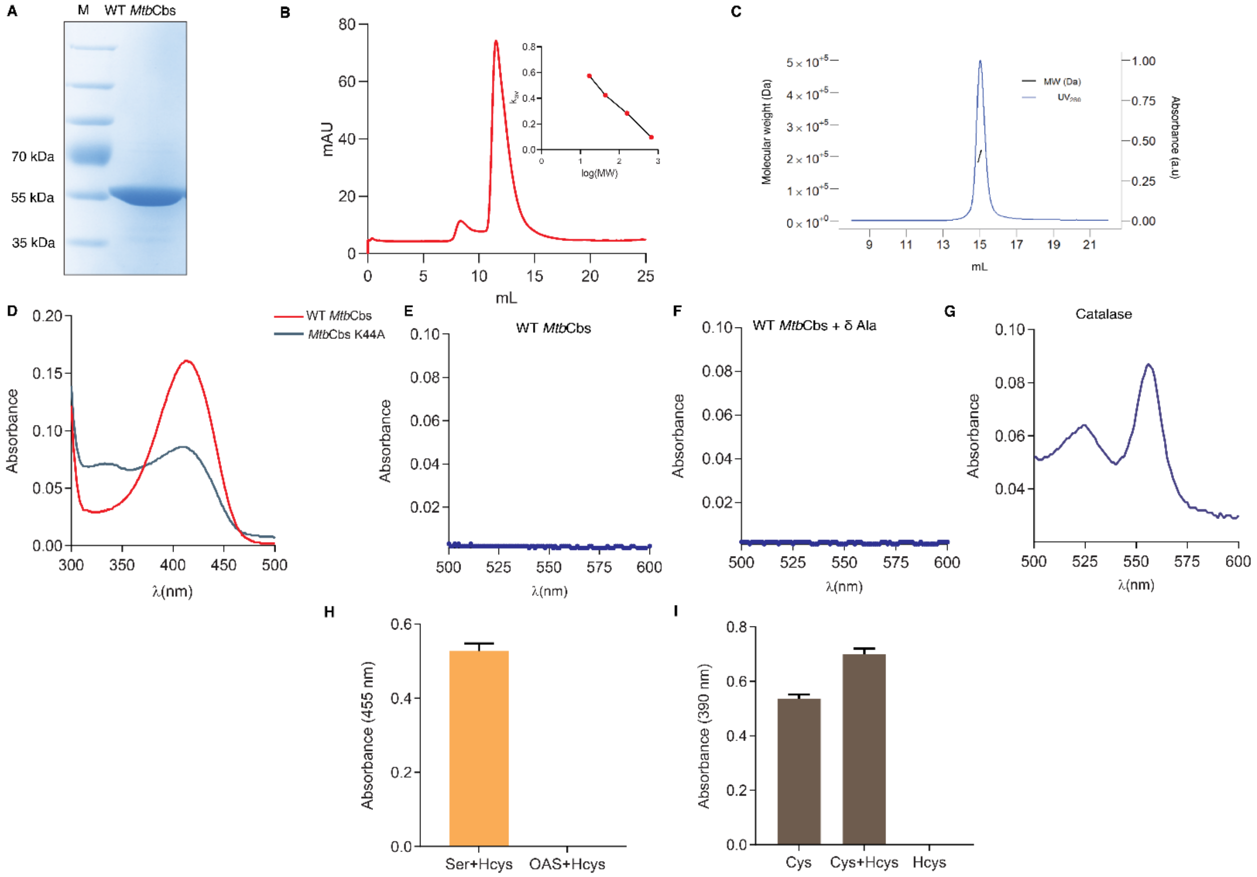

Figure S1: Purification and preliminary biophysical analysis of *Mtb*Cbs.

(A) WT *Mtb*Cbs was purified using Ni-NTA affinity chromatography with a molecular weight of ~55 kDa. **(B)** Size exclusion chromatography (SEC) profile of WT *Mtb*Cbs. Calibration curve (inset) was plotted using the gel phase distribution coefficient k_{av} against proteins of known molecular weight and **(C)** SEC-MALS profile for *Mtb*Cbs indicating the molecular weight (203 kDa, 1% error rate). **(D)** UV-VIS spectra of WT *Mtb*Cbs. Protonated PLP-aldimine absorbs strongly at 420 nm which is diminished in the K44A mutant. Pyridine hemochromagen assay to determine the presence of heme in the purified proteins. **(E)** WT *Mtb*Cbs purified from culture grown in normal and **(F)** media enriched with δ-ALA did not show the characteristic reduced hemochromagen spectra as shown by heme containing proteins. **(G)** Catalase was used as positive control. **(H)** Acid-

ninhydrin assay for the detection of Cysth. Cysth was detected at OD_{455} nm when Ser (20 mM) was used with Hcys (10 mM) but not when O-acetylserine was used. Data represent mean ± SD of two independent biological replicates. **(I)** Lead acetate assay for the detection of H_2S. H_2S was detected at OD 390 nm when Cys (20 mM) was used with Hcys (10 mM) but not when Hcys was used alone. Data represent mean ± SD of two independent biological replicates.

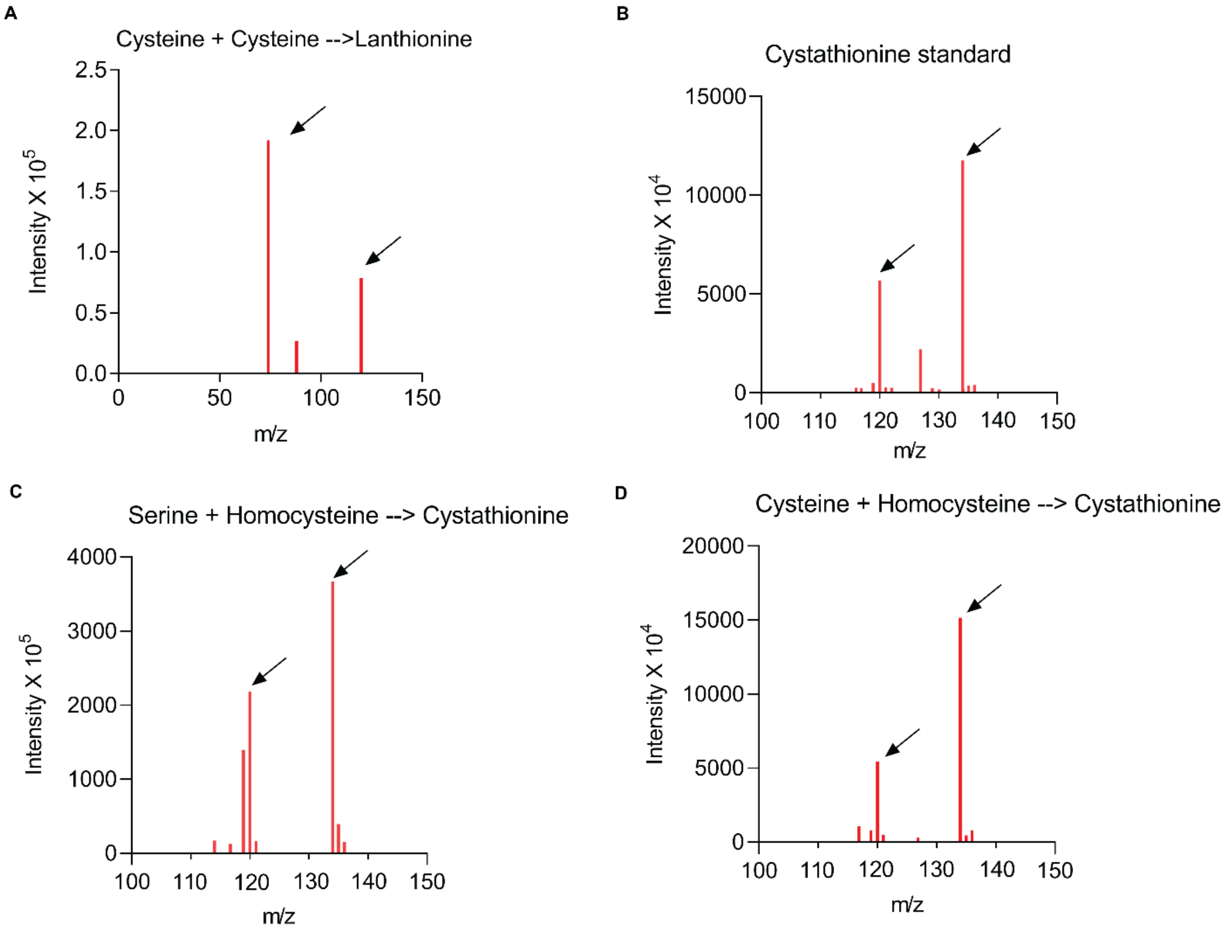

Figure S2: LC-MS/MS analysis of *Mtb*Cbs reaction products.

(A) LC-MS/MS analysis of the reaction products when Cys alone was used as the substrate. The m/z ratios obtained corresponded to lanthionine as the parent product using the CFM-ID database as reference. **(B)** LC-MS/MS analysis of Cysth standard. **(C)** LC-MS/MS analysis of the reaction products when Ser+Hcys and **(D)** Cys+Hcys were used as substrates. The m/z ratios obtained corresponded to cystathionine as the parent product using the CFM-ID database as reference.

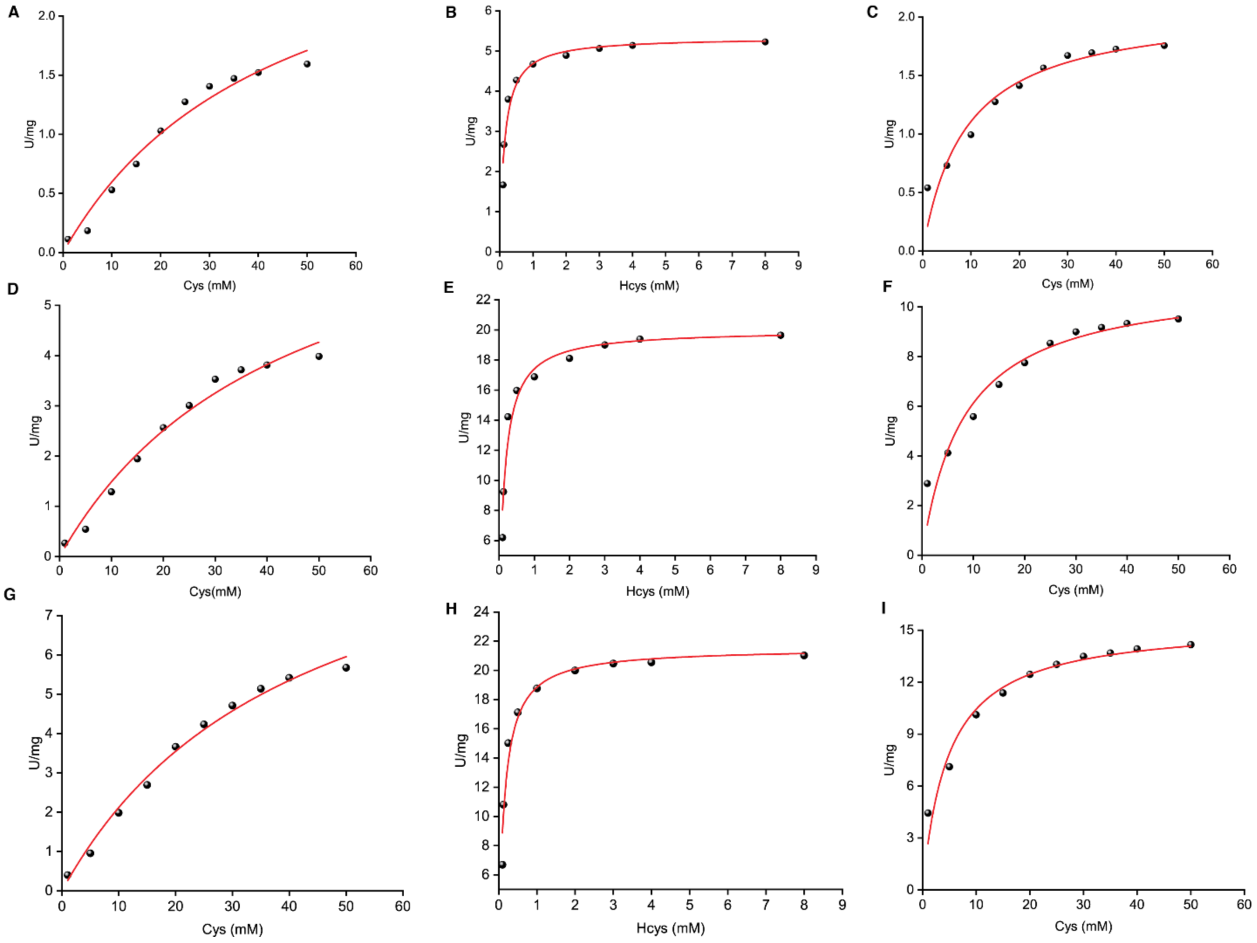

Figure S3: Steady state kinetics of *Mtb*Cbs and *Mtb*Cbs$_{1\text{-}317}$

Steady state kinetics of *Mtb*Cbs showing the dependence of the reaction on **(A)** Cys **(B)** the bisubstrate reaction on Hcys (Cys held constant at 20 mM) and **(C)** bisubstrate reaction on Cys (Hcys held constant at 1 mM). Steady state kinetics of *Mtb*Cbs showing the dependence of the reaction on **(D)** Cys **(E)** the bisubstrate reaction on Hcys (Cys held constant at 20 mM) and **(F)** bisubstrate reaction on Cys (Hcys held constant at 1 mM) in presence of 500 μm SAM. Steady state kinetics of *Mtb*Cbs$_{1\text{-}317}$ showing the dependence of the reaction on **(G)** Cys **(H)** the bisubstrate reaction on Hcys (Cys held constant at 20 mM) and **(I)** bisubstrate reaction on Cys (Hcys held constant at 1 mM). Representative data of one biological replicate.

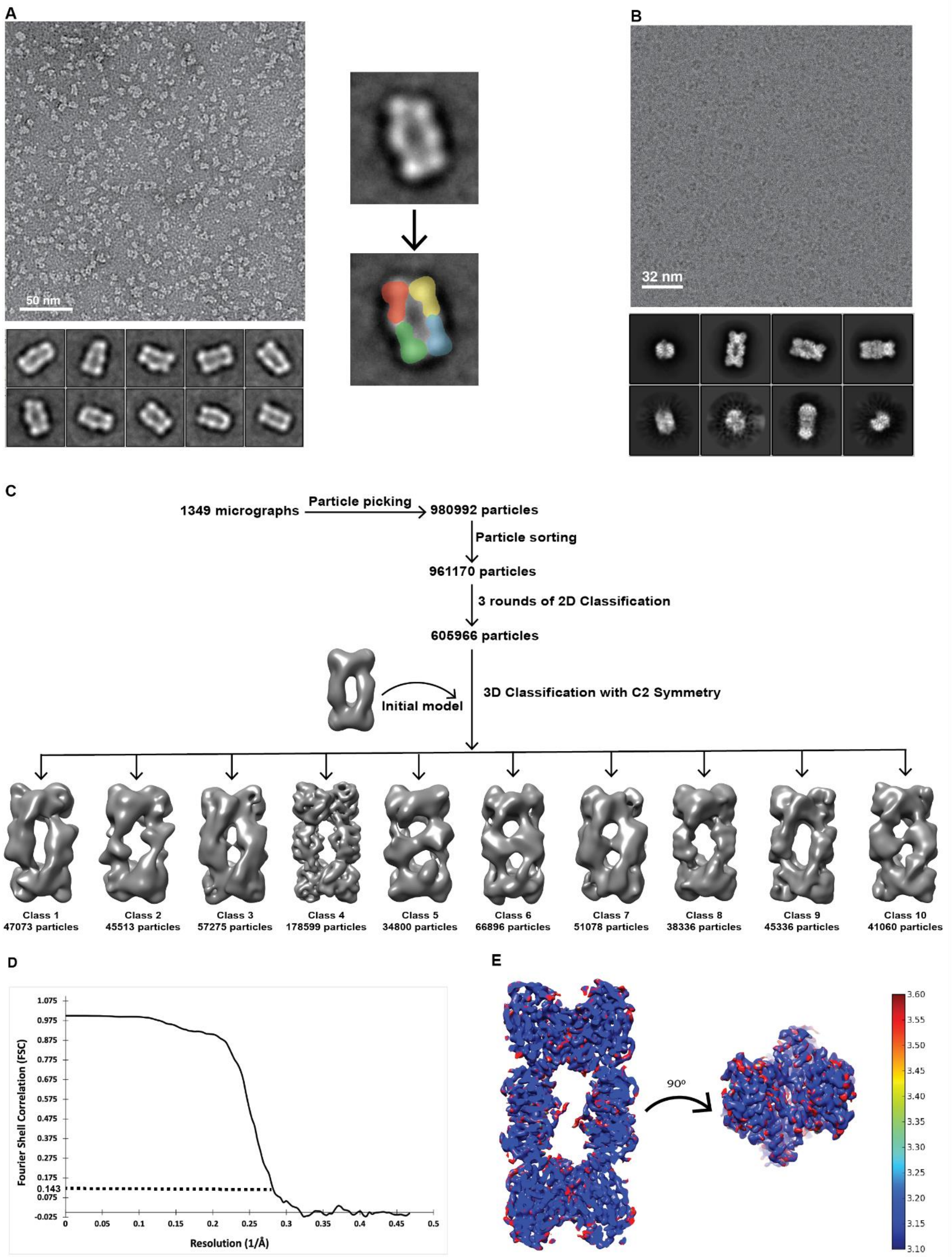
A
50 nm
B
32 nm
C
1349 micrographs
Particle picking
980992 particles
Particle sorting
961170 particles
3 rounds of 2D Classification
605966 particles
Initial model
3D Classification with C2 Symmetry
Class 1
47073 particles
Class 2
45513 particles
Class 3
57275 particles
Class 4
178599 particles
Class 5
34800 particles
Class 6
66896 particles
Class 7
51078 particles
Class 8
38336 particles
Class 9
45336 particles
Class 10
41060 particles
D
Fourier Shell Correlation (FSC)
Resolution (1/Å)
0.143
E
90º
3.60
3.55
3.50
3.45
3.40
3.35
3.30
3.25
3.20
3.15
3.10

Figure S4: Representative micrograph and 2D classification, pipeline of data processing using single particle cryo-EM, FSC and local resolution calculation of native *Mtb*Cbs

(A) A representative negative staining micrograph and reference free 2D class averages of native *Mtb*Cbs protein. 2D class averages indicate rectangular elongated native tetrameric *Mtb*Cbs protein. Particles are homogenously distributed (bottom panel). Enlarged view of 2D classification (right panel). Four different monomers are colored in red, blue, yellow, and green (right panel). **(B)** A representative cryo-EM micrograph and reference free 2D classification of native *Mtb*Cbs. 2D class averages indicate different orientations of protein and particles are homogeneously distributed (bottom panel). **(C)** Cryo-EM data processing workflow and structure determination of native *Mtb*Cbs. Detailed procedures are described in Method section. Class 4 having high resolution features were further refined and sharpened. **(D)** Gold standard Fourier Shell Correlation (FSC) curve of the high resolution cryo-EM map of native *Mtb*Cbs calculated at 0.143. **(E)** Local resolution calculation of native *Mtb*Cbs using ResMap.

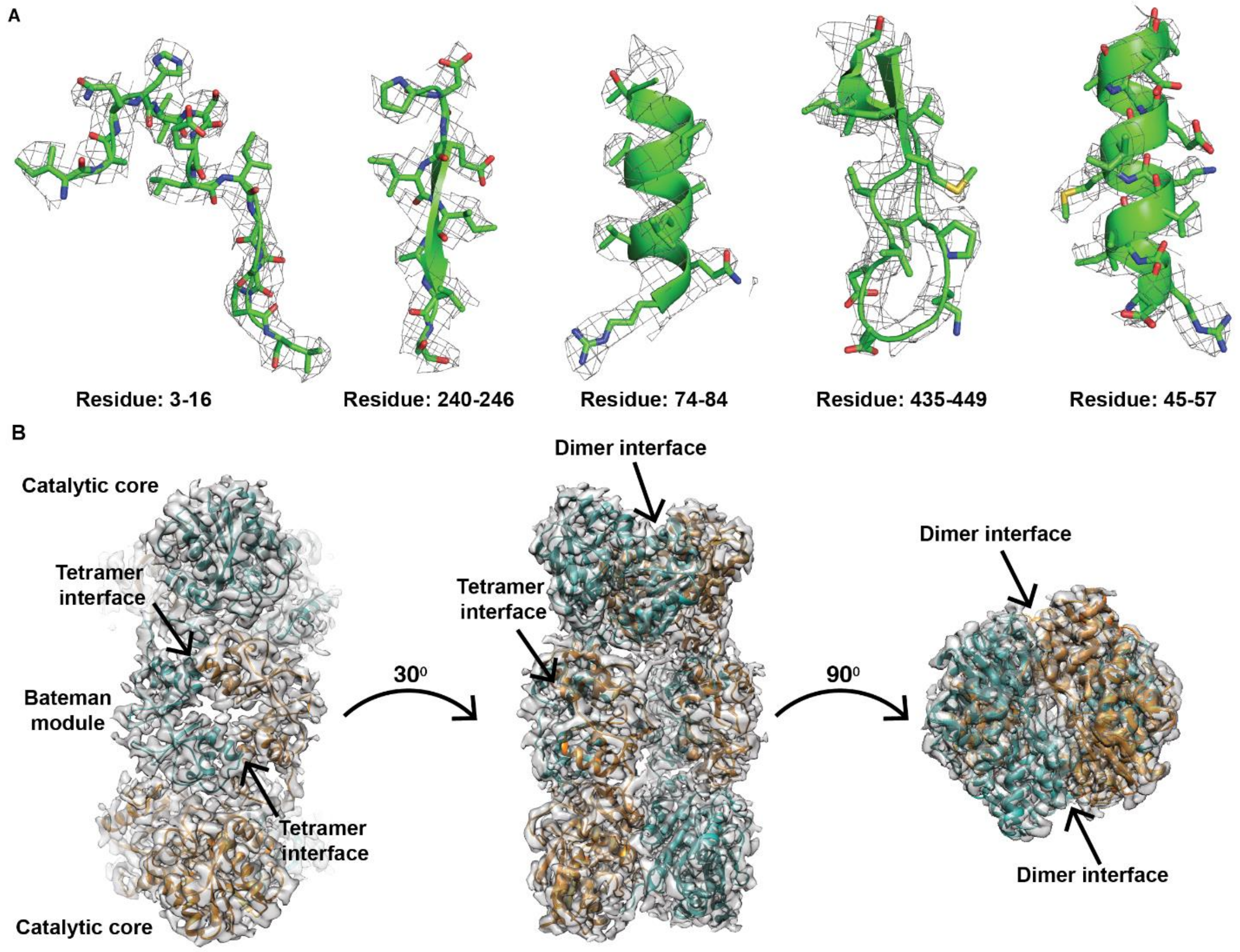

Figure S5: Side chain fitting and Identification of dimeric and tetrameric interface of *Mtb*Cbs:

(A) Side chain fitting at different regions of native *Mtb*Cbs shows proper fitting of amino acid residues in the cryo-EM map. **(B)** Arrangement of four monomers of *Mtb*Cbs is shown in two different colours, green and orange, where the diagonally opposite monomers have the same colour. Dimer and tetramer interfaces are marked with black arrowheads. Bateman modules from two monomers interact anti-parallelly to form a tetrameric *Mtb*Cbs.

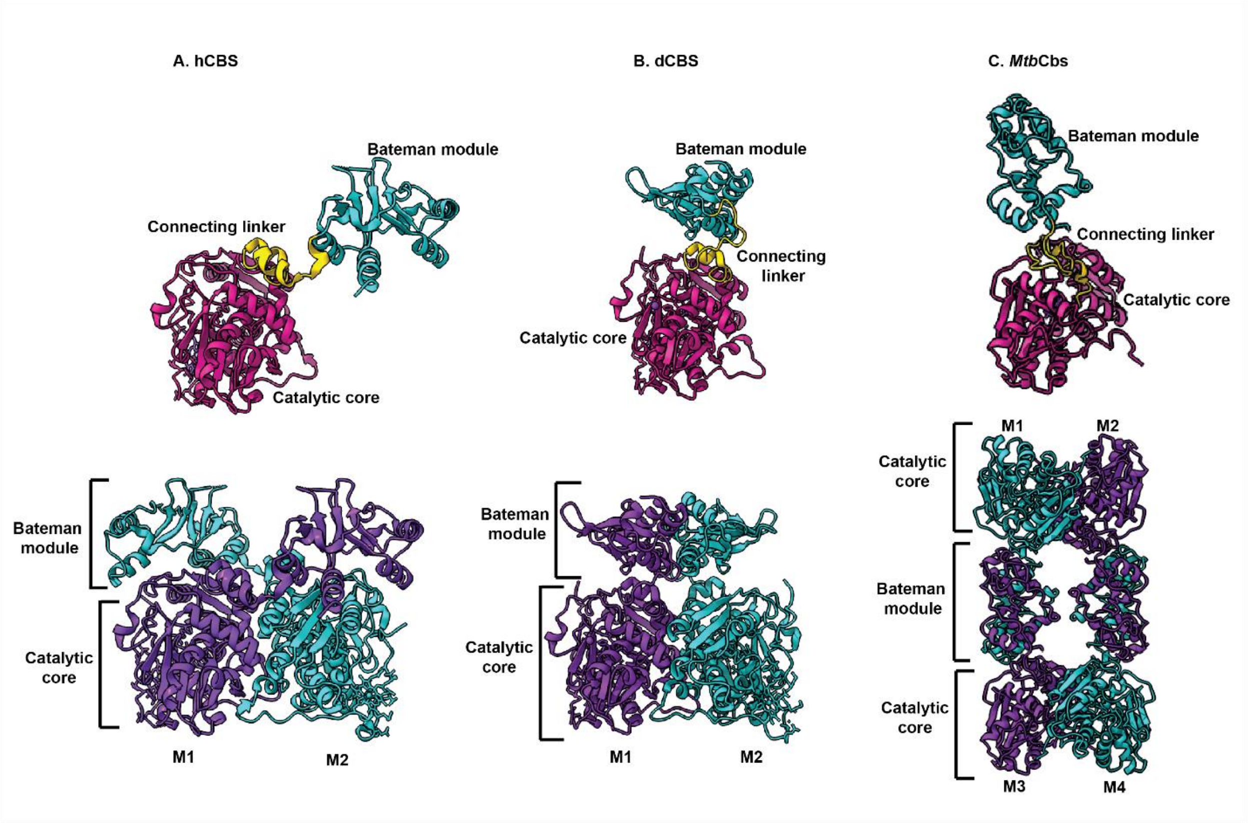

Figure S6: Comparison between *Mtb*, human and *Drosophila* CBS.

(A) Monomeric (upper panel) and dimeric (lower panel) representation of hCBS (human). **(B)** Monomeric (upper panel) and dimeric (lower panel) representation of dCBS (*Drosophila*). **(C)** Monomeric (upper panel) and tetrameric (lower panel) representation of *Mtb*Cbs. Catalytic core and Bateman module are positioned perpendicular to each other.

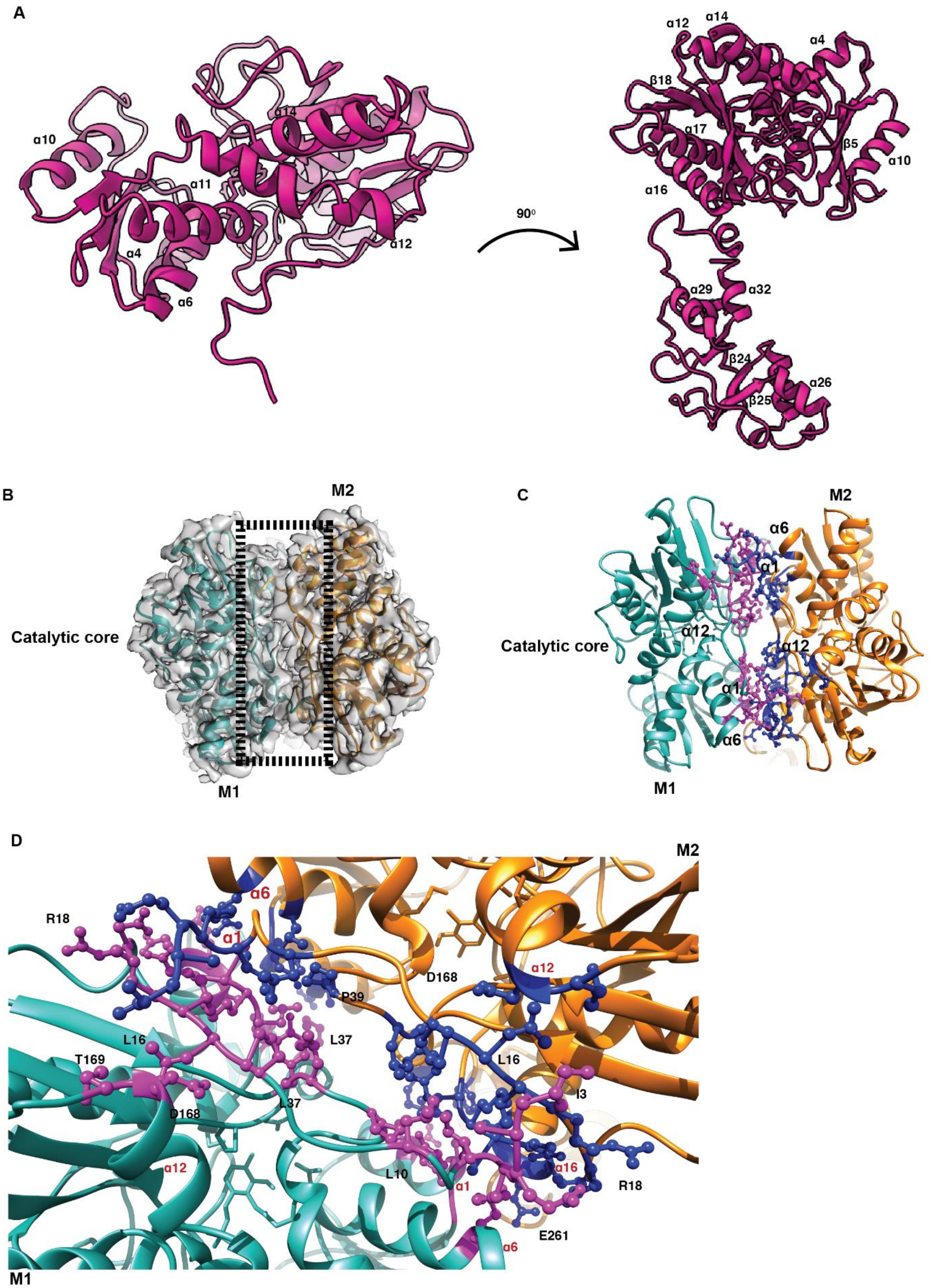

A
α10
α14
α11
α4
α6
α12
90⁰
α12
α14
α4
β18
α17
β5
α10
α16
α29
α32
β24
α26
β25
B
M2
Catalytic core
M1
C
M2
α6
α1
α12
Catalytic core
α12
α1
α6
M1
D
M2
R18
α6
α1
D168
α12
P39
L37
L16
T169
L16
I3
D168
L37
α12
L10
α1
α16
R18
E261
α6
M1

Figure S7: Identification of helices and beta strands, and dimeric interface of *Mtb*Cbs.

(A) Total 19 helices and 13 beta strands are present in the monomeric *Mtb*Cbs. Helices and beta strands are marked in different orientation of monomeric *Mtb*Cbs. **(B-C)** The dimeric interface at the catalytic core where two monomers (marked in green and orange) interact with each other. Transparent representation (left panel) and atomic model (right panel) shows the arrangement of two monomers during dimer formation. Amino acids responsible for the strong hydrophobic interactions at the dimer interface are marked in magenta and blue respectively (right panel). **(D)** Enlarged view of dimer interface where interacting helices and amino acid residues are marked.

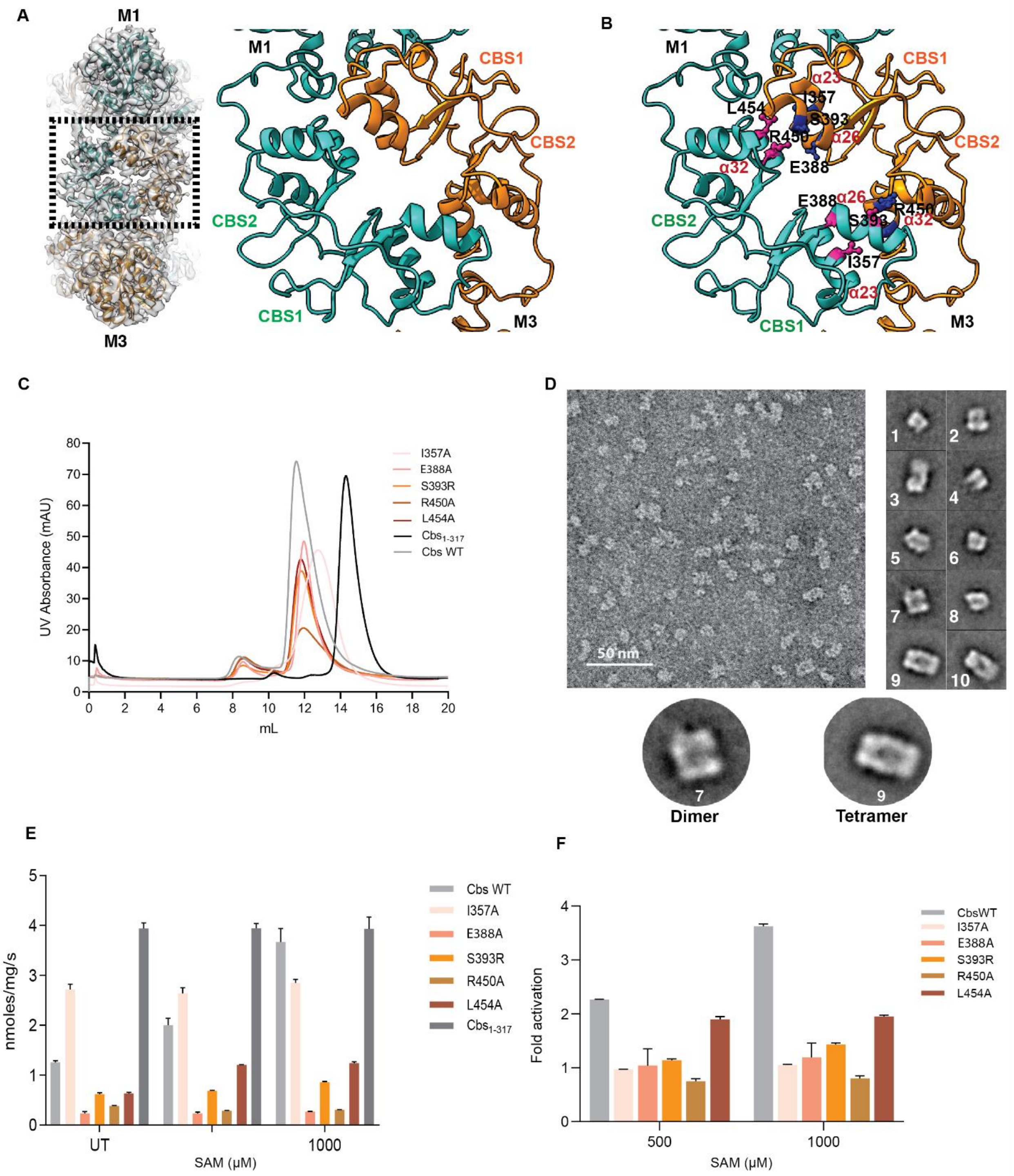

Figure S8: Tetrameric interfaces of *Mtb*Cbs and mutation studies at tetrameric interface.

(A) Transparent representation of two monomers' interaction at the Bateman module during tetramer formation (left panel). Two different monomers are colored in green and orange, respectively. Enlarged view of the Bateman module of two monomers (right panel). **(B)** Enlarged view of the atomic model of Bateman module. Amino acids (I357, E388, S393, R450 and L454) were selected for further mutational study. **(C)** Size exclusion chromatography (SEC) profile of all the mutated proteins, native *Mtb*Cbs and *Mtb*$Cbs_{1\text{-}317}$. **(D)** Negative staining micrograph of SEC purified I357A mutant (left panel). Reference free 2-D classification clearly indicates the mutated protein is a mixture of dimer and tetramer (right panel). Enlarged view of 2-D class averages explains the dimer and tetrameric arrangement of the protein (bottom panel). **(E)** Basal specific activity of wild type *Mtb*Cbs and mutants in presence of indicated amounts of SAM using 20 mM Cys. Data represent mean±SD of two independent biological replicates. **(F)** Measurement of fold activation of wild type *Mtb*Cbs and mutants in the presence of indicated amounts of SAM and 20 mM Cys. Fold activation was calculated by dividing specific activity in presence of SAM by specific activity in absence of SAM. Data represent mean ± SD of two independent biological replicates.

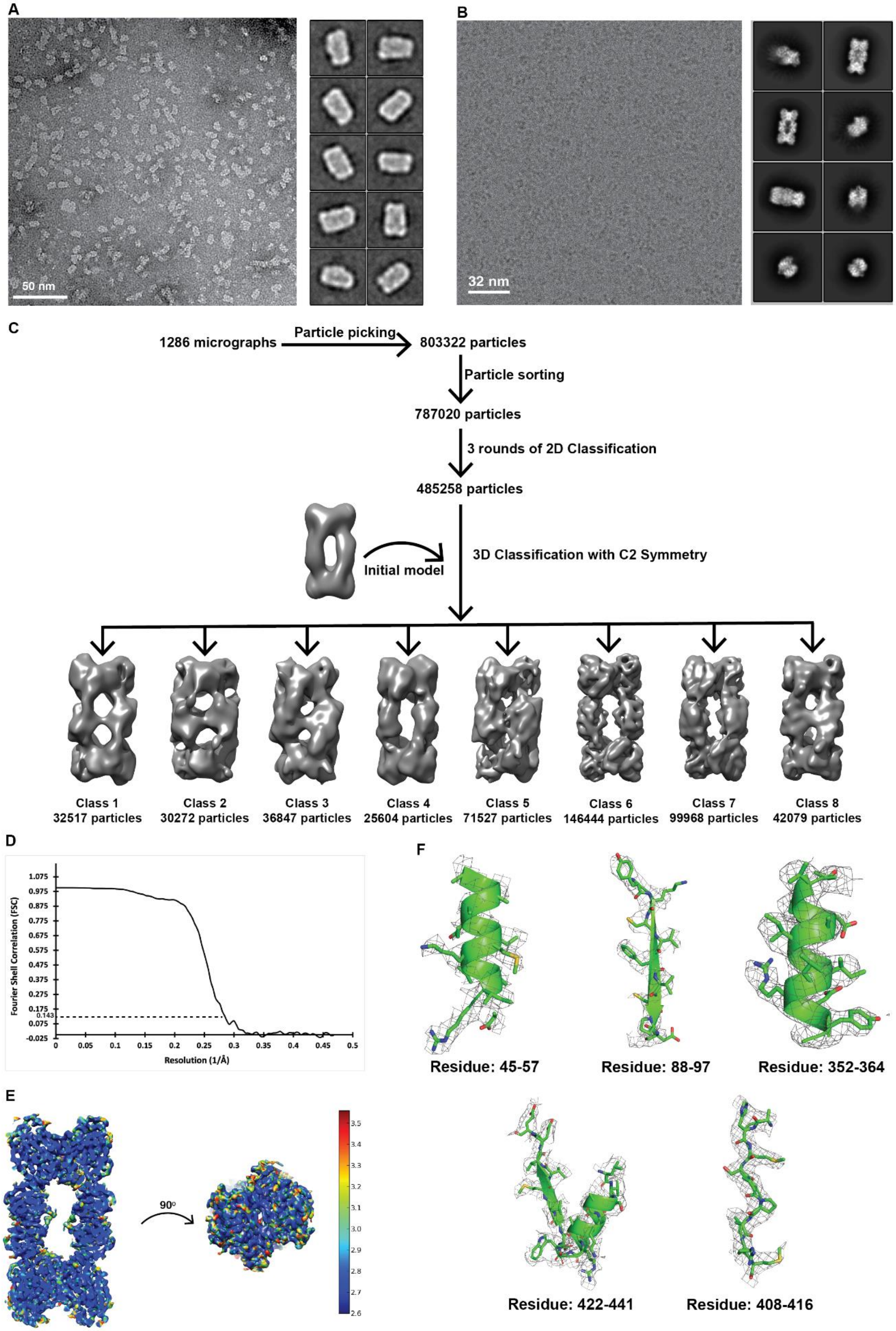
A
50 nm
B
32 nm
C
1286 micrographs
Particle picking
803322 particles
Particle sorting
787020 particles
3 rounds of 2D Classification
485258 particles
Initial model
3D Classification with C2 Symmetry
Class 1
32517 particles
Class 2
30272 particles
Class 3
36847 particles
Class 4
25604 particles
Class 5
71527 particles
Class 6
146444 particles
Class 7
99968 particles
Class 8
42079 particles
D
Fourier Shell Correlation (FSC)
Resolution (1/Å)
0.143
E
90°
F
Residue: 45-57
Residue: 88-97
Residue: 352-364
Residue: 422-441
Residue: 408-416

Figure S9: Representative micrograph and 2D classification, pipeline of data processing using single particle cryo-EM, FSC and local resolution calculation of SAM treated *Mtb*Cbs

(A). A representative negative staining micrograph and reference free 2D class averages of *Mtb*Cbs in the presence of SAM. 2D class averages indicate rectangular elongated protein particle similar to native *Mtb*Cbs. **(B)** A representative cryo-EM micrograph and reference free 2D class averages of *Mtb*Cbs in the presence of SAM. 2D class averages are very similar to native *Mtb*Cbs. **(C)** Cryo-EM data processing workflow and structure determination of *Mtb*Cbs in the presence of SAM. Detailed procedures are described in Method section. Class 6 having high resolution features were further refined and sharpened. **(D)** Gold standard Fourier Shell Correlation (FSC) curve of the high resolution cryo-EM map of SAM treated *Mtb*Cbs at 0.143. **(E)** Local resolution calculation of SAM treated *Mtb*Cbs using ResMap. **(F)** Side chain fitting at different regions of SAM treated *Mtb*Cbs shows proper fitting of amino acid residues in the cryo-EM map.

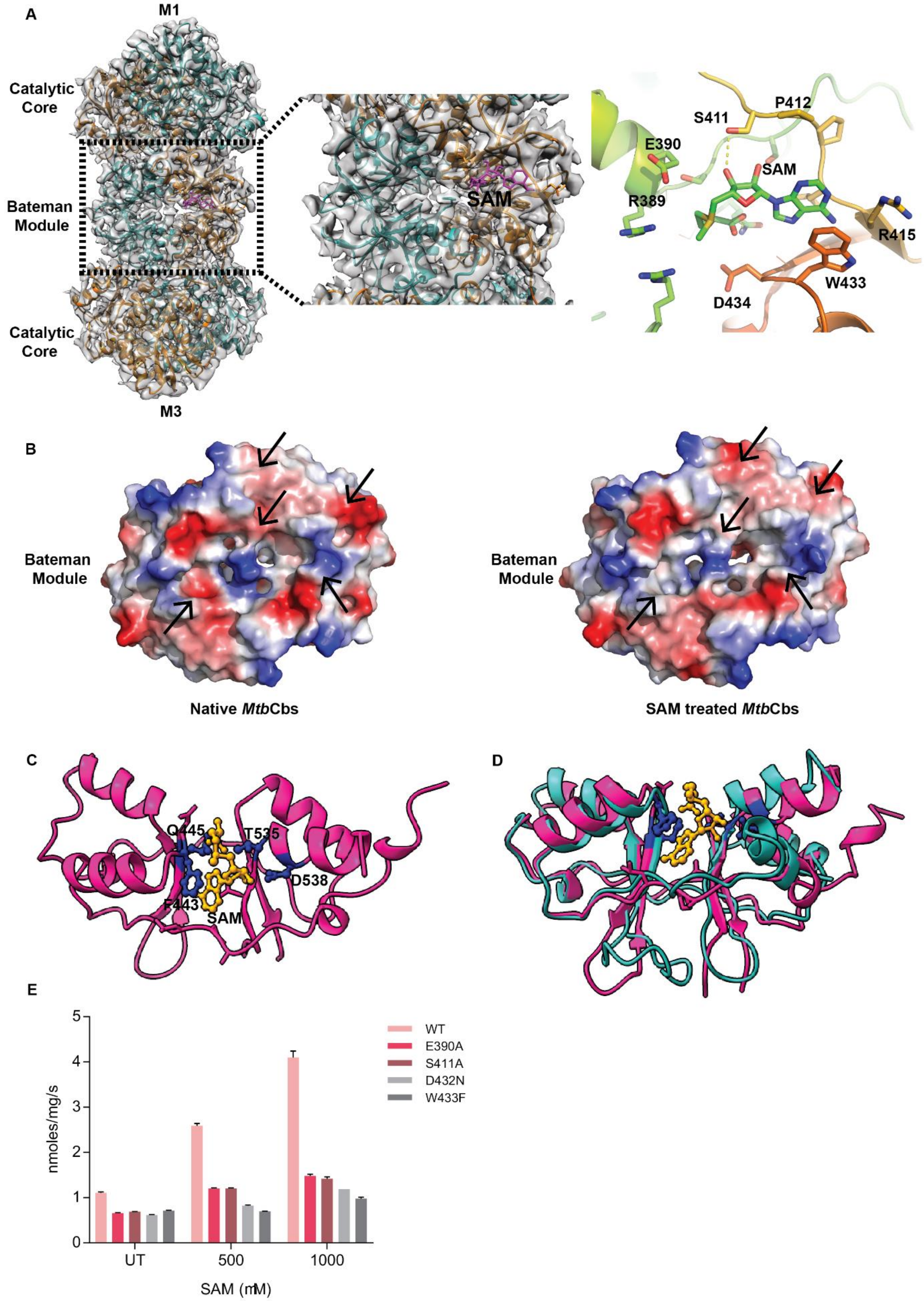
A
M1
Catalytic Core
Bateman Module
Catalytic Core
M3
SAM
S411
P412
E390
SAM
R389
R415
W433
D434
B
Bateman Module
Native MtbCbs
Bateman Module
SAM treated MtbCbs
C
Q445
T535
D538
F443
SAM
D
E
nmoles/mg/s
SAM (mM)
UT
500
1000
WT
E390A
S411A
D432N
W433F

Figure S10: SAM interaction at *Mtb*Cbs Bateman module, superimposition of *Mtb*Cbs and hCBS Bateman modules, and SAM mutants show impaired SAM mediated allosteric activation.

A. Transparent representation of *Mtb*Cbs treated with SAM (left panel). SAM bonding region is marked with black dotted box. Enlarged view of Bateman module (centre). SAM molecule is positioned at the predicted place. Predicted amino acids responsible for SAM interaction shown (right panel). **B.** Representation of electrostatic surface potential of Bateman module before and after SAM treatment. Significant redistribution of surface charges at different regions of Bateman module are marked with black arrowhead. **(C)** Key amino acids, F443, T535, Q445, and D538, responsible for SAM interaction for hCBS is marked in the regulatory domain (PDB ID: 4PCU). **(D)** Superimposition of hCBS regulatory and *Mtb*Cbs regulatory domain (atomic model). hCBS is marked in pink and *Mtb*Cbs is marked in blue. **(E)** Specific activity of *Mtb*Cbs and mutants in presence of the indicated amounts of SAM and 20 mm Cys as substrate. Data represent mean±SD of two independent biological replicates.

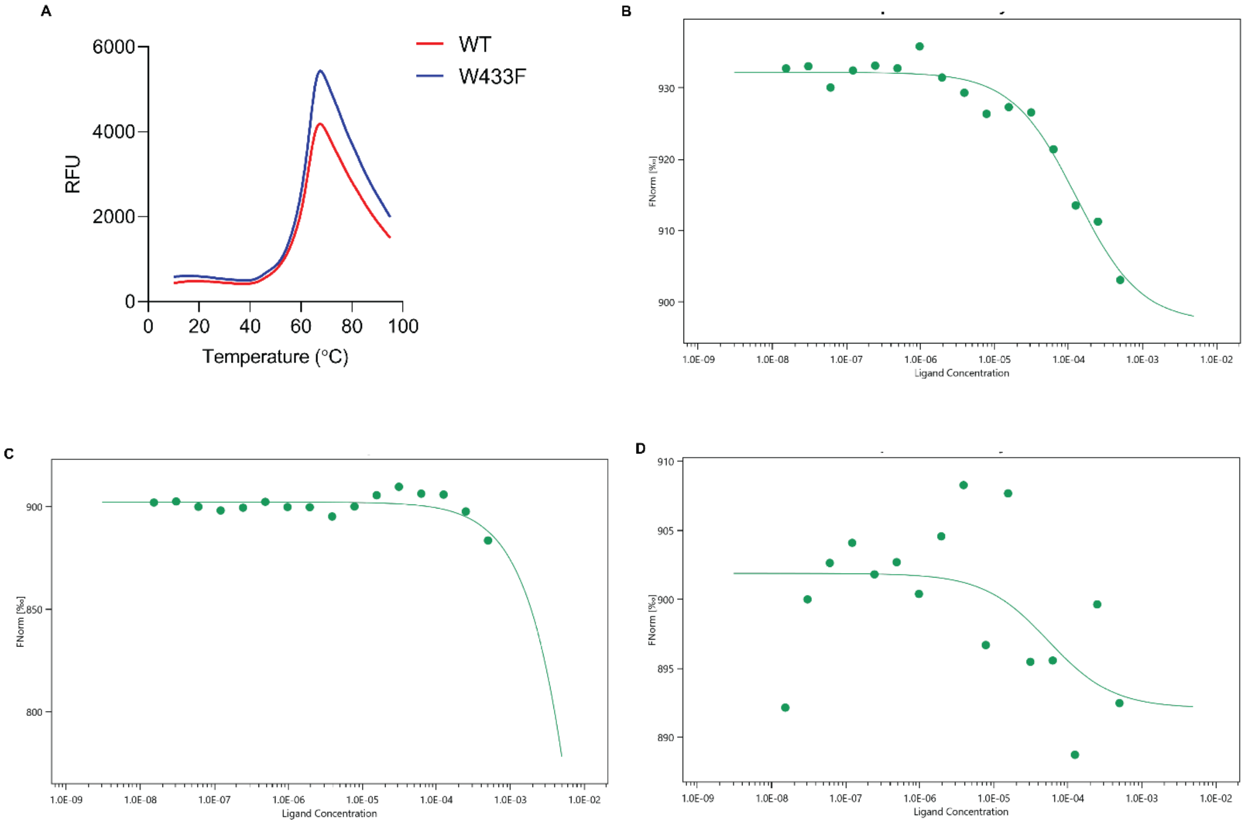

Figure S11: Stability and SAM binding affinity of WT and *Mtb*Cbs mutants. (A) Fluorescence thermal shift assay of WT *Mtb*Cbs and W433F mutant was performed using 10 µg of purified protein and SYPRO^{TM} orange dye. Dye binding to the proteins was measured over a range of temperatures to determine the T_m. SAM binding to purified **(B)** wild type *Mtb*Cbs, **(C)** W433F mutant and **(D)** *Mtb*Cbs$_{1\text{-}317}$ was evaluated using microscale thermophoresis. For this assay, the final protein concentration used was 100 nM. The protein was labelled with 100 nM red-Tris-NTA dye in the C-terminus of the His-tag. Monolith NT.115 capillaries were used in each experiment. In the binding assay, the protein concentration for both native and mutated, were kept constant. Both the protein was incubated with 16 two-fold serial dilutions of the ligand, SAM. The ligand was

solubilized in protein containing buffer (50 mM HEPES pH 7.4, 150 mM NaCl and 3% glycerol). The starting concentration for the ligand, SAM was 500 µM for all the cases.

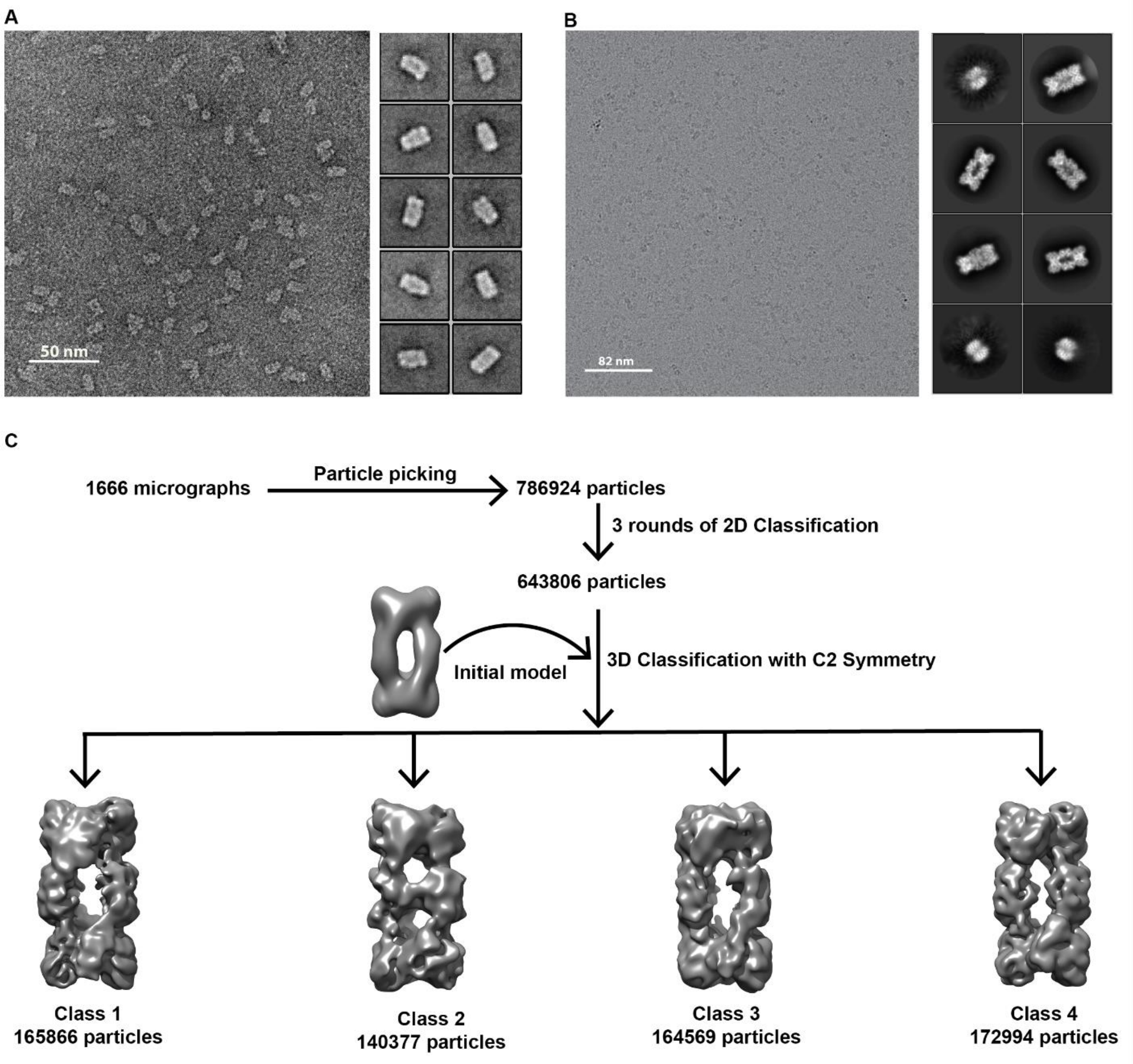

Figure S12: Representative micrograph and 2D classification, pipeline of data processing using single particle cryo-EM of SAM and Ser treated *Mtb*Cbs

(A) A representative negative staining micrograph and reference free 2D class averages of *Mtb*Cbs in presence of both SAM and Ser. 2D class averages indicate *Mtb*Cbs retains its rectangular tetrameric arrangement after substrate treatment. **(B)** A representative cryo-EM micrograph and reference free 2D class averages of *Mtb*Cbs in the presence of both SAM and Ser. **(C)** Cryo-EM data processing workflow and structure determination

of *Mtb*Cbs in presence of both SAM and Ser. Detailed procedures are described in Method section. Class 4 having high resolution features were further refined and sharpened.

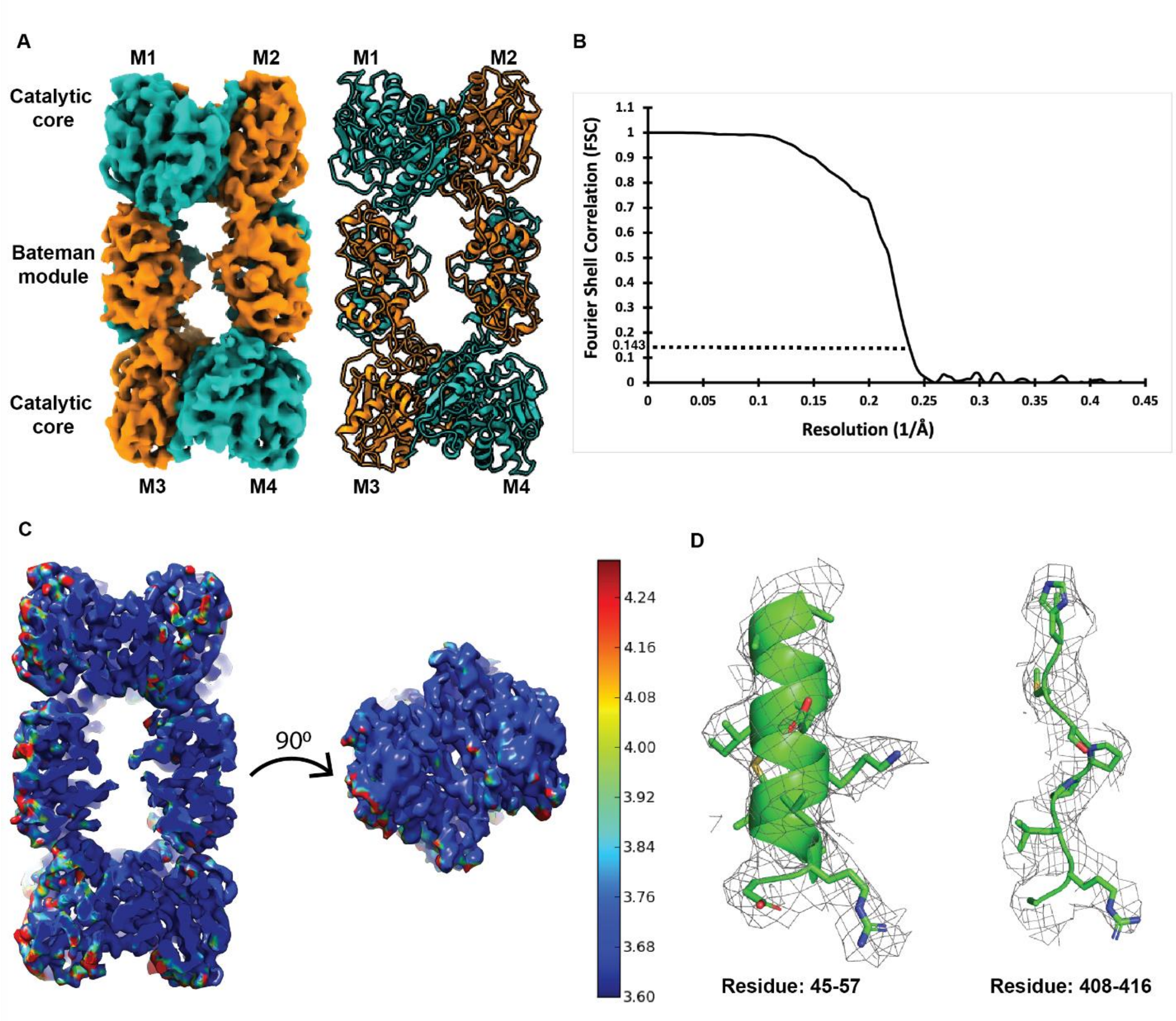

Figure S13: Cryo-EM map, atomic model, FSC and local resolution calculation of *Mtb*Cbs in the presence of both SAM and Ser.

(A) A solid representation of the cryo-EM 3D model of *Mtb*Cbs in the presence of both SAM and Ser. The arrangement of four monomers of *Mtb*Cbs is shown in two different colours, green and orange, where the diagonally opposite monomers have the same colour. Arrangement of catalytic core and Bateman module are marked, which indicates a similar structural arrangement to native *Mtb*Cbs. Atomic model of tetrameric *Mtb*Cbs in the presence of both SAM and serine. Four monomers in the atomic model are shown in

two different colours, green and orange, where diagonally opposite monomers have the same colour. **(B)** Gold standard Fourier Shell Correlation (FSC) curve of the cryo-EM map of SAM and Ser treated *Mtb*Cbs at 0.143. **(C)** Local resolution calculation of the map using ResMap. **(D)** Side chain fitting at different regions of the map shows the proper fitting of amino acid residues in the cryo-EM map.

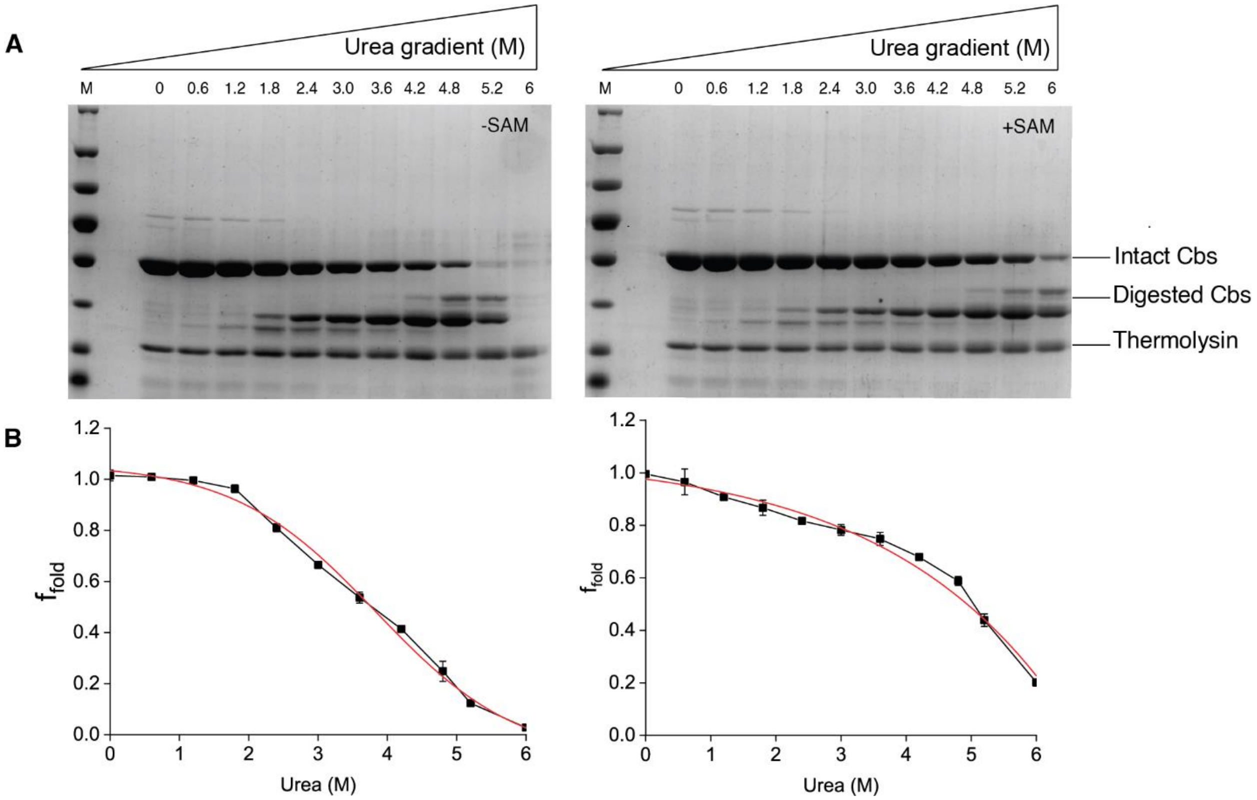

Figure S14: *In vitro* stabilization of *Mtb*Cbs by SAM

(A) Proteolytic digestion profile of WT *Mtb*Cbs in the absence and presence of 500 µM SAM in a denaturing urea gradient. **(B)** Dependence of the fraction of the folded protein (f_{fold}) on urea concentration in absence and presence of 500 µM SAM. Data represent mean ± SD of two independent biological replicates. f_{fold} was calculated by dividing the intensity of the intact protein band at a specific urea concentration by that of the untreated control (0 M urea).

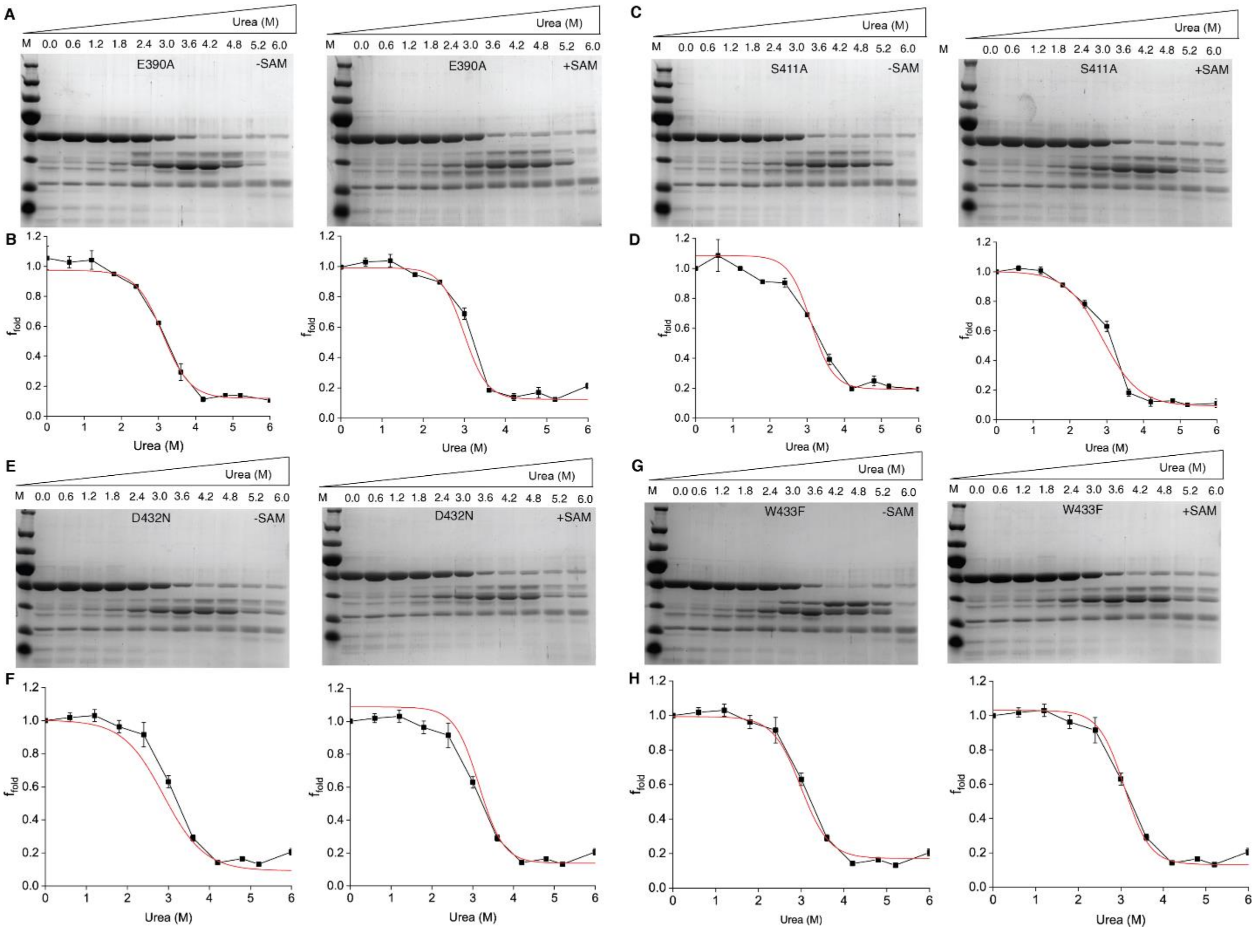

Figure S15: Pulse proteolysis assay of SAM binding deficient mutants.

Proteolytic digestion profile and dependence of fraction of folded protein (f_{fold}) on urea gradient of the SAM binding deficient mutant E390A **(A-B)** S411A **(C-D)** D432N **(E-F)** and W433F **(G-H)** in absence and presence of of 500 µM SAM in a denaturing urea gradient. Data represent mean ± SD of two independent biological replicates.

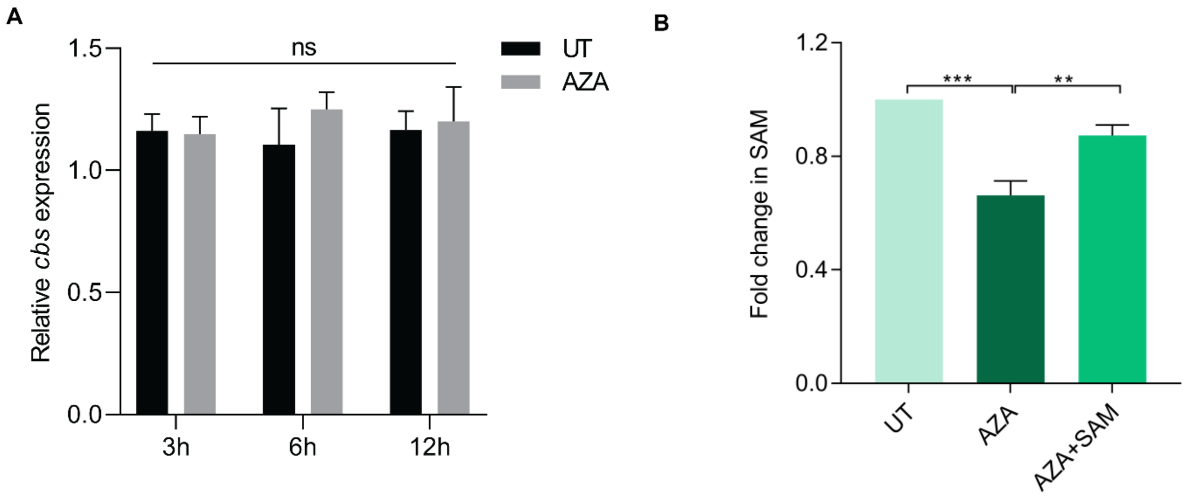

Figure S16: Effect of AZA on *Mtb*Cbs transcript and measurement of intracellular SAM upon exogenous addition. (A) RT-qPCR analysis of the transcript level of *cbs* upon AZA treatment for the indicated time periods. Relative expression was calculated by dividing treated by untreated values. Data represent mean ± SD of two independent biological replicates. 16s rRNA was used as internal control. **(B)** Fold change in abundance of SAM of WT *Mtb* upon treatment with 1 mM AZA for the 3 h followed by 1 mM SAM for additional 3 h. Data represent mean ± SD of three independent biological replicates. **p=0.0045, ***p = 0.003 by unpaired t-test.

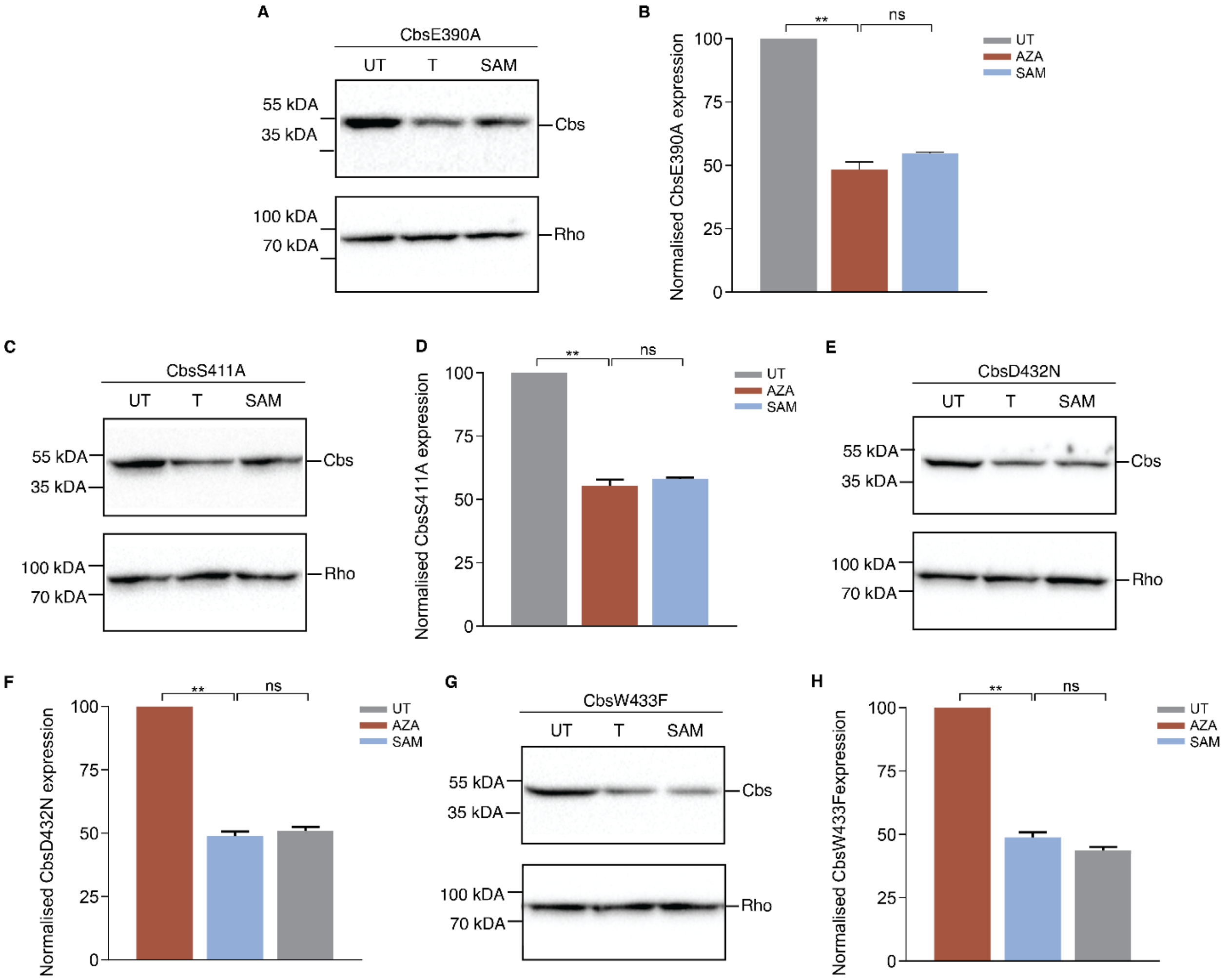

Figure S17: SAM fails to stabilize SAM binding deficient mutants of *Mtb*Cbs *in vivo*. Immunoblotting of mutants showing protein abundance upon treatment with 1 mM AZA for 3 h followed by addition of 1 mM SAM for additional 3 h and the corresponding densitometry profile. E390A **(A-B)**, S411A **(C-D)**, D432N **(E-F)** and W433F **(G-H)**. Cbs expression was normalized to the expression of the internal control Rho. Data represent mean±SD of two independent biological replicates. ns = not significant. **p = 0.0033 by unpaired t-test

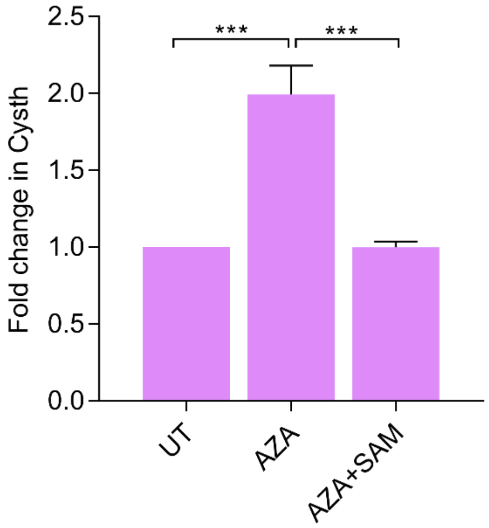

Figure S18: Induction of the FTS pathway is reversed by the exogenous addition of SAM. Fold change in abundance of Cysth of WT *Mtb* upon treatment with 1 mM AZA for the 3 h followed by 1 mM SAM for additional 3 h. Data represent mean ± SD of three independent biological replicates. ***p = 0.0008 by unpaired t-test.

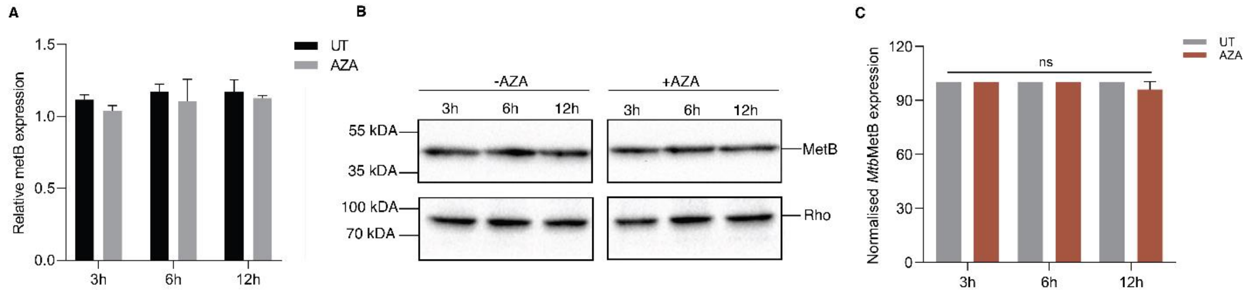

Figure S19: MetB transcript and protein levels remain unchanged upon AZA treatment. (A) RT-qPCR analysis of the transcript level of *metB* upon AZA treatment for the indicated time periods. Relative expression was calculated by dividing treated by untreated values. Data represent mean ± SD of two independent biological replicates. 16s rRNA was used as internal control. **(B)** Immunoblotting of MetB showing protein abundance upon treatment with 1 mM AZA for the indicated time periods and **(C)** the corresponding densitometry profile. MetB expression was normalized to the expression of the internal control Rho. Data represent mean ± SD of two independent biological replicates. ns = not significant.

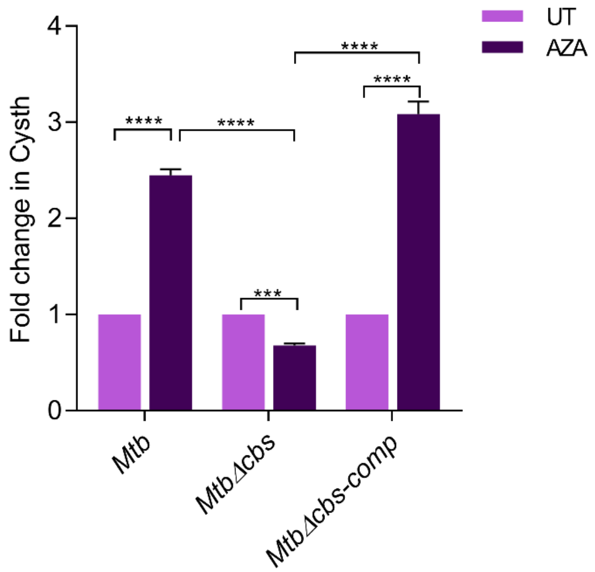

Figure S20: *MtbΔcbs* is unable to trigger the FTS pathway upon AZA treatment. Fold change in abundance of Cysth upon treatment with 1 mM AZA for 6 h. Comp – WT *Mtb*Cbs complemented strain. Data represent mean ± SD of three independent biological replicates. ***p=0.0002, ****p = 0.0001 by two-way ANOVA with Tukey's multiple comparison test.

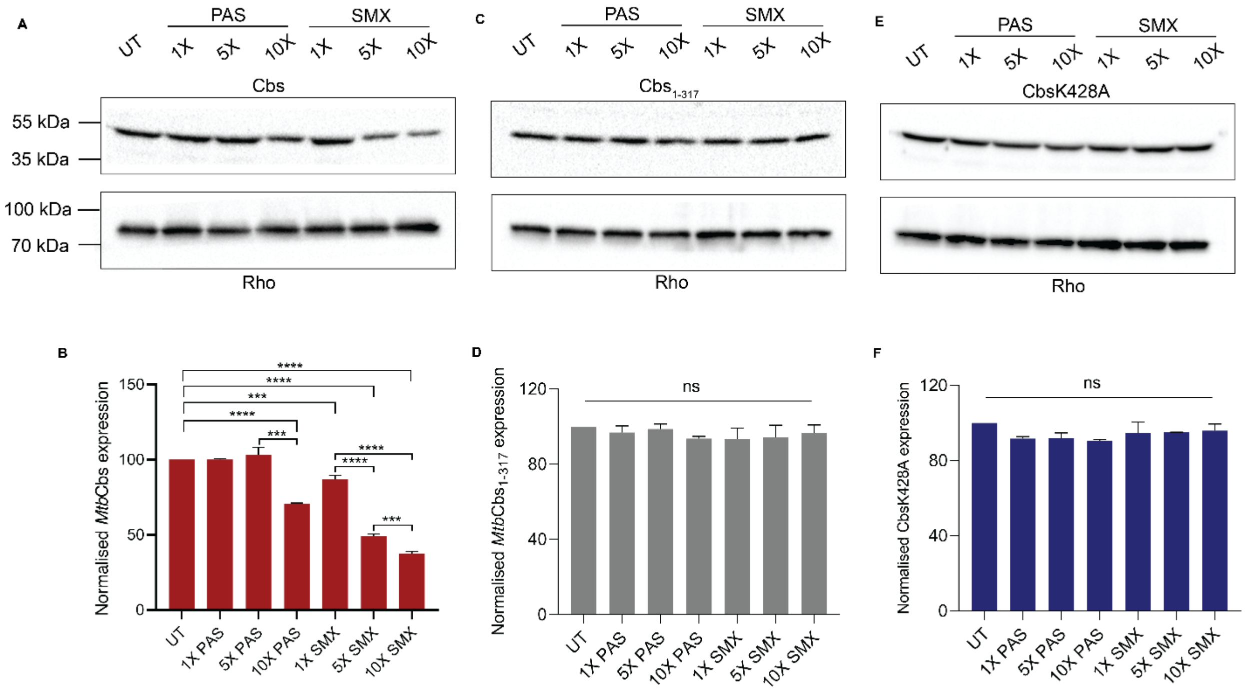

Figure S21: Antifolate drugs perturb the stability of *Mtb*Cbs.

Immunoblotting of **(A)** WT *Mtb* (**C**) *Mtbcbs1-317* and (**E**) *MtbcbsK428A* showing protein abundance upon treatment with upon treatment with PAS and SMX for 12 h and their corresponding densitometry profile (**B**), (**D**) and (**F**) respectively. Cbs expression was normalized to the expression of the internal control Rho. Data represent mean ± SD of two independent biological replicates. ***p =0.001, ****p<0.0001 by one-way ANOVA with Tukey's multiple comparison. ns = not significant.

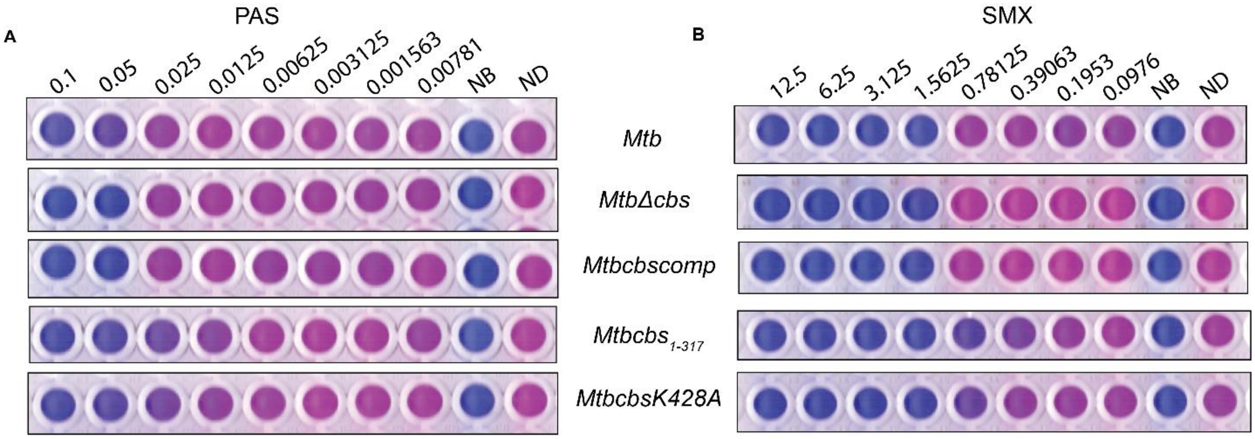

C

Strain	PAS MIC$_{90}$ µg/mL	SMX MIC$_{90}$ µg/mL
Mtb	0.05	1.5625
MtbΔcbs	0.05	1.5625
Mtbcbscomp	0.05	1.5625
Mtbcbs$_{1-317}$	0.025	0.39063
MtbcbsK428A	0.025	0.78125

Figure S22: Antifolate susceptibility of WT *Mtb*, *MtbΔcbs*, *Mtbcbscomp*, *Mtbcbs$_{1-317}$* and *MtbcbsK428A*

Minimum Inhibition Concentration with 90% inhibition (MIC$_{90}$) of para-amino salicylic acid (PAS) and sulfamethoxazole (SMX) was calculated using resazurin microplate assay (REMA). Comp – WT *Mtb*Cbs complemented strain Data is representative of two independent biological replicates. NB = no bacteria control, ND = no drug control.

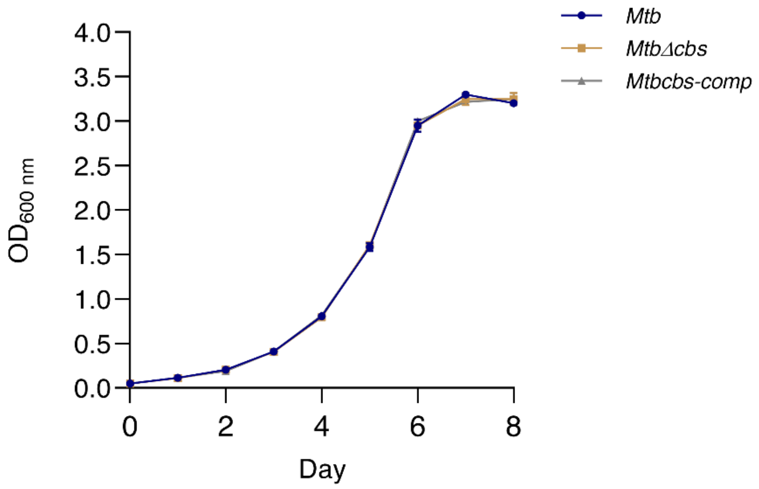

Figure S23: Deletion of *Mtb*Cbs does not lead to Cys auxotrophy. Growth curve of WT *Mtb*, *MtbΔcbs* and *Mtbcbs-comp* in 7H9 media without Cys supplementation. Data represent mean±SD of two independent biological replicates.

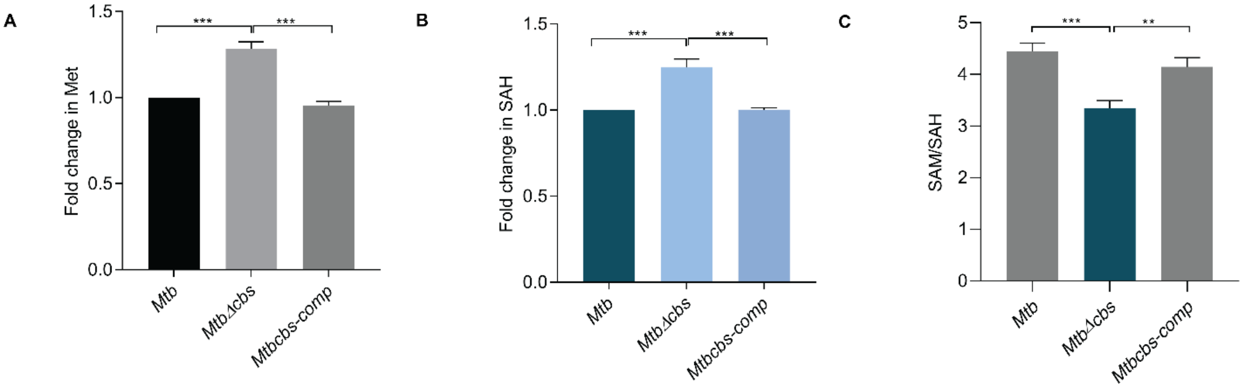

Figure S24: *Mtb*Cbs deletion perturbs abundance of AMC intermediates.

Fold change in metabolite profile of *MtbΔcbs* showing **(A)** Met and **(B)** SAH abundance as compared to WT *Mtb*. Comp – WT *Mtb*Cbs complemented strain Data represent mean±SD of three independent experiments. ***p=0.0003 by unpaired t-test **(C)** Deletion of *Mtb*Cbs leads to imbalance of the SAM/SAH ratio in *Mtb*. Data represent mean±SD of three independent experiments. **p=0.004, ***p = 0.0009 by unpaired t-test.

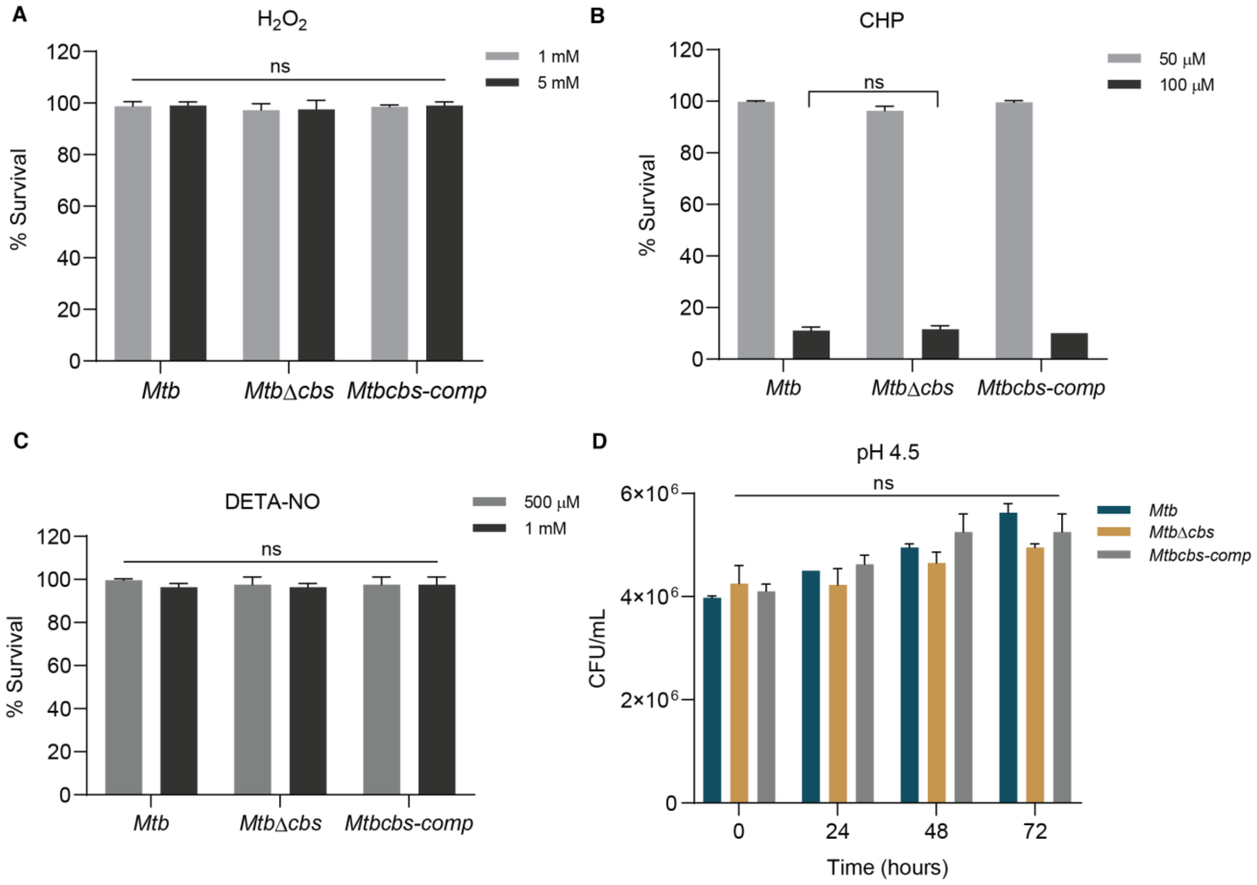

Figure S25: Deletion of *Mtb*Cbs does not lead to increased susceptibility towards commonly encountered stresses. Deletion of *Mtb*Cbs does not affect survival when exposed to oxidative stress using **(A)** H_2O_2, **(B)** CHP, nitrosative stress using **(C)** DETA-NO or **(D)** acidic pH for 24 h and percent survival was calculated by CFU enumeration. Comp – WT *Mtb*Cbs complemented strain. Data represent mean ± SD of three independent experiments. ns = not significant.

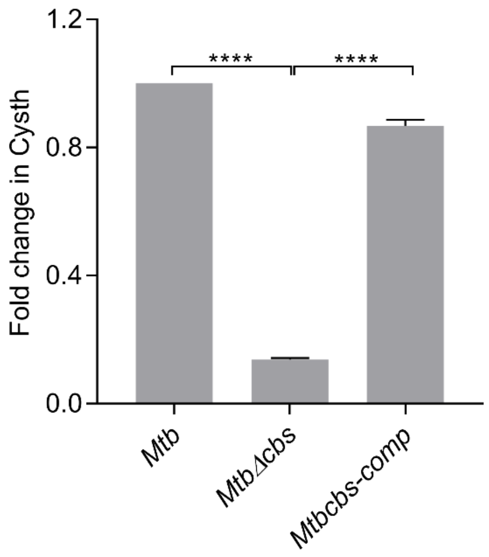

Figure S26: *Mtb*Cbs is the putative cystathionine synthase in *Mtb*. Metabolite profile of WT *Mtb*, *MtbΔcbs* and *Mtbcbs-comp* showing Cysth abundance of Cysth. Comp – WT *Mtb*Cbs complemented strain. Data represent mean ± SD of three independent experiments. ****$p < 0.0001$ by unpaired t-test.

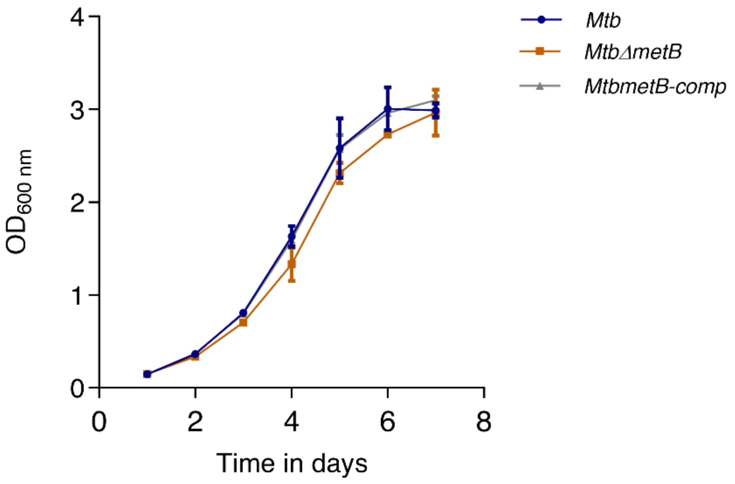

Figure S27: Deletion of MetB does not lead to Met auxotrophy. Growth curve of WT *Mtb*, *MtbΔcbs* and *Mtbcbs-comp* in 7H9 media without Met supplementation. Data represent mean ± SD of two independent biological replicates.

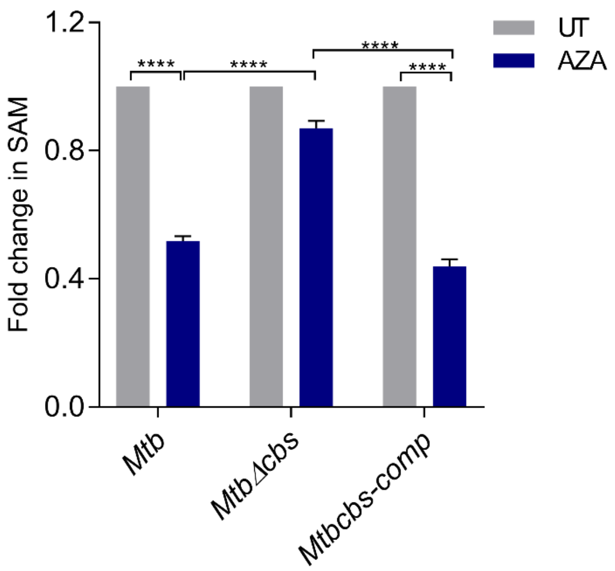

Figure S28: SAM levels remain relatively unchanged upon AZA treatment of *MtbΔcbs.* Metabolite profile of WT *Mtb*, *MtbΔcbs* and *Mtbcbs-comp* showing SAM abundance. Comp – WT *Mtb*Cbs complemented strain Data represent mean ± SD of three independent experiments. ****p < 0.0001 by one-way ANOVA with multiple comparison.

Table S1: Reactions catalyzed by hCBS

Serine + Homocysteine → Cystathionine + H_2O	β-replacement
Cysteine +H_2O → Serine + H_2S → Pyruvate + NH_3	α,β-elimination
Cysteine + Cysteine → Lanthionine + H_2S	β-replacement
Cysteine + Homocysteine → Cystathionine + H_2S	β-replacement
Homocysteine + H_2O → Homoserine + H_2S → α-ketobutyrate + NH_3	α,γ elimination

Table S2: Kinetic parameters of H_2S producing reactions of WT *Mtb*Cbs in absence and presence of SAM, and of *Mtb*Cbs$_{1-317}$.

Parameter	WT *Mtb*Cbs	WT *Mtb*Cbs + SAM	*Mtb*Cbs$_{1-317}$
1. Cys + Cys→ Lanthionine + H_2S			
k_m	43.64 ± 1.11 mM	43.8 ± 0.87 mM	41.3 ± 0.75 mM
V_{max}	3.2 ± 0.96 U/mg	8.01 ± 0.88 U/mg	10.87 ± 0.98 U/mg
K_{cat}	0.64 ± 0.12 s^{-1}	1.6 ± 0.13 s^{-1}	2.17 ± 0.12 s^{-1}
2. Hcys + Cys → Cystathionine + H_2S			
k_m (Hcys)	0.4 ± 0.13 mM	0.41 ± 0.21 mM	0.41 ± 0.34 mM
k_m (Cys)	32.72 ± 1.01 mM	30.5 ± 1.22 mM	29.7 ± 1.2 mM
V_{max}	8.11 ± 0.76 U/mg	31.2 ± 0.82 U/mg	33.4 ± 1.45 U/mg
k_{cat}	1.61 ± 0.72 s^{-1}	6.25 ± 0.68 s^{-1}	6.8 ± 0.77 s^{-1}

U = nmoles/s

The initial rate of Cys-directed H_2S formation followed a hyperbolic dependence on Cys, and the data were fitted to the Michaelis–Menten equation to obtain the kinetic parameters. Cysteine was varied from 10 mM - 50 mM. H_2S production from Cys was not biphasic in line with the LC-MS/MS data, which indicates the absence of the Ser production via β-elimination. H_2S production from Cys and Hcys follows bimolecular reaction kinetics. For this reaction, homocysteine was varied from 1 mM to 9 mM with cysteine held constant at 20 mM, and cysteine was varied from 10 mM - 50 mM with homocysteine held constant at 1 mM. We performed the reaction at a lower concentration range of Hcys as we observed significant substrate inhibition at higher concentrations. This data set was fitted to the equation of bimolecular kinetics to obtain the kinetic

parameters. For the bisubstrate reactions, data fits to ping-pong and sequential models were indistinguishable. Data represent two independent biological replicates.

Table S3: Cryo-EM data acquisition, processing parameters and refinement statistics

Parameters	Native *Mtb*Cbs (small dataset)	Native *Mtb*Cbs	SAM treated *Mtb*Cbs	Serine treated *Mtb*Cbs
**Microscope type**	Talos Arctica 200 kV cryo-EM	Titan Krios 300 kV cryo-EM	Titan Krios 300 kV cryo-EM	Talos Arctica 200 kV cryo-EM
**Camera**	K2 direct electron detector (DED)	Falcon 3 Direct Electron Detector (DED)	Falcon 3 Direct Electron Detector (DED)	K2 direct electron detector (DED)
**Electron gun**	Field emission gun	Field emission gun	Field emission gun	Field emission gun
**Voltage (HT)**	200 kV	300 kV	300 kV	200 kV
**Electron dose**	40 $e^{-}/Å^{2}$	30 $e^{-}/Å^{2}$	30 $e^{-}/Å^{2}$	40 $e^{-}/Å^{2}$
**Electron dose per frame**	2 $e^{-}/Å^{2}$	1.2 $e^{-}/Å^{2}$	1.2 $e^{-}/Å^{2}$	2 $e^{-}/Å^{2}$
**Total number of frames**	20	25	25	20
$Å/\boldsymbol{pix}$	1.17	1.07	1.07	1.17
**Defocus range**	-0.75 to -2.25 μm	-2.2 to -3.9 μm	-2.2 to -3.9 μm	-0.75 to -2.25 μm
**Plunge freeze instrument**	Vitrobot Mark IV	Vitrobot Mark IV	Vitrobot Mark IV	Vitrobot Mark IV
**Data collection mode**	Counting mode	Counting mode	Counting mode	Counting mode
**Symmetry imposed**	-	C2	C2	C2
**Number of movie files**	-	1349	1286	1666
**Number of particles in model**	-	178,599	146,444	172,994
**Map resolution (Å)**	-	3.6 Å	3.56 Å	4.25 Å
**FSC threshold**	-	0.143	0.143	0.143
**Map sharpening B factor ($Å^{2}$)**	-	-200	-200	-150
**MolProbity score**	-	3.05	3.01	3.30
**Poor rotamers (%)**	-	3.42	3.85	6.63
**Ramachandran plot**				
**Favored (%)**	-	87.72	89.47	89.84

**Allowed (%)**	-	12.01	10.53	9.73
**Outliers (%)**	-	0.27	0	0.44
**EM Ringer score**	-	2.53	2.43	2.16

Table S4: Denaturation and unfolding parameters of WT *Mtb*Cbs and mutants.

Name	C_m		$\Delta G_{unfold}^{\circ}$	
	-SAM	+SAM	-SAM	+SAM
WT *Mtb*Cbs	3.7 ± 0.3 M	4.92 ± 0.55 M	21.1± 0.2 $kcalmol^{-1}$	30.34 ± 0.91 $kcalmol^{-1}$
E390A	3.165 ± 0.4 M	3.06 ± 0.2 M	19.1 ± 0.3 $kcalmol^{-1}$	18.44 ± 0.11 $kcalmol^{-1}$
S411A	3.123 ± 0.21 M	3.29 ± 0.7 M	18.8 ± 0.9 $kcalmol^{-1}$	19.89 ± 0.15 $kcalmol^{-1}$
D432N	3.375 ± 0.25 M	3.195 ± 0.6 M	19.9 ± 0.6 $kcalmol^{-1}$	19.3 ± 0.2 $kcalmol^{-1}$
W433F	3.3 ± 0.52 M	3 ± 0.3 M	20 ± 0.6 $kcalmol^{-1}$	18 ± 0.2 $kcalmol^{-1}$

Data represent two independent biological replicates.

Table S5: Minimum Inhibitory Concentration (MIC) of anti-TB drugs (μg/mL)

Drug	Strain		
	WT *Mtb* $H_{37}Rv$	*MtbΔcbs*	*Mtbcbs-comp*
Isoniazid	0.125	0.125	0.125
Rifampicin	0.0625	0.0625	0.0625
Clofazimine	0.250	0.250	0.250

Data represent three independent biological replicates.
